# Generalized additive mixed models to discern data-driven theoretically informed strategies for public brain, cognitive and mental health

**DOI:** 10.1007/s10654-025-01296-9

**Published:** 2025-09-15

**Authors:** Laurenz Lammer, Frauke Beyer, Steffi Riedel-Heller, Julia Sacher, Heide Glaesmer, Arno Villringer, A. Veronica Witte

**Affiliations:** 1 Department of Neurology, Max Planck Institute for Human Cognitive and Brain Sciences, Stephanstraße 1a, 04103 Leipzig, Germany; 2 Clinic for Cognitive Neurology, University of Leipzig Medical Center, Leipzig, Germany; 3 Institute of Social Medicine, Occupational Health and Public Health, Faculty of Medicine, University of Leipzig, Leipzig, Germany; 4 Max Planck School of Cognition, Leipzig, Germany; 5 Leipzig Center for Female Health & Gender Medicine, Medical Faculty, University Clinic Leipzig, Leipzig, Germany; 6 Department of Medical Psychology and Medical Sociology, University of Leipzig, Leipzig, Germany; 7 Berlin School of Mind and Brain, Humboldt University of Berlin, Berlin, Germany

**Keywords:** Social isolation, Social connection, Brain health, Cognitive health, Dementia, Anxiety, Depression, Generalized additive mixed models, Public health, Epidemiology, Strategy of preventive medicine

## Abstract

Social isolation is recognized as a public health emergency. However, major guidelines provide vastly different recommendations on how to target it, and no strategy has been substantiated on firm theoretical or empirical grounds, yet. Rose’s seminal *The Strategy of Preventive Medicine* provided a theoretical framework for such arbitrations between approaches. Therein, determining the shape of the relationship between risk factor and outcome is of paramount importance. However, quantitative approaches immediately applying this theory to evidence are still lacking. Thus, in this pre-registered analysis, we pursued a novel approach and employed generalized additive mixed models to model the shape of social isolation’s Links to brain, cognitive and mental health outcomes in a well-characterised population-based sample. We derived brain measures from 3T MRIs, assessed cognitive functions with extensive neuropsychological testing and measured social isolation and mental health outcomes using established questionnaires. Overall, we studied over 10,000 (mean age 58a, 53% women) participants at baseline and over 5500 (mean age 64a, 53% women) at follow-up after ~ 6 years. The relationship of social contact with almost all outcomes was firmly linear and did not differ above and below the standard threshold for social isolation. Only for processing speed did we detect a steeper slope amongst socially isolated individuals. Hence, most of the health effects of social contact were observed in individuals that would not be categorised as socially isolated. Applying advanced statistical methods to a large and well-characterised dataset we provide evidence in support of a shift in focus away from individual-level and towards population-level preventive approaches.

**Supplementary Information:**

The online version contains supplementary material available at 10.1007/s10654-025-01296-9.

## Significance Statement

Poor social connection is a pressing public health concern but sound evidence on which strategy to pursue in preventive efforts is severely lacking. Here, we provide an unprecedented approach to quantitatively apply fundamental public health theory to discern an apt preventive strategy. This allowed us to show that current efforts focussing exclusively on socially isolated individuals are likely missing the bulk of the preventive potential obtainable by targeting social isolation.

**Supplementary Information:**

The online version contains supplementary material available at 10.1007/s10654-025-01296-9.

## Introduction

Poor social connection is recognized as one of the public health emergencies of our times. Since 2018, the governments of Japan and the UK initiated dedicated ministries [1], the US Surgeon General issued a report on the epidemic of social isolation and loneliness [2] and the German government published an official strategy against loneliness [3]. Two insights drove governments to be seriously concerned about social isolation, the lack of social contact, and loneliness, the dissatisfaction with one’s social Life. First, we realised that 25% of our elderly population are socially isolated [4] and one in two suffers from loneliness [5], highlighting the sheer magnitude of the problem. Second, evidence of the substantial adverse effects of social isolation and loneliness on wellbeing, morbidity and mortality amounted steadily [6].

Several theories have been proposed to explain the beneficial effects of social interactions. According to the stress-buffering hypothesis, they can be explained by social support’s potential to cushion the adverse effects of stress [7]. The main-effect theory postulates that social relationships foster beneficial health behaviours, affective states and endocrine and immunological responses [8]. Concerning cognitive decline, yet another theory may apply. The ‘use-it-ot-lose-it’ theory postulates that sustained cognitive activity, which is often stimulated in social interactions, is pivotal to maintain brain functioning as we age [9]. Lastly, reverse causation or simultaneity might also contribute to observed associations.

These mechanisms are likely to underly the associations of social isolation with the psychiatric ailments of dementia, anxiety and depressive disorders as social isolation is an important risk factor for them [10–16]. These diseases are all grave public health concerns in and of themselves. Already, dementias cause tremendous suffering and affect over 55 million individuals [17, 18]. By 2035, their prevalence is predicted to exceed 100 million [19]. Livingston et al. calculated that we could prevent up to 5% of dementia cases by averting social isolation, though [10]. For anxiety and depression, social isolation’s importance seems even greater as Li et al. estimated that 9 and 11% of cases are attributable to it, respectively [13]. Depressive and anxiety disorders are the two leading mental health contributors to the global burden of disease [17, 20]. In 2019, they affected a staggering 280 and 300 million individuals, respectively and these numbers still increased by 53 and 76 million during the Covid-19 pandemic [20, 21].

The massive harm caused by these diseases is further aggravated by the limitations of our therapies. Pharmacotherapy, psychotherapy and their combination can effectively help many patients suffering from anxiety and depressive disorders [22–24]. However, more than a fifth of patients with a depressive episode do not recover and go on to develop a chronic depressive illness [25]. Amongst individuals struggling with anxiety disorders, a third only reach intermittent recovery and almost one in ten suffers from consistent chronicity [26]. In the case of dementia, the situation is yet grimmer. Symptomatic therapies provide at most minimal alleviation [27] and novel antibody-based treatments produce only subtle effects and are severely limited in their applicability to real-world patients [28]. Therefore, prevention is even more urgent in our approaches to these diseases.

While the need for and preventive potential of improving social connection is becoming ever more evident, the best strategy to realise it is still debated. Major guidelines propose vastly different approaches. For example, the report by the US National Academies emphasizes targeting isolated individuals [6] while the report by the US Surgeon General underscores societal measures to improve social connection in the entire population [2]. In his seminal *The Strategy of Preventive Medicine*, Geoffrey Rose provided a theoretical framework for such arbitrations between different approaches. Therein, he distinguished two archetypal public health strategies. The high-risk strategy identifies patients at elevated risk and aims to specifically target their risk factor profiles by individualised interventions. The population strategy strives to move the risk factor distribution of the entire population to more favourable levels by systemic action. While the first strategy is the default clinical approach, the latter strategy takes two key epidemiological observations into account. Firstly, that the normal distributions of risk factors in populations living under different conditions are shifted against one another with disparate population means and secondly, that most cases of disease do not occur at the extreme risk factor levels of the greatest relative risk but at levels of merely moderately elevated risk that are far more prevalent [29, 30]. Therefore, tailoring preventive strategies to the higher risk factor levels may miss out a substantial share of those that could benefit.

**Fig. 1:**
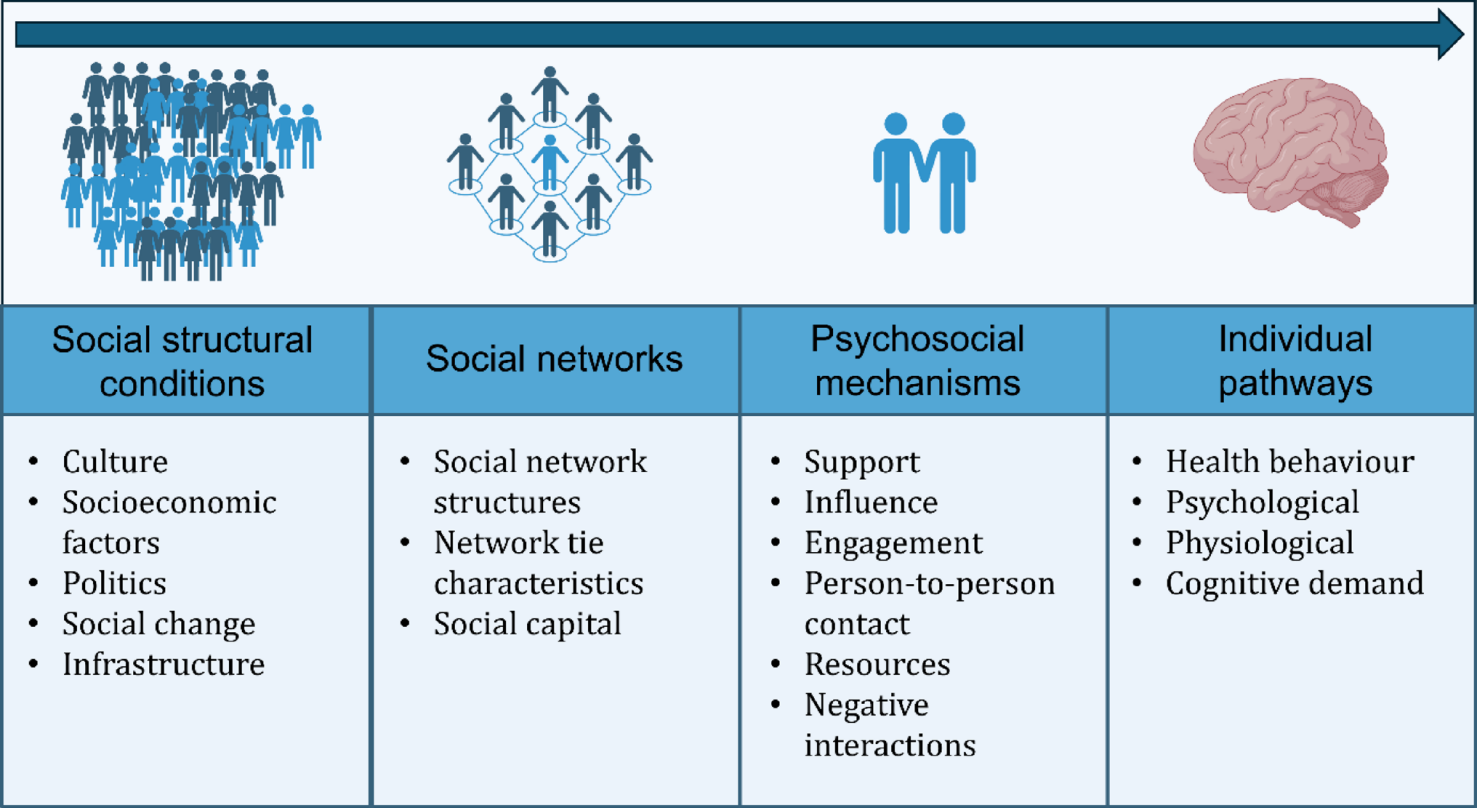
**A** A linear risk factor-outcome relationship would suggest the broad focus of a population strategy. **B** If an effect is observable at all risk factor levels but with a steeper slope beyond a threshold, this calls for a combination of approaches. **C** If effects are only observable beyond a threshold, preventive strategies should exclusively focus on those individuals adversely affected

If this is the case is determined by the shape of the relationship between social isolation and the outcomes. At one extreme, a relationship in which differences in social contact only affect the outcomes in socially isolated individuals would be strongly indicative of a high-risk strategy. This corresponds to the standard clinical practice of determining a cut-off value (e.g. less than twelve points on the six-item Lubben Social Network Scale) and focussing our attention on individuals that fall below this threshold [31]. At the other extreme, a linear relationship in which each reduction of social isolation by one unit would result in an equivalent outcome improvement would provide strong support for a population strategy. In between these cases, an exponential relationship would suggest a combination of a population strategy with particular attention to more isolated individuals (see Fig. 1) [30].

Hower, the actual shape has not really been investigated. Researchers either dichotomise participants as socially isolated or not isolated, or just model social isolation as a linear variable. Like many others, the authors of this paper have employed both approaches in the past but the decisions for either of the approaches have been largely based on convention rather than on evidence [32, 33]. The implications are substantial, though. If only those considered socially isolated suffer adverse effects, 100% of the total effect would be observed in this group. Yet, in the case of a Linear relationship, only 20% (i.e. the prevalence of social isolation) of the total effect would be suffered in this group. Given this paramount importance of the shape of the relationship for determining appropriate public health strategies, this is a pressing research gap to close.

Henceforth, we aimed to determine the shape of the relationship between social isolation and brain, cognitive and mental health using GAMMs to inform public health strategies against social isolation. To this end, we studied health data of a well-characterized population-based sample using generalized additive mixed models (GAMMs), a flexible and powerful statistical approach that is agnostic to the shape of multivariate relationships. We measured social isolation using the Lubben Social Network Scale (LSNS-6) and estimated brain health using hippocampal volume (HCV) and white matter hyperintensity volume (WMHV) derived from on advanced high-resolution MRI at 3 Tesla. Furthermore, we assessed cognitive functions based on parts A and B of the Trail Making Test and the full CERAD battery, and anxiety and depressive symptoms using the seven-item Generalized Anxiety Disease questionnaire (GAD7) and the 20-item Center for Epidemologic Studies Depression Scale (CESD). All measurements were gathered from a large well-characterized longitudinal adult sample (*n* > 10,000) from the Health Study of the Leipzig Research Centre for Civilization Diseases (LIFE) [34].

To this end, we regressed our outcomes of interest on a smooth (potentially nonlinear) term of LSNS-6 scores while controlling for relevant covariates in our *full* models and compared their fit to analogous *base* models defining the relationship with LSNS scores as linear. A non-linear fit would support a high-risk prevention strategy, while a linear fit would speak towards a broad prevention strategy.We have preregistered details on MRI preprocessing, measurement, a power analysis and statistical analyses at osf.io/6cz7t.

Therein, we specified three competing hypotheses on an equal footing: (A) The adverse effect of less social contact is linear across the entire spectrum of LSNS scores. (B) Less social contact has adverse effects across the entire spectrum of scores but the slope is steeper amongst scores considered to constitute social isolation. (C) Less social contact only has adverse effects if people are socially isolated whereas less social contact amongst more socially integrated individuals has no measurable effect on our outcomes. Figure 1 illustrates these hypotheses and the public health strategies they would be indicative of.

## Methods

### Study design, preregistration, code and reflexivity

We followed the standard guidelines for observational studies [35] and MRI studies [36, 37] in our reporting.

We preregistered our study at osf.io/6cz7tand published all code at github.com/LaurenzLammer/social_isolation_gamms. Please refer to the appendix for further information on the software. Furthermore, a reflexivity Sect. [38] and deviations from our preregistration such as changes in employed R functions for better visualisation aesthetics are provided in the appendix.

### Study population

We used all available data from the longitudinal ‘Health Study of the Leipzig Research Centre for Civilization Diseases’ (LIFE). The study was conducted according to the Declaration of Helsinki and approved by the institutional ethics board of the Medical Faculty of the University of Leipzig. It randomly selected and invited participants from the general adult population of Leipzig, a major city with 630,000 inhabitants in Germany. At baseline, over 10,000 volunteers participated of which a subgroup of around 2600 participants underwent MRI testing. Around 5,500 participated in the follow-up of which around 1,800 returned for in-person testing including MRI and cognitive testing. The baseline examinations were conducted from August 2011 to November 2014. Follow-up assessments were performed around 6–7 years after the respective first examinations [34]. The participants that did return for follow-up did not differ from those that did not in a substantial way at baseline. They were slightly older (Δ = 0.81 years), slightly fewer men (Δ = 0.12%), were a little more educated (Δ = 0.28 points (see below)) and had more social contact (Δ = 1.0 points LSNS). At baseline, the MRI subcohort was older (Δ = 2.83 years), contained more men (Δ = 7.97%), had slightly higher educational attainment (Δ = 0.23 points (see below)) and hardly differed regarding social contact (Δ = 0.21 points LSNS). 71% of the MRI subcohort participated in the follow-up while only 49% of the other participants returned.

### MRI data acquisition, processing, and quality control

We obtained T1-weighted and fluid-attenuated inversion-recovery (FLAIR) images on a 3 Tesla Siemens Verio MRI scanner (Siemens Healthcare, Erlangen, Germany) without major hardware modifications. Please refer to the appendix for details on the imaging protocol and quality control.

### Exclusion criteria

To keep our results as generalisable to the general population as possible, we only applied a minimal set of exclusion criteria. Thus, we only excluded those participants whose scans were deemed unusable in the quality control from analyses with MRI measures as dependent variable (*n* = 184, bl = 134, fu = 50). Additionally, we excluded those whose FLAIR scans had been classified as low quality due to either motion or lesions [39] from analyses with WMHV as dependent variable (*n* = 75, bl = 46, fu = 29).

## Measurements

### Social isolation

We used the standard 6-item Lubben Social Network Scale (LSNS) to measure the participants’ social isolation in which higher scores signify more social contact. The established cut-off deems participants with scores below 12 as socially isolated [31]. The questionnaire is a suitable tool to measure social isolation [40], has a high internal consistency (Cronbach’s α = 0.83), a stable factor structure of the family and non-kin subscale (rotated factor loading comparisons = 0.99) and good convergent validity (correlations with caregiver/emotional support availability and group activity all 0.2–0.46 across multiple sites) [31].

### Brain health outcomes

We processed the MRIs with FreeSurfer version 7.4.1 (RRID: SCR_001847) employing its longitudinal sequence-adaptive multimodal segmentation (SAMSEG) [41] to derive hippocampal, intracranial and white matter hyperintensity volume. Further details are provided in the appendix.

We averaged the hippocampal volume (HCV) over both hemispheres and adjusted it for intracranial volume according to the following formula:

HCV_adjusted, i_ = HCV_raw, i_ – β ∗ (ICV_raw, i_ − ICV_mean_).

where β is the unstandardized regression coefficient of HCV on intracranial volume (ICV) from a linear mixed-effects model (LME) and i denotes the observation [42]. ICV was added as a control variable to models with WMHV as a dependent variable (see below) [39].

### Cognitive functions

The entire sample performed the trail-making test parts A and B at baseline. A subcohort underwent more extensive cognitive testing at both timepoints using the extended German version of the Consortium to Establish a Registry for Alzheimer’s Disease (CERAD) test battery [43] additionally including semantic and phonematic fluency, learning, recall and recognition. We defined learning as the sum of three consecutive learning trials of the CERAD word list (10 words), recall as the sum of correctly recalled words after a delay, in which participants performed a nonverbal task, and recognition as the number of correctly recognized words out of a List of 20 presented afterwards. The tests were z-transformed for comparability.

We calculated domain-specific composite scores as follows [44]:

$$ \begin{aligned} Executuive\:functions = & (z(phonematic\:fluency)\: + \:z(semantic\:fluency)\: \\ & + \:z( - \frac{{TMT\:B\: - \:TMT\:A}}{{TMT\:A}}))/3 \\ \end{aligned} $$

$$Memory = \left( {\left( {z\left( {learning} \right) + z\left( {recall} \right) + z\left( {recognition} \right)} \right)} \right)/3$$

$$\:Processing\:speed=\:-\:z\left(TMT\:A\right)$$

Most participants were cognitively tested between 9 a.m. and 1 p.m.

### Depressive symptoms

We measured depressive symptoms as the sum score of the German version of the 20-item CESD which was developed to measure depressive symptoms in epidemiological studies. For each answer a score between 0 and 3 is assigned, resulting in a possible total score of 0 to 60. Higher scores imply more depressive symptoms. The scale has a high internal consistency (Cronbach’s α ~ 85%) and moderate to high external validity (measured as convergence with other measures of depressive symptoms) [45]. We aligned scores of inverted questions.

### Anxiety symptoms

To measure symptoms of anxiety we used the sum score of the 7-item General Anxiety Disorder scale (GAD7). Each item of the GAD7 is scored on a scale from 0 to 3 depending on symptom frequency leading to a total score range from 0 to 21 with higher scores indicating more anxiety symptoms. The questionnaire has a high internal consistency (Cronbach’s α = 0.92), test-retest reliability (intraclass correlation = 0.83) and agreement with clinical diagnosis (89% sensitivity and 82% specificity at a cut-off of 10 points). The scale is well suited to screen for generalized anxiety disorder [46] but also detects anxiety symptoms more broadly (including symptoms of social anxiety and panic disorder) [47]. The German version of the scale showed a similarly high internal consistency (Cronbach’s α = 0.89) and validity [48].

### Gender

Participants had to choose their gender in a binary female/male manner. Note that the German ‘Geschlecht’ does not differentiate between sex and gender. The lack of clarification and other options is lamented by the authors.

### Education

The participants’ education was assessed using an extensive questionnaire, scored on a range from 1 (no degree at all) to 7 (A-levels + master’s degree [or equivalent] or promotion) [49] and dichotomously categorized with a cut-off at a score < 3.6 based on prior research on education as a protective factor against dementia [50]. Baseline education levels were used for all timepoints.

### Income

We calculated an equivalised income according to the OECD-modified scale:

$$\:equivalised\:income=\:\left(net\:household\:income\right)\:/$$ $$\:(1+\:0.5*(\text{a}\text{d}\text{d}\text{i}\text{t}\text{i}\text{o}\text{n}\text{a}\text{l}\:\text{h}\text{o}\text{u}\text{s}\text{e}\text{h}\text{o}\text{l}\text{d}\:\text{m}\text{e}\text{m}\text{b}\text{e}\text{r}\text{s}\:\ge\:\:15\:\text{y}\text{e}\text{a}\text{r}\text{s})\:\:$$ $$\:+\:0.3*(\text{a}\text{d}\text{d}\text{i}\text{t}\text{i}\text{o}\text{n}\text{a}\text{l}\:\text{h}\text{o}\text{u}\text{s}\text{e}\text{h}\text{o}\text{l}\text{d}\:\text{m}\text{e}\text{m}\text{b}\text{e}\text{r}\text{s}\:<\:15\:\text{y}\text{e}\text{a}\text{r}\text{s}))\:\:$$

We scored the equivalised income on a scale ranging from 1.0 points (< 800€) to 7.0 points (≥ 3000€) following the established adaptation of the suggestions of the Robert Koch-Institute (RKI) [49].

### Socioeconomic status

We measured socioeconomic status (SES) as a metric variable according to the guidelines developed at the RKI [49]. In addition to education and income measured as described above, occupation contributed to SES as a third factor. In addition to linear SES scores used as covariates, we categorized the resulting SES scores according to the general population quintile ranges provided by Lampert et al. [49] for weighted analyses (see below). Baseline SES scores were used for all observations.

### Hypertension

To control for hypertension, we used a dichotomized variable. We considered participants.

To have hypertension if they had a previous diagnosis of hypertension, took antihypertensive medication (see code for exact list of ATC codes), had an average systolic blood pressure over 140 mmHg or average diastolic blood pressure higher than 90 mmHg during the examination. Blood pressure was measured three times. The first measurement was performed after 5 min of rest and three additional minutes of rest passed between each of the following measurements.

We treated biologically implausible systolic blood pressures > 200 and < 80mmHg as NAs. The threshold was exceeded in 108 observations of which 88 were above 390mmHg. Only two observations were below the threshold.

### Diabetes

As to control for hypertension, we used a dichotomized diabetes variable. We deemed participants to have diabetes if they had a previous diagnosis of diabetes, took antidiabetic medication (with the exception of SGLT-2 inhibitors because of their broader set of indications, see code for exact list of ATC codes), or their HbA1C measured by turbidimetry was ≥ 6%.

### BMI

We calculated the body-mass-index (BMI) according to the standard formula:

$$\:BMI=\text{w}\text{e}\text{i}\text{g}\text{h}\text{t}\:\left[\text{k}\text{g}\right]/{\text{h}\text{e}\text{i}\text{g}\text{h}\text{t}\:\left[\text{m}\right]}^{2}$$

Biologically implausible BMIs > 50 or < 15 were treated as NAs due to probable measurement or documentation error. 13 observations were below threshold (all < 11) and 26 above the threshold of which ten were three-digit values.

### Age

We determined the participants’ age based on the date of the LSNS. If no LSNS data were available for a timepoint the date of the MRI, CESD and GAD7 were used in this order.

### Living alone

We created a dichotomous variable differentiating participants living alone from those living with others.

### Occupation

We classified participants as non-working if they declared not to be gainfully employed due to other reasons than studying, military, or alternative service.

### Marital status

We only considered participants to be married if they also lived with their spouses to avoid including separated but not yet divorced persons as this is more appropriate for the topic at hand.

### Ventricular enlargement

We created a dichotomous variable indicating if the quality control showed that ventricular enlargement absorbed white matter hyperintensities from baseline to follow-up.

### Outliers

GAMMs are better capable to deal with outliers than normal linear mixed models as confidence intervals will just grow larger around rarer values [51]. Thus, we did not exclude outliers from our analyses.

### Imputation

We used multiple imputation by chained equations to handle missing data [52] to produce five imputed datasets. Please refer to the appendix for details.

### Transformations

To approximate a normal distribution of CESD scores when used as a covariate, we performed an asinh transformation.

### Statistical models

We investigated the shape of the relationship of LSNS scores with adjusted HCV, WMHV, executive functions, memory, processing speed, anxiety symptoms and depressive symptoms using GAMMs.

Generalized additive mixed models (GAMMs) present the optimal method to answer this question. They extend generalized linear mixed models by allowing for smooth terms. Smooth terms facilitate an approach to modelling the relationship between variables that is agnostic to the shape at the outset. As they are based on multiple base functions, they can flexibly model various nonlinear shapes that are more faithful to the observed data than predetermined linear, quadratic, etc. shapes. At this, they avoid overfitting by incorporating a cost function that minimises both residuals and the complexity of the smooth term. Accordingly, the resulting shape can be linear if the reduction of residuals does not outweigh the added complexity or take polymorphic shapes in cases of sufficient improvements in faithfulness to the data [51, 53].

For each of our seven outcomes, we calculated four models differing in the number of covariates and the shape constraints of the LSNS predictor. In model 1, we only controlled for gender and the nonlinear effect of age. In model 2, we additionally controlled for further covariates. For MRI measures and cognitive functions, we added education, BMI, diabetes, hypertension and asinh-transformed depressive symptoms. In model 2 for WMHV, we furthermore included ICV and ventricular enlargement as covariates. For mental health outcomes, we added SES, BMI, diabetes and hypertension. Fig S6 illustrates the assumptions underlying models 1 and 2. In the *full* models, we predicted the outcomes by a smooth LSNS term whereas in the *base* models, we defined LSNS as a linear predictor. In the gamm4 syntax, smooth terms are demanded by s(*predictor*) in the model formula. The smooth terms allow us to investigate whether there is sufficient evidence in the data to justify the increased complexity of a nonlinear relationship. The *base* models provide a benchmark to test if the more complex *full* models outperform them. In each model, we calculated random intercepts for each participant by adding the argument “random = ~(1|ID)”.

Combing the different sets of covariates and *base* and *full* models results in the following four models written in the gamm4 syntax per outcome:

$${\text{Full model 1}}:\,dv=gender~+~s\left( {age} \right)~+~s\left( {LSNS} \right)$$

$${\text{Base model 1}}:\,dv=gender~+~s\left( {age} \right)~+~LSNS$$

$${\text{Full model 2}}:\,dv=gender~+~s\left( {age} \right)~+~s\left( {LSNS} \right)~+~further~covariates$$

$${\text{Base model 2}}:\,dv=gender~+~s\left( {age} \right)~+~LSNS~+~further~covariates$$

HCV and executive functions could be modelled as AMMs with normal error distributions and identity links, but due to their non-normal distribution we had to calculate GAMMs for GAD7, CESD, WMHV, memory and processing speed. For GAD7 and CESD we used a Poisson distribution with a log link. We employed a gamma distribution for WMHV, memory and processing speed. To obtain a right-skewed distribution without values = < 0, we negated the scores of memory and processing speed and Then subtracted their minimum value and added 0.00001. For WMHV and memory we had to use a log link. For processing speed, an identity link produced a diagnostically sound fit.

### Weighting

As participants in lower SES quintiles are underrepresented in the LiFE-Adult study, we used weights to counterbalance this in our analyses. We calculated the weights relative to the category prevalence in our sample and the 20% prevalence in the general population:

$$\:weight=20\%/quintile\:prevalence\:in\:sample\:in\:\%$$

All participants with no available SES scores before imputation were assigned a weight of 1.

### Model diagnostics

We used the gam.check function to test for oversmoothing and to produce diagnostic plots (q-q plot, residuals plotted against linear predictor, histogram of residuals, response plotted against fitted values) of our models. We detected oversmoothing in our processing speed models and hence increased the dimensions of the basis used to represent the smooth term to 20 for the age and LSNS terms. All diagnostic plots showed satisfying results. Furthermore, we checked for concurvity, the nonparametric extension of the concept of multicollinearity with the concurvity function. The worst-case scenario concurvity did not exceed our prespecified threshold [54] of 0.8 for any of our smooth terms (the maximum was 0.305).

Moreover, we tested for multicollinearity amongst our parametric terms using variance-inflation factors (VIFs) derived using the car package’s vif() function on Linear models of the Linear predictors for each outcome. The VIFs did not exceed our preregistered cut-off of 10 with the maximum across models and imputations reaching 1.68.

### Model comparisons

To investigate whether the *full* model with a smooth LSNS term outperforms the *base* model with a linear LSNS term, we compared them with the anova function. We always compared the *full* and the *base* models of the same outcomes with the same set of covariates. If the returned p-values are low, this would suggest a significant difference between the models and thus a significant added value of the greater complexity. Furthermore, the returned BICs assist in comparing the two models.

### Population-level absolute effect share calculation

To estimate how much of the effect of social isolation is amongst those deemed socially isolated (LSNS < 12), we calculated local gradients for each LSNS score. We extracted estimates for each LSNS score from the models using the smooth_estimates() function of the gratia package and calculated the gradients based on the difference to the estimates of the adjacent scores. The resulting gradients were then proportioned to the relative frequency of the LSNS scores before comparing the shares amongst scores below and above the cut-off.

### Inference criteria

To test the significance of LSNS as a predictor of our outcomes, we used one-sided approximate p-values of the smooth term for LSNS returned by gamm4() and the standard α-level of 0.05.

Drawing conclusions on the shape of the relationship between our predictor of interest and our outcomes is not straightforward. A qualitative assessment of the plotted partial effect of LSNS was our primary step to evaluate our models with regards to our prespecified hypotheses. If a log link-function had been used, we transformed the estimates to the original scale. Then, this was supplemented by the quantitative measures of the anova comparison of the *full* and *base* models. Namely, the p-value and the BIC values of both models.

A significant p-value at the α-level of 0.05 combined with a lower BIC for the *full* model would be a clear indication of a non-linear relationship. If this goes in hand with a steeper slope at lower LSNS scores, this would be in support of hypothesis B or C depending on the shape.

### Families of tests

We consider HCV, WMHV, and the three cognitive functions as facets of cognitive aging to form a family of tests. Accordingly, we FDR-corrected the s(LSNS) p-values for these outcomes using the qvalue() function. Models 1 and 2 were FDR-corrected separately.

p-values from model comparisons were not FDR-corrected.

### Sensitivity analyses

To test the robustness of our results, we conducted sensitivity analyses. We calculated the models without SES weights and without imputed data. Furthermore, we modelled two separate smooths for men and women to explore this potential moderating factor. We did this by adding the argument by = gender to the LSNS smooth terms. Analogously, we conducted further exploratory stratified analyses (completed 1/2 timepoints, above/below 65 years (German retirement age), above/below median SES, married/unmarried, living alone/cohabitating).

### Power analysis

We detected modelled nonlinearity in 91.75% of cases resulting in a β well below the standard of < 20%, indicating adequate power to detect effects with our sample size. In 19.29% of purely linear simulations, the anova indicated nonlinearity. However, in these cases, the actual shape could be easily deduced from the accompanying plots of partial effects. Please refer to the appendix for further methodological information.

## Results

We included all participants of the LIFE-Adult study [34] To keep our sample as representative as possible, we refrained from restrictive exclusion criteria. We only excluded participants with problematic MRI scan quality from models with MRI measures as outcomes. In total, we analysed 10,411 participants at baseline with sample sizes of ~ 10,300 for processing speed, GAD7 and CESD, ~ 3480 for memory and executive functions and ~ 2600 for MRI measures. At follow-up, we analysed data from 5,664 with sample sizes of ~ 5,640 for GAD7 and CESD, ~ 1780 for cognitive functions and ~ 1600 for MRI measures. The mean age at baseline and follow-up was 58 and 64 years, respectively. 53% of participants were female. The sample displayed a high prevalence of cardiovascular risk factors, with ~ 60% presenting with hypertension and 13–14% with diabetes. Less than 10% had no form of tertiary education. Individuals exhibited LSNS scores ranging across the whole spectrum, with an average score of 16–17 similar to other populations [31]. Tables 1 and 2 show descriptive data for observed data and of the 1 st imputed dataset, respectively.

**Table 1:**
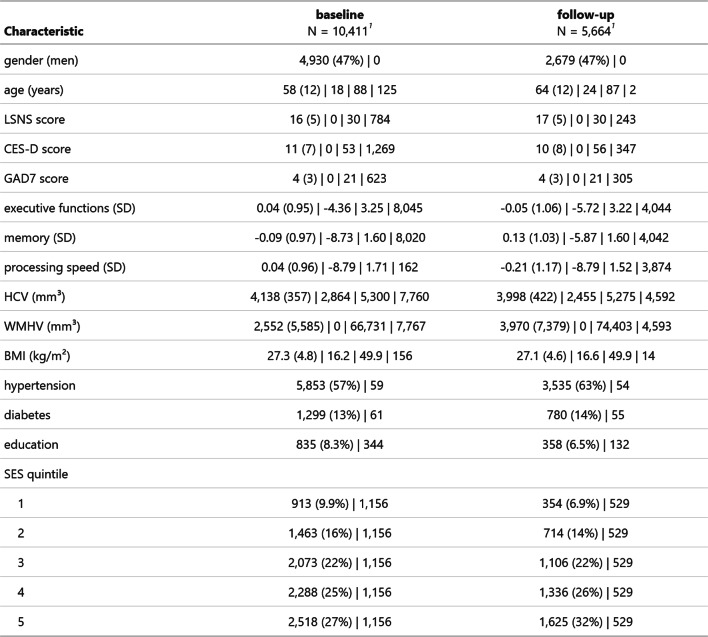
Descriptive statistics of the sample

^1^categorical: n(%) yes | n missing; continuous: Mean (SD) | minimum | maximum | n missing

**Table 2:**
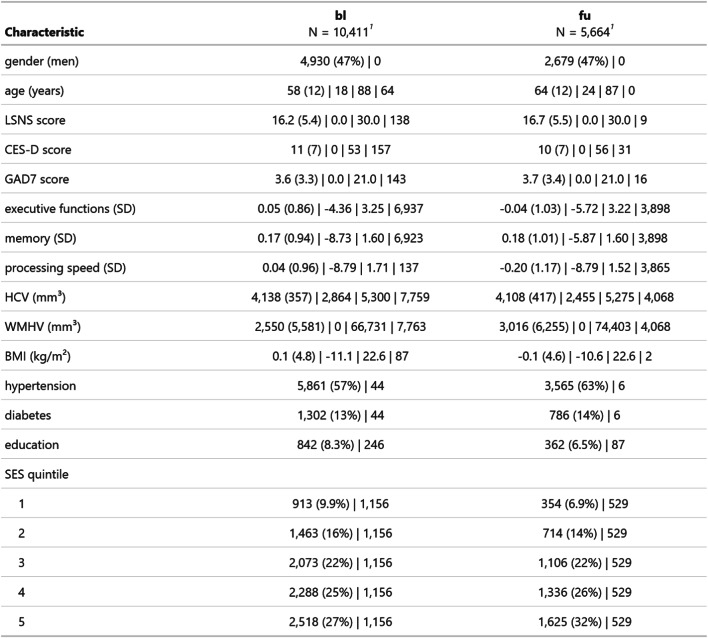
Descriptive statistics of the sample of the first imputation

^1^categorical: n(%) yes | n missing; continuous: Mean (SD) | minimum | maximum | n missing

### The effect of social isolation

The direction of effect showed that more social contact is related to better outcomes for all dependent variables across models. This also holds for the multiple imputed datasets accounting for the greater uncertainty of imputed variables. All outcomes showed consistent and clear levels of significance often below the floating-point precision of our machines (~ 2.2 * 10^−16^), except for HCV and WMHV. For HCV, the α-level was exceeded in a single imputation in model 2 when correcting for multiple comparisons (FDR-corrected q-values in model 1: 0.0012–0.0203 (5/5 significant); model 2: 0.0053–0.0575 (4/5 significant)). The FDR-corrected q-values for the effect of LSNS on WMHV did only reach significance in a few imputations, though (FDR-corrected q-values in model 1: 0.0496–0.3976 (1/5 significant); model 2: 0.0318–0.344(2/5 significant)). Table 3 provides an overview of the measures of significance for the different outcomes across imputations.

**Table 3:**
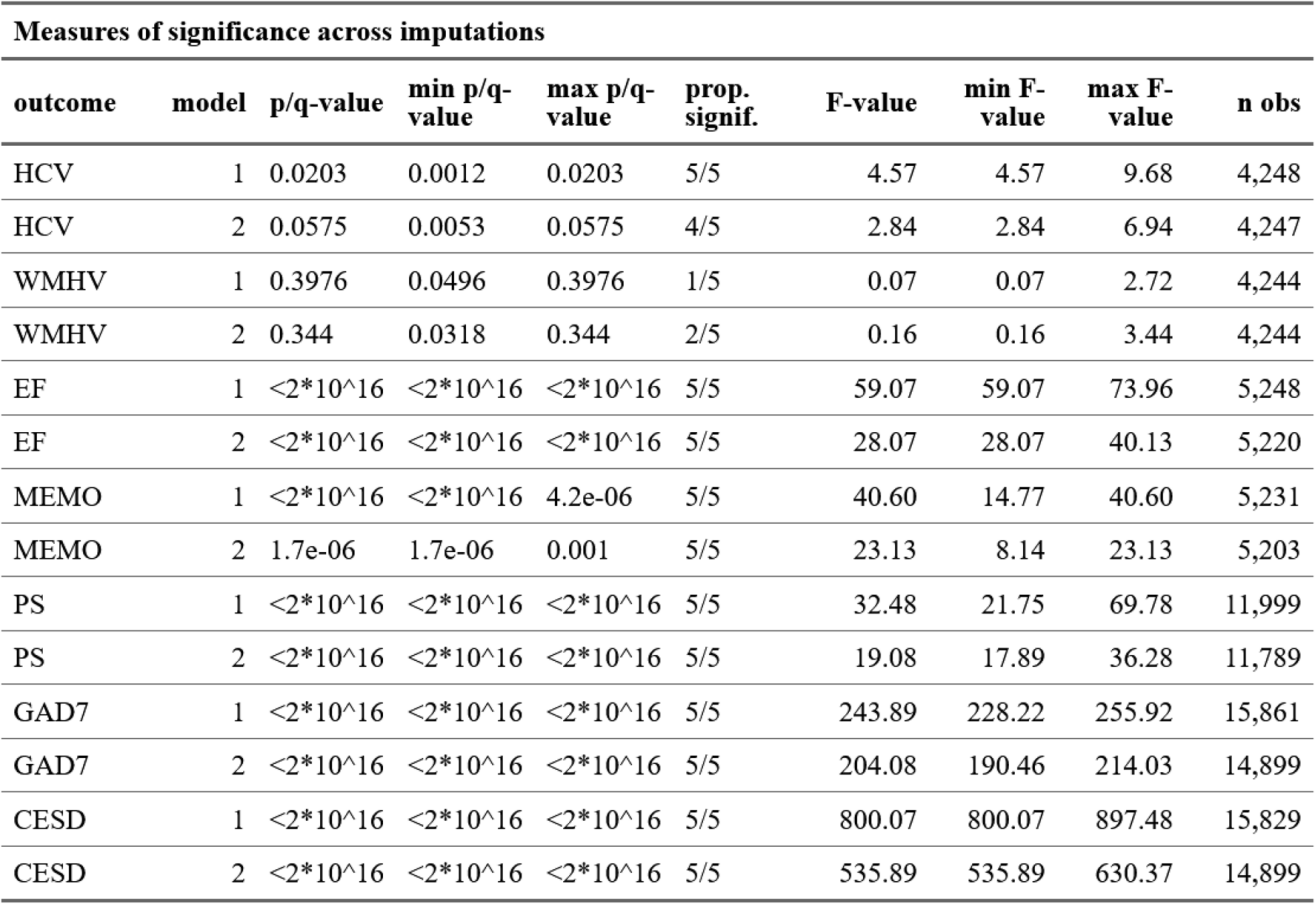
Measures of significance across imputations

*HCV* hippocampal volume, *WMHV* white matter hyperintensity volume, *EF* executive functions, *MEMO* memory, *PS* processing speed, *GAD7* 7-item generlaized anxiety disease questionnaire, *CESD* 20-item center for epidemiologic studies depression scale

**Fig. 2:**
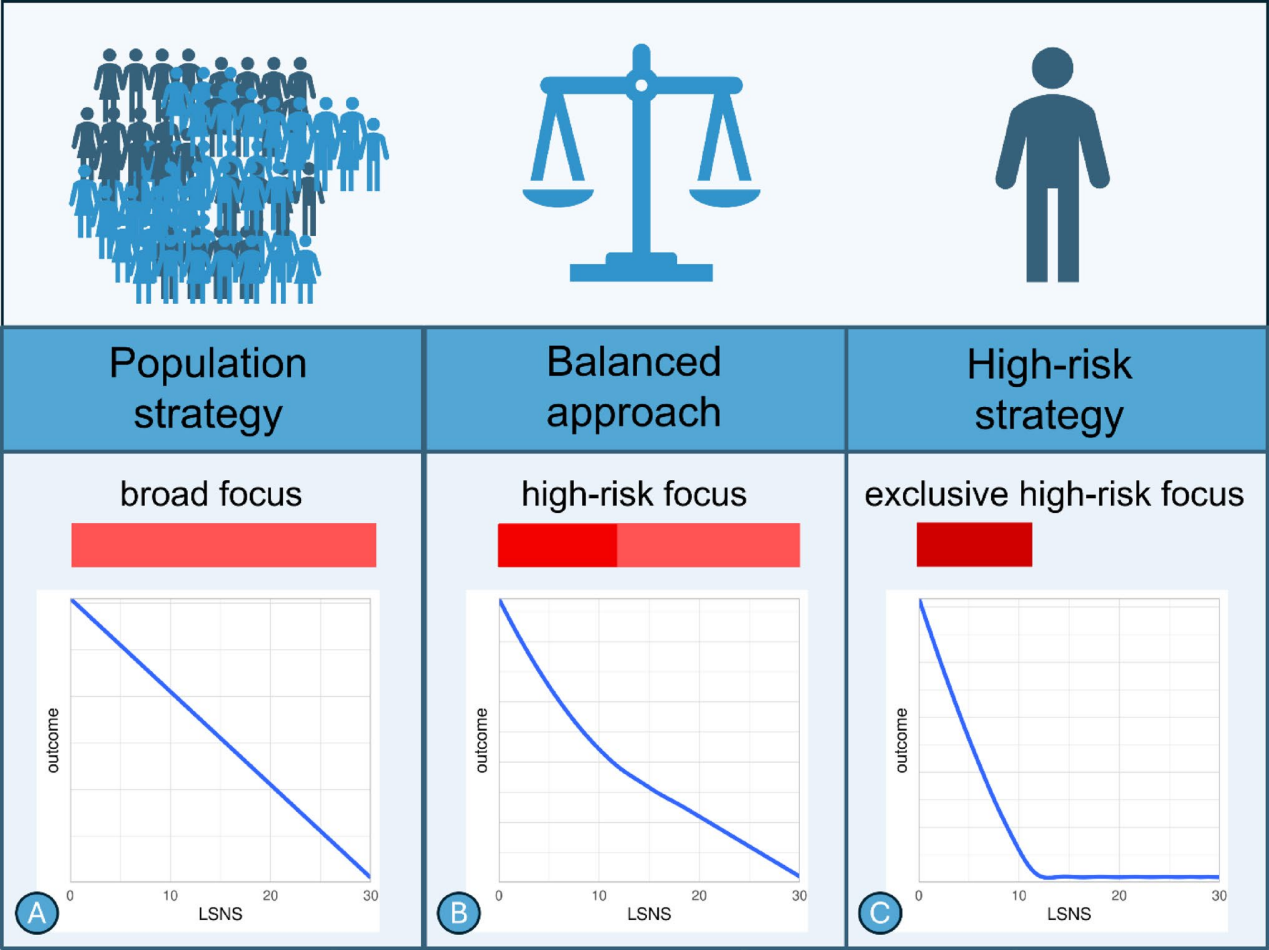
Results of model 1. **A** The partial effect of age on hippocampal volume (HCV in the 1 st imputed dataset). **B** The partial effect of Lubben Social Network Scale (LSNS) scores on hippocampal volume (HCV) in the 1 st imputed dataset. The dashed vertical line illustrates the standard LSNS cut-off. The density plot at the bottom depicts the distribution of LSNS scores with those considered socially isolated shown in red. **C** Partial effects of LSNS scores on HCV across the five imputations. The waffle plot indicated that the FDR-corrected q-values were significant in all five imputations. **D** The partial effect of age on white matter hyperintensity volume (LESIONS) in the 1 st imputed dataset. **E** The partial effect of Lubben Social Network Scale (LSNS) scores on white matter hyperintensity volume in the 1 st imputed dataset. The dashed vertical line illustrates the standard LSNS cut-off. The density plot at the bottom depicts the distribution of LSNS scores with those considered socially isolated shown in red. **F** Partial effects of LSNS scores on white matter hyperintensity volume across the five imputations. The waffle plot indicated that the FDR-corrected q-values were significant in one of five imputations. * < 0.05; ** < 0.01; *** < 0.001. HCV and WMHV are measured in mm3. Grey areas indicate 95% confidence intervals

**Fig. 3:**
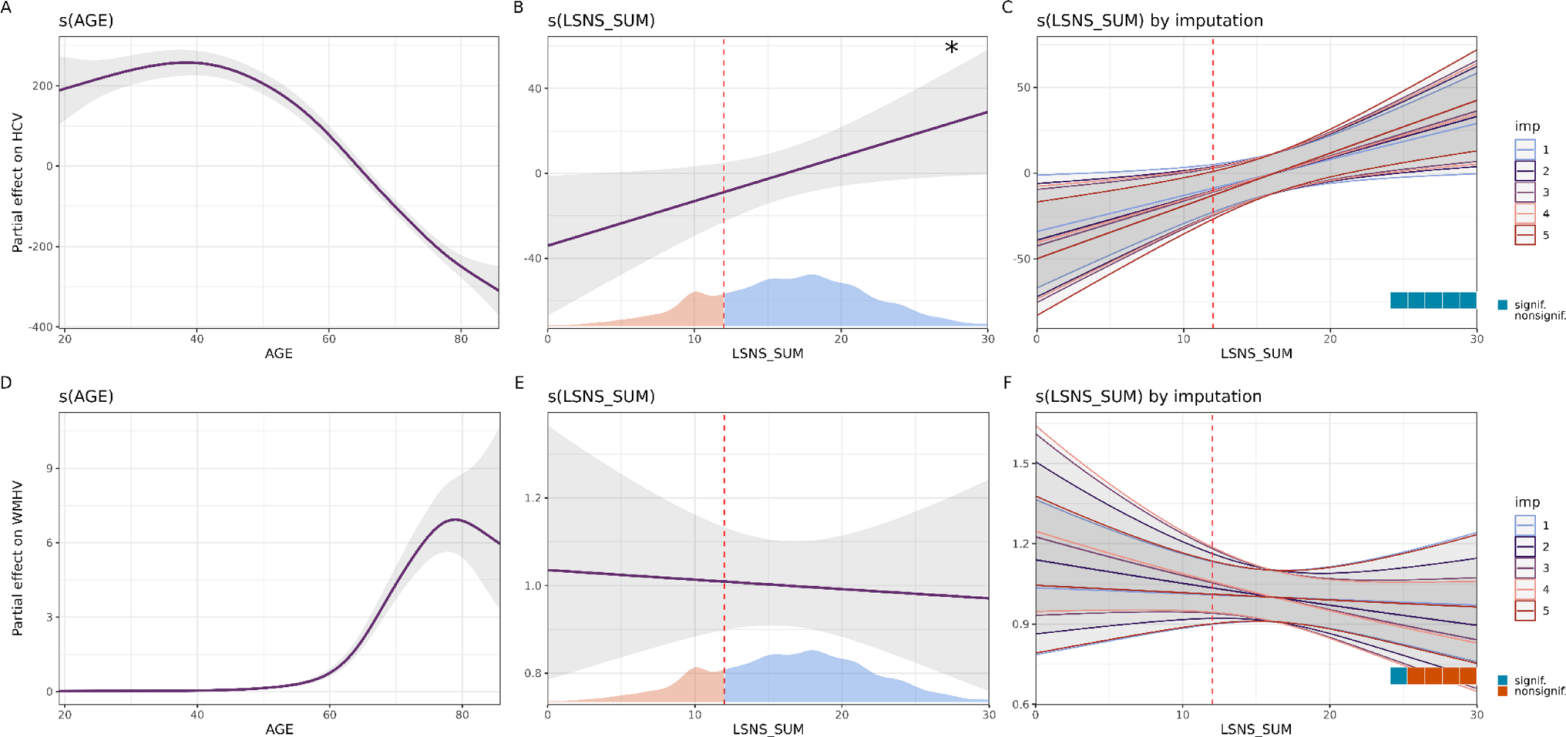
Results of model 1. **A** The partial effect of age on executive functions in the 1 st imputed dataset. **B** The partial effect of Lubben Social Network Scale (LSNS) scores on executive functions in the 1 st imputed dataset. The dashed vertical line illustrates the standard LSNS cut-off. The density plot at the bottom depicts the distribution of LSNS scores with those considered socially isolated shown in red. **C** Partial effects of LSNS scores on executive functions across the five imputations. The waffle plot indicated that the FDR-corrected q-values were significant in all five imputations. **D** The partial effect of age on memory in the 1 st imputed dataset. **E** The partial effect of Lubben Social Network Scale (LSNS) scores on memory in the 1 st imputed dataset. The dashed vertical line illustrates the standard LSNS cut-off. The density plot at the bottom depicts the distribution of LSNS scores with those considered socially isolated shown in red. **F** Partial effects of LSNS scores on memory across the five imputations. The waffle plot indicated that the FDR-corrected q-values were significant in all five imputations. **G** The partial effect of age on processing speed in the 1 st imputed dataset. **H** The partial effect of Lubben Social Network Scale (LSNS) scores on processing speed in the 1 st imputed dataset. The dashed vertical line illustrates the standard LSNS cut-off. The density plot at the bottom depicts the distribution of LSNS scores with those considered socially isolated shown in red. I) Partial effects of LSNS scores on processing speed across the five imputations. The waffle plot indicated that the FDR-corrected q-values were significant in all five imputations. * < 0.05; ** < 0.01; *** < 0.001. Cognitive functions are measured in standard deviations. Grey areas indicate 95% confidence intervals

**Fig. 4:**
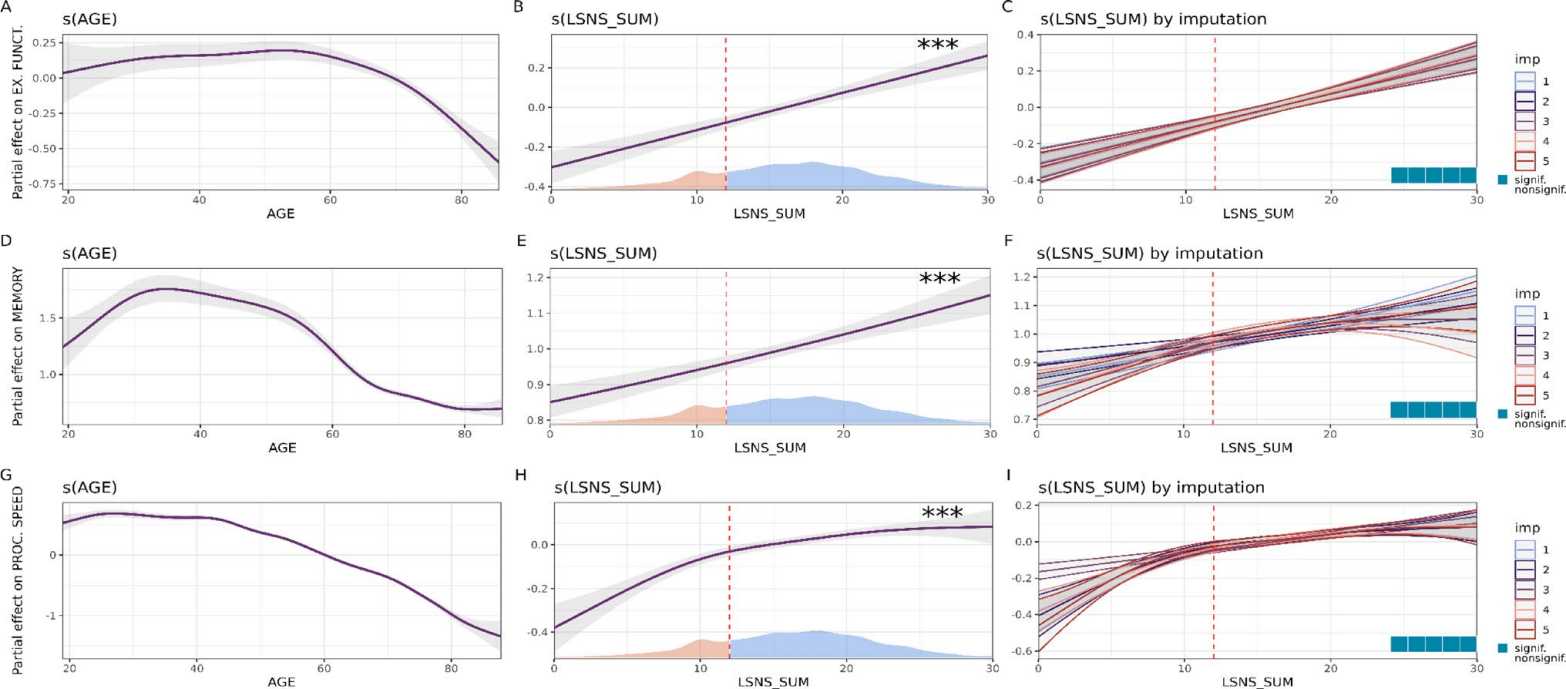
Results of model 1. **A** The partial effect of age on anxiety symptoms in the 1 st imputed dataset. **B** The partial effect of Lubben Social Network Scale (LSNS) scores on anxiety symptoms in the 1 st imputed dataset. The dashed vertical line illustrates the standard LSNS cut-off. The density plot at the bottom depicts the distribution of LSNS scores with those considered socially isolated shown in red. **C** Partial effects of LSNS scores on anxiety symptoms across the five imputations. The waffle plot indicated that the FDR-corrected q-values were significant in all five imputations. **D** The partial effect of age on depressive symptoms in the 1 st imputed dataset. **E** The partial effect of Lubben Social Network Scale (LSNS) scores on depressive symptoms in the 1 st imputed dataset. The dashed vertical line illustrates the standard LSNS cut-off. The density plot at the bottom depicts the distribution of LSNS scores with those considered socially isolated shown in red. **F** Partial effects of LSNS scores on depressive symptoms across the five imputations. The waffle plot indicated that the FDR-corrected q-values were significant in all five imputations. * < 0.05; ** < 0.01; *** < 0.001. GAD7 and CESD are measured in points on the respective questionnaire. Grey areas indicate 95% confidence intervals

Due to the potential nonlinearity, GAMMs do not provide effect sizes in terms of β as regular linear models do. Therefore, we plotted the partial effects of LSNS next to that of age in Figs. 2, 3 and 4 to allow our readers to compare the effect size across the spectrum of LSNS scores with that across our age range.

### The shape of the effect of social isolation

Our models determined a Linear shape to strike the optimal balance between parsimony and faithfulness to the data for almost all LSNS-outcome relationships. In the case of depressive symptoms, the slope showed some wiggliness but generally followed a Linear form. Only in the case of processing speed, we observed a shape resembling an exponential relationship with a steeper slope for scores below 10. Figures 2, 3 and 4 depict the partial effects of LSNS on our outcomes and the shape variability across imputations for models with limited covariates. Analogous figures for models with all covariates are presented in figs S7-9.

Quantitative measures did not show that the more complex *full* models outperformed the simpler *base* models (all p-values > 0.05, all ΔBIC < −6) except for processing speed where both p-value and BIC prefer the *full* model when controlling for all covariates (*p* < 1 * 10^−3^, ΔBIC = 12.8). Table 4 provides an overview of the results of the anova comparisons of the *full* and *base* models for all outcomes.

**Table 4:**
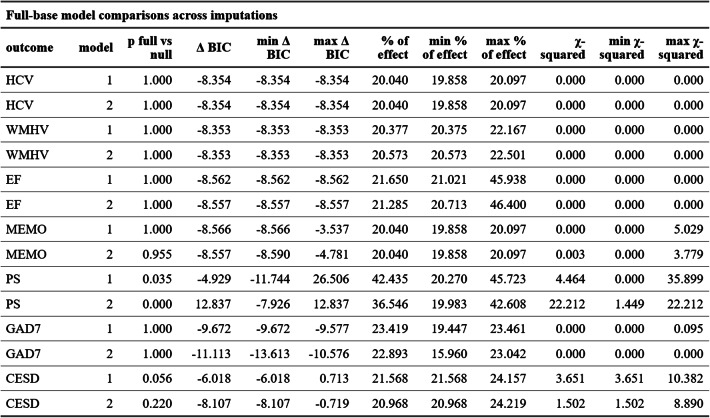
Full versus base model comparisons across imputations

*HCV* hippocampal volume, *WMHV *white matter hyperintensity volume, *EF *executive functions, *MEMO* memory, *PS* processing speed, *GAD7* 7-item generlaized anxiety disease questionnaire, *CESD* 20-item center for epidemiologic studies depression scale

### The distribution of the effect

LSNS scores vary across the entire range of 0 to 30 points and approximate a normal distribution. Around 20% are below the standard threshold of 12 points. Therefore, the proportion of the Linear effect of LSNS on the outcomes WMHV, HCV, executive functions, memory, anxiety and depressive symptoms amongst those not considered to be socially isolated is around 80%. Regarding processing speed, socially isolated individuals bear a larger share of the effect but, considering the gradient at each and distribution of LSNS scores, the share is still predominantly amongst those not considered to be socially isolated (model 1: 57.6%; model 2: 64.5%). Accordingly, the results for processing speed are aligned with hypothesis B while all other outcomes are in line with hypothesis A. Please see Table 4 for the proportion of the effect amongst socially isolated participants for all models.

### Sensitivity analyses

Neither calculating unweighted models nor only using observed data changed the results substantially or with regards to our hypotheses. Models without SES weights showed slightly smaller effect sizes. When analysing only observed data, all models were significant including those with WMHV as dependent variable. Figs S10-21 show their results analogously to Figs. 2, 3 and 4 and S7-9. Quantitative measures are presented in Tables S1-4. Separate LSNS-smooths by gender did not reveal consistent differences in the effects of social isolation in men and women. The nonlinearity in processing speed seemed to be stronger in women and the effect size on HCV to be greater in men, though. There were also more pronounced effects on processing speed amongst those Living alone, the unmarried and participants with higher SES. Participants that only completed one of the two timepoints showed larger effect sizes in models with cognitive functions and MRI measures as dependent variables. Furthermore, individuals with a lower SES showed slightly stronger effects of LSNS on depressive symptoms. The effect on executive functions amongst those Living alone and those above 65 years tended towards an inverse U-shape. The breadth of the confidence interval of these stratified partial effects makes them compatible with multiple shapes in the higher LSNS score range, though. Figs S22-39 depict the stratified partial effects of LSNS on our outcomes.

## Discussion

In this pre-registered study, we pursued a theoretically- informed and data-driven approach to discern appropriate preventive public health strategies against social isolation. Based on data of a well-characterized large longitudinal sample, we found social isolation to significantly predict brain, cognitive and mental health outcomes consistently except for WHMV. For all outcomes other than processing speed, the relationship with LSNS scores was effectively linear and thus in line with hypothesis A. Processing speed was the only outcome in line with hypothesis B that suggested that more social contact would be disproportionally beneficial for socially isolated individuals. Not a single of our models suggested that more social contact would only be beneficial for individuals that would be considered socially isolated (hypothesis C). For all outcomes, most of the absolute harm could be observed amongst individuals that would not be categorised as socially isolated. These findings proved to be robust and independent of gender moderation in multiple sensitivity analyses.

Moreover, our findings question the grim picture painted by focussing our attention on social isolation. More social contact was related to better outcomes across LSNS scores without evidence of saturation. Actually, all major theories on the mechanism of action (stress-buffering hypothesis, main-effect hypothesis, cognitive demand hypothesis) explain social isolation’s link to health outcomes by means of the benefits of social contact rather than by the harms of its absence [7, 9, 55, 56]. Thus, we could embrace the salubrious effects of more social contact in addition to being concerned with the adverse effects of isolation [2].

## Implications for public health

Prima facie, the effect size of social isolation on a single outcome is not massive. Its high prevalence and adverse effects on multitudinous health outcomes makes it a pressing public health concern, nonetheless. This is underscored by our results. Moreover, the positive effects of social contact on quality of life [57, 58], that have also been argued to be linear [59], add further importance to this issue. Still, preventive public health strategies should not exclusively focus on social isolation but target a variety of modifiable risk factors to maximise their effectiveness [10, 60].

A few assumptions and approximations were necessary to enable our statistical analyses. Hence, interpretation must proceed cautiously. Linearity in our models cannot be equated with true Linearity of effects. Still, the general direction of the results is clear enough to suggest that the current focus on individual-level interventions against social isolation is Likely to be suboptimal at least for the prevention of dementia, cognitive decline, anxiety and depression. Au contraire, they point to population-level interventions that aim to foster social connection across society as more promising approaches to prevention. Only in efforts to sustain processing speed, a focus on socially isolated individuals in addition to a population strategy would be indicated by our models. Individuals scoring above the LSNS cut-off would not attract attention in standard clinical practice. Yet, our findings suggest that they experience 60–80% of the health effects of social contact. This helps to explain the poor performance of dementia risk prediction models that try to identify high-risk individuals [61]. Accordingly, our results are in line with and provide empirical evidence for recent calls for shifting our efforts to population-level preventive interventions [62, 63]. Such interventions hold promise but it must be acknowledged that a share of the observed effect sizes is probably attributable to reverse causation. Hence, we must adjust our expectations concerning the preventive potential of such interventions accordingly.

**Fig. 5:**
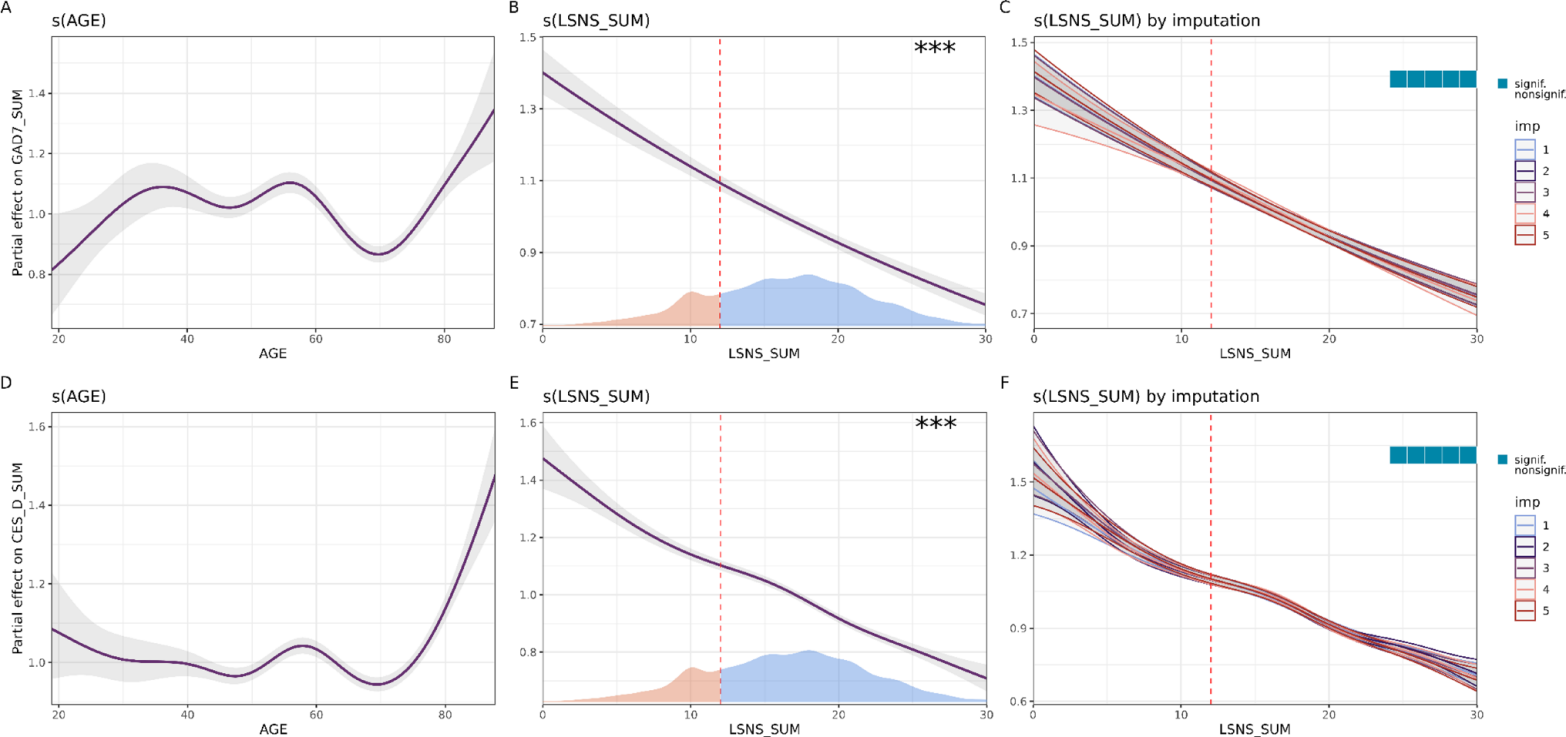
Determinants of social connection from societal characteristics and policies down to the individual level Adapted from Berkman & Krishna [64]

Still, the shape of the relationship is not the only consideration Rose suggested for arbitrating between public health strategies. The sheer prevalence of social isolation, affecting around 25% of the elderly population [4], is suggestive of a population strategy, too. Population strategies innately scale more easily to large numbers of beneficiaries and avoid screening costs whose value diminishes at increasing base rates. Furthermore, being labelled as socially isolated can be stigmatising [6] and a population strategy would evade this concern. Moreover, prevention harms, a common reason to pursue high-risk strategies, are not of concern in the case of social isolation. However, one must acknowledge that our medical systems are geared towards and much more experienced in providing high-risk strategies. While interventions could take place on all levels (see Fig. 5) [64–66], almost all existing methods to reduce social isolation like social prescribing are interventions on the (inter)individual level [67–70].

## Limitations

This work takes the notion that social isolation should be intervened upon as its starting point. It tries to illuminate how this might be done rather than if this should be done. We believe this to be needed given the numerous campaigns by governments around the globe aiming to improve social connection. However, it must be noted that reverse causation is a plausible explanation for the observed associations between social isolation and our outcomes, too. The correlative nature of this work cannot answer this question. Research focusing on directionality points towards a bidirectional connection with mental health outcomes [71] while the evidence on cognitive outcomes varies [72, 73]. Factoring this bidirectionality in means that a share of the strength of any correlation must be attributed to reverse causation. Thus, the actual preventive potential of reducing social isolation is likely to be smaller than effect sizes might initially suggest. Concerning cognitive functions, the relative importance of reverse causation is likely to surge as cognitive decline progresses. Careful analysis of longitudinal data with numerous timepoints would be necessary to elucidate when cognitive impairment leads to social withdrawal.

Another concern is social isolation’s level of measurement. In our work the LSNS is treated as a scale with proportionate intervals. Its scores are not linearly translatable into quantities like time spent with friends in hours, though. It is thus arguably an ordinal scale [74]. The same concerns apply to our questionnaire-based outcomes depressive symptoms and anxiety symptoms. Three lines of argumentation can justify our treatment of LSNS scores, nonetheless. Borgatta and Bohrnstedt argued that although the measurement scales employed in the social sciences are hardly ever true interval or ratio scales, the constructs we try to measure are usually continuous and that our scales measure them with measurement error [75]. This is supported by the LSNS scores being normally distributed. Additionally, multiple statisticians have argued to treat measurements on ordinal scales as if they had proportionate intervals for purely pragmatic reasons and to accept that approximations are made in doing so [76]. Lastly, Lord [77] argued to treat numbers as continuous if this is how they are perceived which arguably is often the case in clinical applications of the LSNS. Hence, these approximations and expected measurement errors of both exposure and outcome measures must be considered in interpreting our results.

Another limitation of the present study is that we did not use clinical diagnoses. Rather, in line with our theoretical underpinnings [30], all our models suppose a continuous spectrum between health and different severities of disease. This is more aligned with novel nosological systems like HiTOP [78] than traditional discrete taxonomies and better supported by empirical evidence on the nature of mental health [79, 80]. Hence, we had symptom severity and continuous markers rather than cases of disease as dependent variables. Therefore, others, that believe in a concrete border between health and disease, might want to replicate these findings with dichotomised outcomes.

Using a population-based sample and employing weights to counteract imbalanced SES sampling ensured a comparatively good generalisability to similar populations. Yet, the extent and effects of social connection are likely strongly contingent upon culture. How large the differences actually are is still uncertain. A systematic review found no differences in the prevalence of social isolation comparing large heterogenous geographic regions like the Americas and Europe [4] but studies comparing prevalences in the Global North with the Global South or between different European regions found stark discrepancies [81, 82]. This inconsistency in results is further complicated by the probable effects of different local norms and interrelations with social desirability bias on response patterns [83].

A further concern is residual confounding. We have conditioned our models on numerous potential confounds to account for this. However, particularly considering the complex interrelations of social connection with multitudinous aspects of human life, residual confounding cannot be ruled out here.

Moreover, the loss to follow-up could have introduced bias, too. The composition of the follow-up sample does not seem to differ from the baseline sample strongly for many key characteristics (gender, age, SES, education), though (see Tables 1 and 2). However, stratified analyses indicated that effect sizes were larger amongst those that only completed one timepoint for some outcomes. Hence, attrition might have biased the effect size to smaller values. Lastly, while our power analysis was very encouraging, it was limited by the shortage of and lack of standardization in the available literature. Thus, we cannot rule out that we missed some subtle nonlinearities in the relationship between LSNS and our outcomes. However, the consistent detection of nonlinear effects of age in the same models at similar effect sizes per unit underscores our ability to detect more complex predictor-outcome relationships at our sample sizes.

## Outlook

Our results show that population strategies are likely to be more effective than individual-level interventions to prevent the adverse health effects of social isolation. While a high-risk strategy might be pursued for other (e.g. political) reasons, our results do not support such an approach. Additionally, the high prevalence of social isolation [10], concerns over stigmatization [6] and the absence of prevention harms [30] suggest a population strategy. Most existing methods to tackle social isolation are individual level interventions, though [67–69]. A literature review recently also concluded that population strategies are gravely under-researched [70].

Scouring the literature for studies of higher-level interventions, we found only sparse examples, too. Interventions that provide spaces and resources to facilitate socialising and collaboration on leisure projects could provide a layout for community-level interventions but have not been investigated as such [84, 85]. Eligibility for free bus passes for elderly citizens seems to improve social connection [86]. Different multi-pronged community-wide interventions in the USA, the Netherlands and Australia were successful to varying degrees [87–89]. Moreover, as most interventions were performed in socially isolated samples, we not only lack evidence on higher-level interventions but also on what might be helpful to socially more integrated individuals.

This highlights a serious need for a lot more research into preventive population strategies. Systemic interventions should strive to target the environmental characteristics that were found to be consistently associated with better social connection. Striving to improve neighbourliness, social cohesion, and social support, public transportation, proximity to resources and to recreational facilities, street walkability, and neighbourhood security are all promising objectives to tackle social isolation on a structural level [90–92]. Additionally, Brandt et al. argued that population-level interventions to reduce prejudices [93], which socially isolate discriminated individuals, constitute a promising strategy to foster social connection [94]. Furthermore, Walsh et al. recently provided recommendation for researchers, funders, science communicators, governments, businesses and public health professionals to accelerate the development of a population strategy of prevention [62]. These are underscored by our research.

Another observation that might prove crucial is the variability in the prevalence of social isolation across the globe [81, 82]. Hence, understanding the cultural and societal factors underlying these differences could be pivotal to fostering social connection in societies struggling with high rates of social isolation.

We have recognized poor social connection as an urgent concern for public health. While existing individual-level interventions are most easily implementable within the structures of our medical systems, our analyses indicate that they are unlikely to constitute an ideal strategy to target social isolation. Given the enormous burden of disease caused by dementia, anxiety and depression, pursuing effective strategies for their prevention is a public health priority. Our research underlines the notion that population strategies against social isolation have great potential to prevent its negative health consequences.

## Supplementary Information

Below is the link to the electronic supplementary material.

Supplementary file1 (DOCX 14954KB)

## Appendix

*Deviations from preregistration*

Although it is standard practice [95], we omitted ICV as a control variable for WMHV in our preregistration. In our analysis, we still included it as a covariate. To simultaneously maintain faithfulness to our preregistration, we compare the results for models not controlled for ICV with the ones reported in Fig S40. No meaningful differences between the models can be discerned. To calculate VIFs we relied on the standard vif() function of the car package instead of the vif.gam() function due to compatibility issues with GAMMs fitted with gamm4 instead of mgcv. Furthermore, we employed other plotting functions for GAMMs and multiple imputation as better and aesthetically more pleasing options came to our attention.

Otherwise, we stuck to our preregistration and only expanded on it for sensitivity analyses and the calculation of population-level shares of harm.

Data availability

This study used third party data. We were granted access to the data from LIFE (Leipziger Forschungszentrum für Zivilisationserkrankungen) under project agreement PV-772. All original data will be exclusively shared by LIFE (https://www.uniklinikum-leipzig.de/einrichtungen/life) based on a project proposal under data protection rules according to the University of Leipzig and can thus not be shared by the authors directly. Please do get in touch if you are planning to do a reproduction and need any assistance with accessing LIFE data. All code can be found at https://github.com/LaurenzLammer/social_isolation_gamms.

*Software*

We performed our analyses using R version 4.4.0 (RRID: SCR_001905). We computed generalized additive mixed models using gamm4 (RRID: SCR_024507), the lme4 based extension of the mgcv package, and performed FDR-correction using the Qvalue package (RRID: SCR_001073). We used mice (RRID: SCR_026363) and its extension miceadds for multiple imputation. Exact information on all software used can be found in the published code.

Different Linux kernels and Ubuntu versions constituted the computational infrastructure during the data processing and analysis.

*Imaging protocols and quality control*

We obtained T1-images using a 3D MPRAGE protocol and the following parameters: inversion time, 900 ms; repetition time, 2300 ms; echo time, 2.98 ms; flip angle, 9°; field of view, 256 × 240 × 176 mm3; voxel size, 1 × 1 × 1 mm3, shimming: tune-up shim, no fat suppression, whole-brain coverage including cerebellum. A fluid-attenuated inversion-recovery (FLAIR) sequence (TR = 5000 ms, TI = 1800 ms, TE = 395 ms, 1 × 0.49 × 0.49 mm resolution, AT = 7.02 min) was obtained for lesion sensitive images in the same session.

No mock scanning was performed. The MRI was operated by a single technician with a colleague next door in case of emergency. The entire session lasted around one hour as up to four more modalities not used in this study were obtained.

*Quality control*

Study doctors examined all MRI scans and evaluated whether they were unusable due to brain tumors, or acute ischaemic, haemorrhagic or traumatic lesions. In case of incidental findings, we contacted the patients and informed them. We only excluded these participants if they fulfilled the abovementioned exclusion criteria.

Additionally, we relied on the visual quality control ratings for FLAIR images from a previous study. Therein, we checked whether the lesions marked in voxel-wise maps of lesion change were confounded due to poor scan quality, lesion regression, or brain pathologies at baseline or follow-up. We gave the following LCL quality ratings: quality concerns due to motion (LCL quality = 1), ventricular expansion which led to regression of lesion voxels (LCL quality = 2), and brain pathologies such as stroke or congenital lesions (LCL quality = 3) [39].

*MRI processing*

In participants for which a T1 and a FLAIR were available, we used both contrasts. To this end, we coregistered FLAIRs with and reformatted them to the T1 scans. For these participants, we differentiated the pallidum from the white matter as suggested by Freesurfer. If both contrasts were available for both timepoints, we processed them with the algorithm’s longitudinal pipeline and created an individual template based on the T1 scans prior to coregistration and reformatting. If a FLAIR was missing at one timepoint, the longitudinal pipeline could not be applied.

*Imputation*

We set the maximum iteration number to 25. Not all participants underwent the entire set of examinations. Only a subcohort of the participants got MRIs and extensive cognitive testing. Furthermore, not all participants returned for follow-up. We only imputed missing values for participants that were in the relevant subcohort. Thus, we did not impute missing MRI values for participants that were not in the neurocognitive subcohort or follow-up measures of individuals that did not participate in the follow-up. If values for MRI measures have been excluded due to poor quality, we did not impute these values but excluded them from our analyses. Based on the existing literature, we had predefined a maximum missingness rate of 40% that was not reached for any variable [96–98].

We employed predictive mean matching with 25 donors for continuous variables to avoid imputing impossible scores (e.g. negative LSNS scores) and a logistic regression model for binary variables. We used linear mixed models with participant as the clustering variable if sufficient data was available to reach convergence.

Predicting dependent variables of our models by our predictor of interest LSNS as a linear variable would bias the resulting datasets in favour of a linear relationship. Hence, we predicted missing values in our outcome variables by social isolation both as a continuous score and a categorical variable. The predictor matrix and specific imputation method for all relevant variables are visualised in Fig S1.

We imputed the default number of five datasets. As there is no direct analogue to Rubin’s rule to pool the results of GAMMs, we preregistered that we will primarily report the results of the first imputed dataset as in a stochastic regression imputation. To represent the greater uncertainty of the results we additionally illustrate the range of results across imputations.

As ICV is invariable intrapersonally, we imputed missing values with those of another observation of the same participant if available prior to multiple imputation.

*Diagnostics*

Plots of means and SDs of all imputed variables show the absence of a trend at 25 iterations (see Fig S2). Figs S3-4 allow the comparison of original and imputed data. Additionally, Fig S5 illustrates the missing data patterns.

*Power analysis*

We used the standard LSNS threshold of < 12. We simulated 100 datasets with each including 5 LSNS-outcome relationships for each of the seven outcomes:

a) A linear relationship across the entire spectrum of LSNS scores.

b) A linear relationship across the entire spectrum and an additional exponential effect below the threshold.

c) A linear relationship across the entire spectrum and an additional linear effect below the threshold.

d) An exponential effect but only below the threshold.

e) A linear effect but only below the threshold.

This power analysis was based on two assumptions:

1) The *average* slope of social isolation should be equal to the effect size detected in linear regressions found in the literature.

2) In nonlinear cases in which there is a non-zero effect below the threshold, the average slope below the threshold should be three times as large as above to constitute a meaningful difference between the two sides of the threshold.

We estimated the effect sizes based on existing literature of the effect of LSNS on our outcomes [32, 99–101] and used differential equations and accounted for the distribution of LSNS scores to adhere to the above stated assumptions.

As in our main analyses we relied on anova to compare *full* and *base* models and based our judgements on the returned BICs and p-values uncorrected for multiple comparisons (α < 0.05).

Please refer to the preregistration for details on the effect size estimation, the mathematics underlying the modelling of the relationships and results differentiated by outcome and relationship form.

*Reflexivity*

I, as the first author of this manuscript, want to go beyond a mere statement of financial conflicts of interest and expand this study by a brief and transparent reflection on influences that might have played a role in the formation of this study [38]. In my medical dissertation, I studied social isolation as a risk factor for cognitive decline and dementia. Considering the amounting epidemiological evidence showing social isolation’s relevance, I came to wonder whether epidemiology could also assist us in understanding how to tackle it. Encountering Rose’s *The Strategy of Preventive Medicine* [30], I had found the theoretical framework for this endeavour. As Rose, I am convinced that the commonly applied high-risk strategy of prevention is limited and that population strategies often hold greater preventive potential. Population strategies aim to act on societal conditions in the realms of economy, industry, culture and politics [102]. They often target issues like socioeconomic inequalities, institutional racism, ageism or social cohesion [62] and thus tend to be aligned with my personal political convictions. I would insist that I hold these convictions for the very reason that they are sensible policies with beneficial effects on society and its members. Yet, researchers should always be particularly attentive of their standpoint in case of a potential coincidence of research implications and personal political opinion. Therefore, the study was thoroughly preregistered to outcome-independently predetermine how results and their interpretation would be arrived at [103]. Furthermore, I chose GAMMs as a shape agnostic approach to modelling the relationship of social isolation and our outcomes to avert biasing the results in any direction. Nevertheless, other researchers with different standpoints could have pursued alternative approaches and I would very much welcome an adversarial collaboration [104].

Beyond political implications, my thinking about the phenomena investigated in this study is of course shaped by the patients and those close to me in my private life that have been or are affected by them. Unfortunately, social isolation, dementia, anxiety and depressive disorders are so pervasive in our society that I had to encounter all of them in both my private and professional life. Thankfully, I never had to experience any of them first-hand.
