# Supplementary material for: Generalized additive mixed models to discern data-driven theoretically informed strategies for public brain, cognitive and mental health
Source: Eur J Epidemiol. 2025 Sep 15;40(11):1323–43. doi: 10.1007/s10654-025-01296-9 (PMC12695978; doi:10.1007/s10654-025-01296-9)
Supplement: Supplementary file 1 — Supplementary file1 (DOCX 14954KB) [file 10654_2025_1296_MOESM1_ESM.docx]

Supplementary figures

**Fig S1**


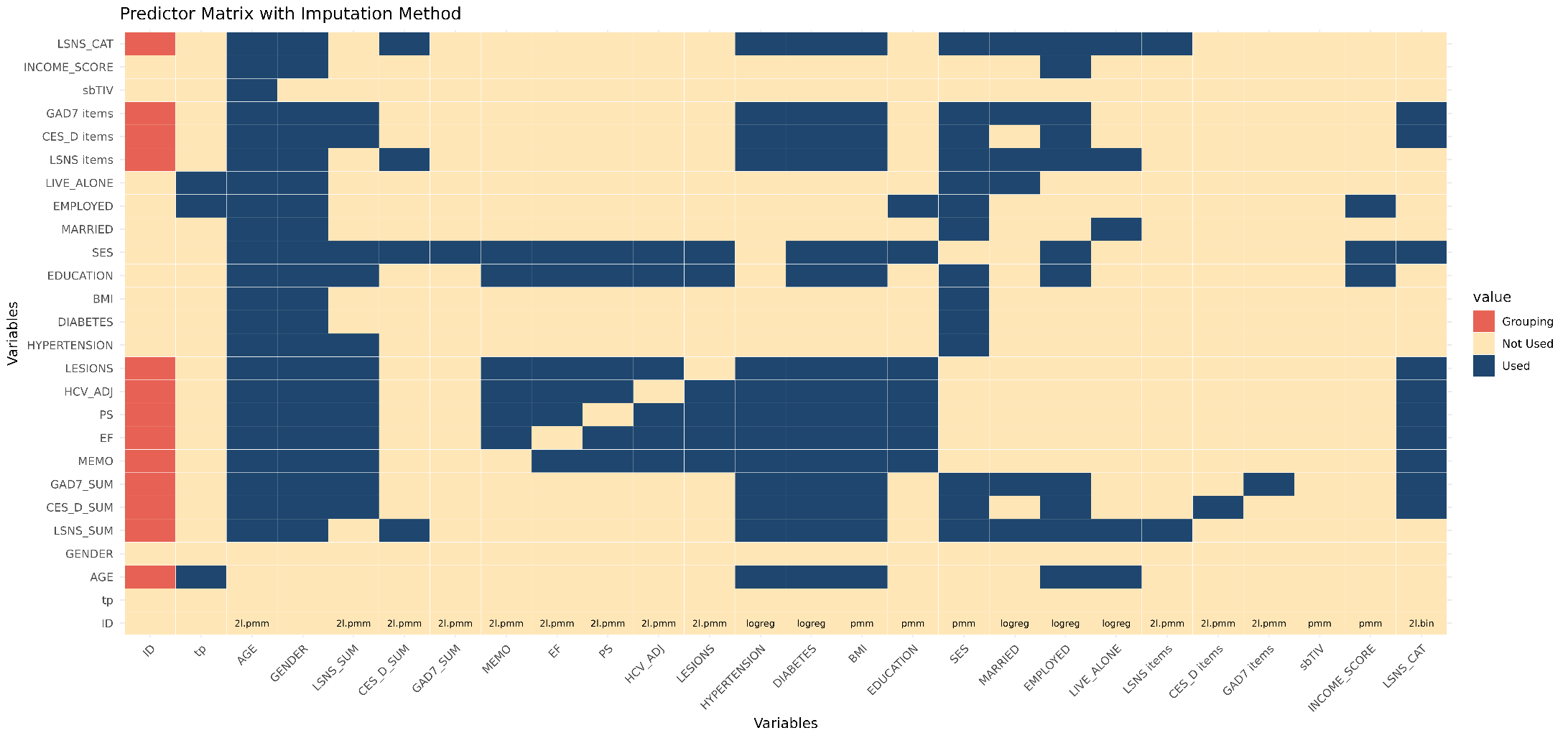


*Supplementary Fig. 1: Predictor matrix and method used for multiple imputation for each variable. tp = timepoint; LSNS_SUM = sum score of the Lubben Social Network Scale; CES_D_SUM = sum score of the Centre for Epidemiologic Studies Depression Scale; GAD7_SUM = sum score of the 7-item Generalized Anxiety Disease questionnaire; MEMO = memory; EF = executive functions; PS = processing speed; HCV_ADJ = adjusted hippocampal volume; LESIONS = white matter hyperintensity/lesion volume; BMI = body-mass-index; SES = socioeconomic status; LIVE_ALONE = living alone; sbTIV = total intracranial volume; LSNS_CAT = categorical dichotomous LSNS*

**Fig S2**


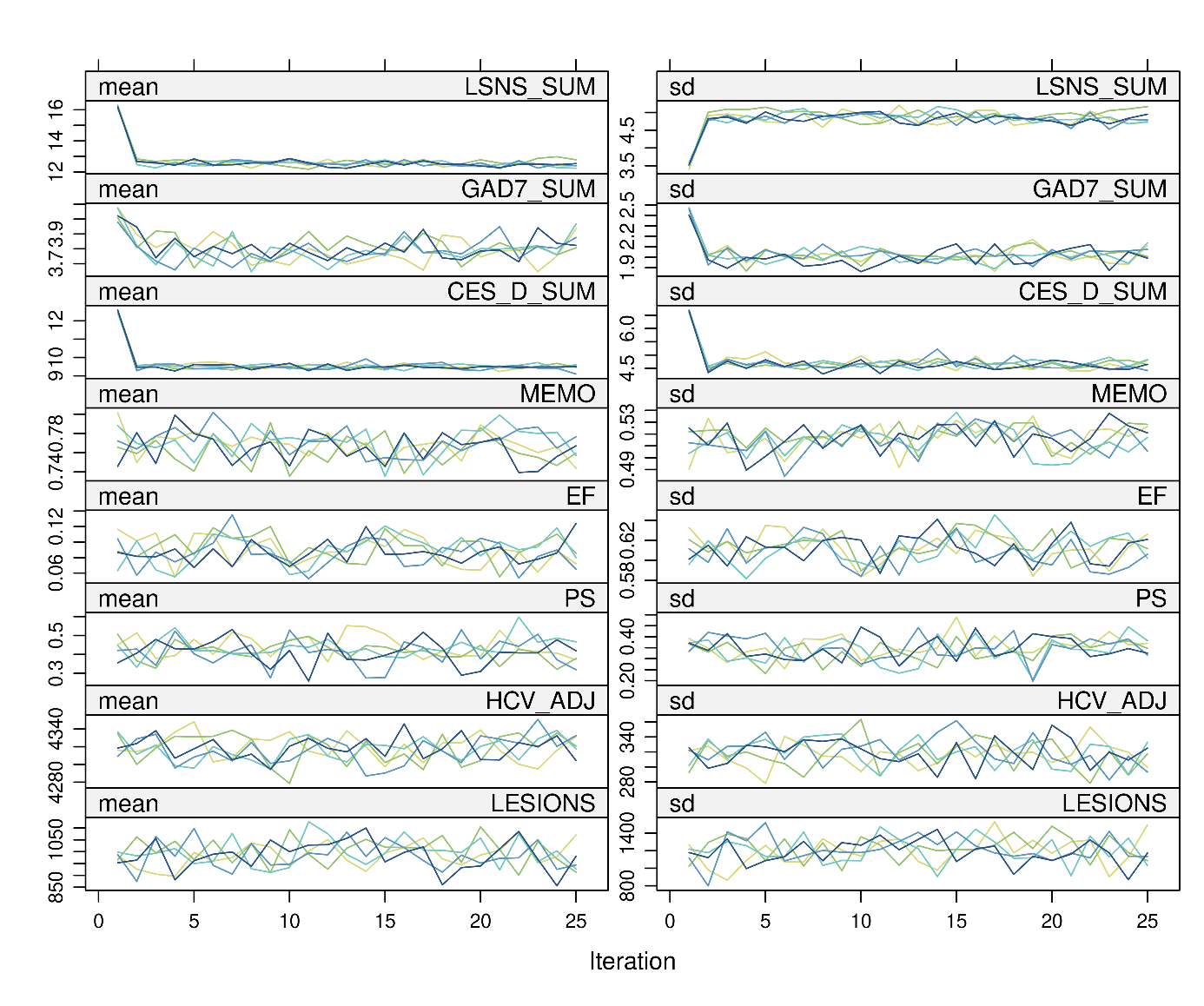


*Supplementary Fig. 2: Means and standard deviations over the course of 25 iterations. The plots show the absence of a trend at the maximum iteration number. LSNS_SUM = sum score of the Lubben Social Network Scale; CES_D_SUM = sum score of the Centre for Epidemiologic Studies Depression Scale; GAD7_SUM = sum score of the 7-item Generalized Anxiety Disease questionnaire; MEMO = memory; EF = executive functions; PS = processing speed; HCV_ADJ = adjusted hippocampal volume; LESIONS = white matter hyperintensity/lesion volume*

**Fig S3**
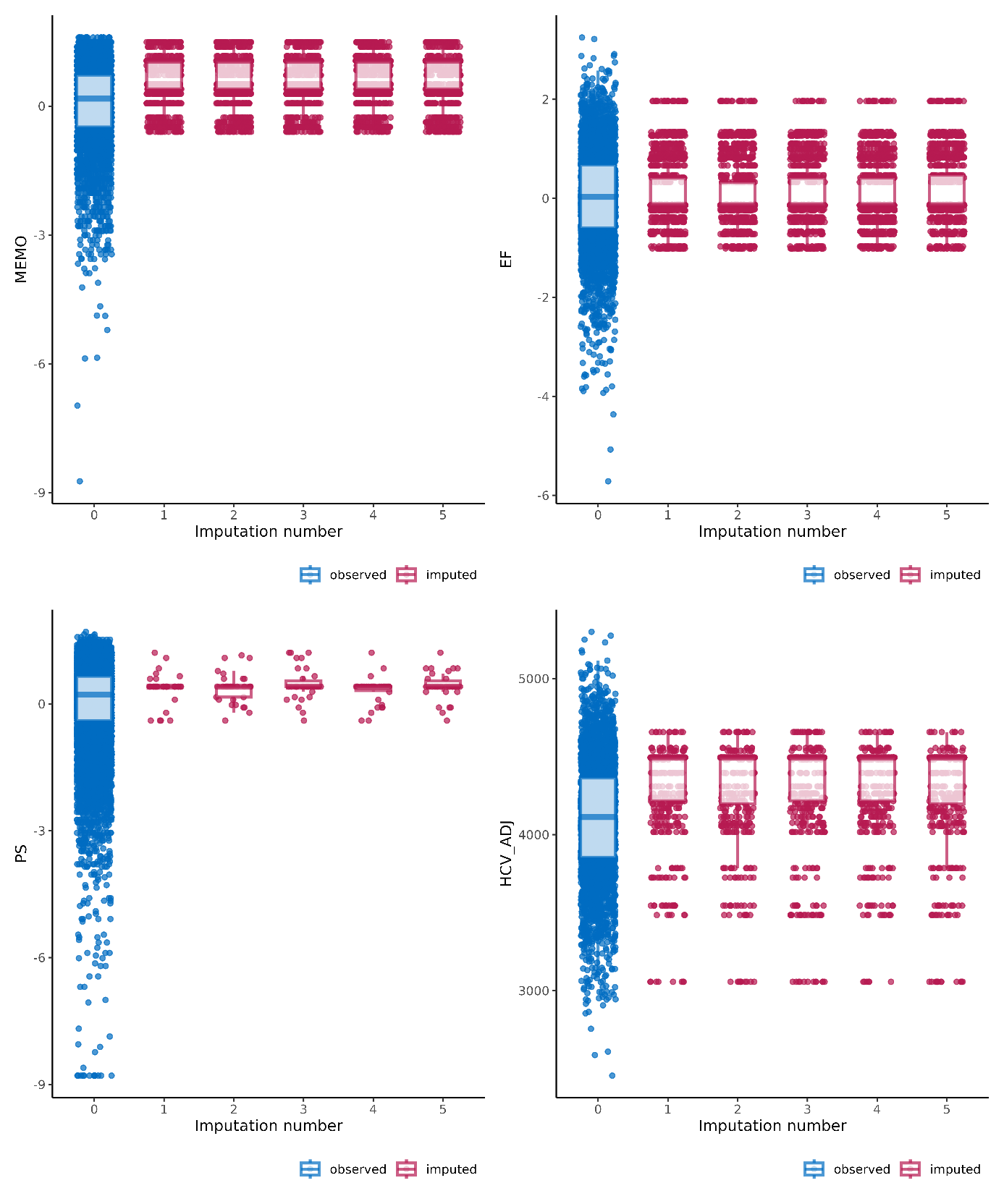


*Supplementary Fig. 3: Comparison of observed and variables imputed with predictive mean matching and 25 donors. The boxplots show the median and the lower and upper hinges correspond to the first and third quartiles (the 25th and 75th percentiles). MEMO = memory; EF = executive functions; PS = processing speed; HCV_ADJ = adjusted hippocampal volume.*

**Fig S4**
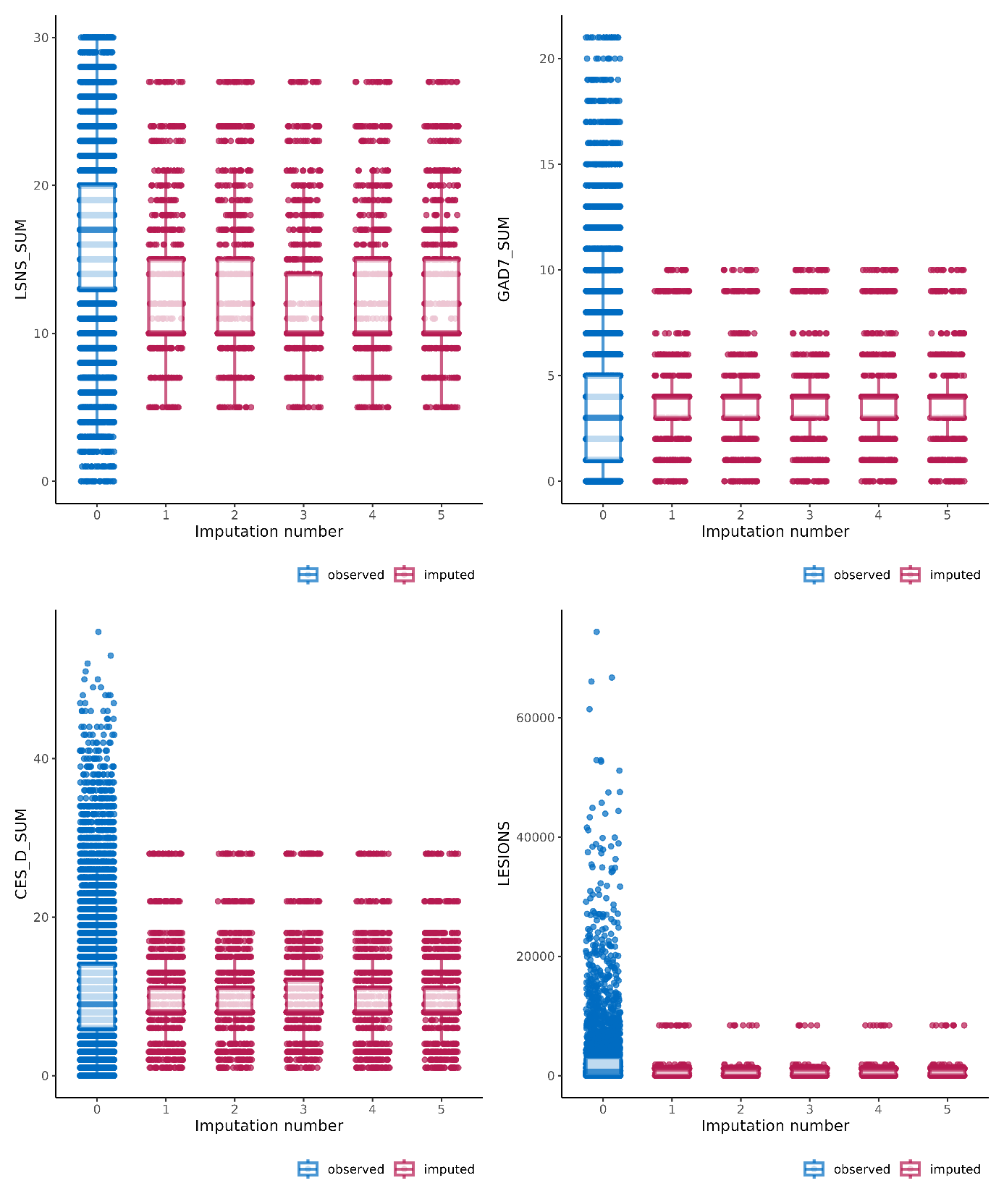


*Supplementary Fig. 4: Comparison of observed and variables imputed with predictive mean matching and 25 donors. The boxplots show the median and the lower and upper hinges correspond to the first and third quartiles (the 25th and 75th percentiles). LSNS_SUM = sum score of the Lubben Social Network Scale; CES_D_SUM = sum score of the Centre for Epidemiologic Studies Depression Scale; GAD7_SUM = sum score of the 7-item Generalized Anxiety Disease questionnaire; LESIONS = white matter hyperintensity/lesion volume*

**Fig S5**
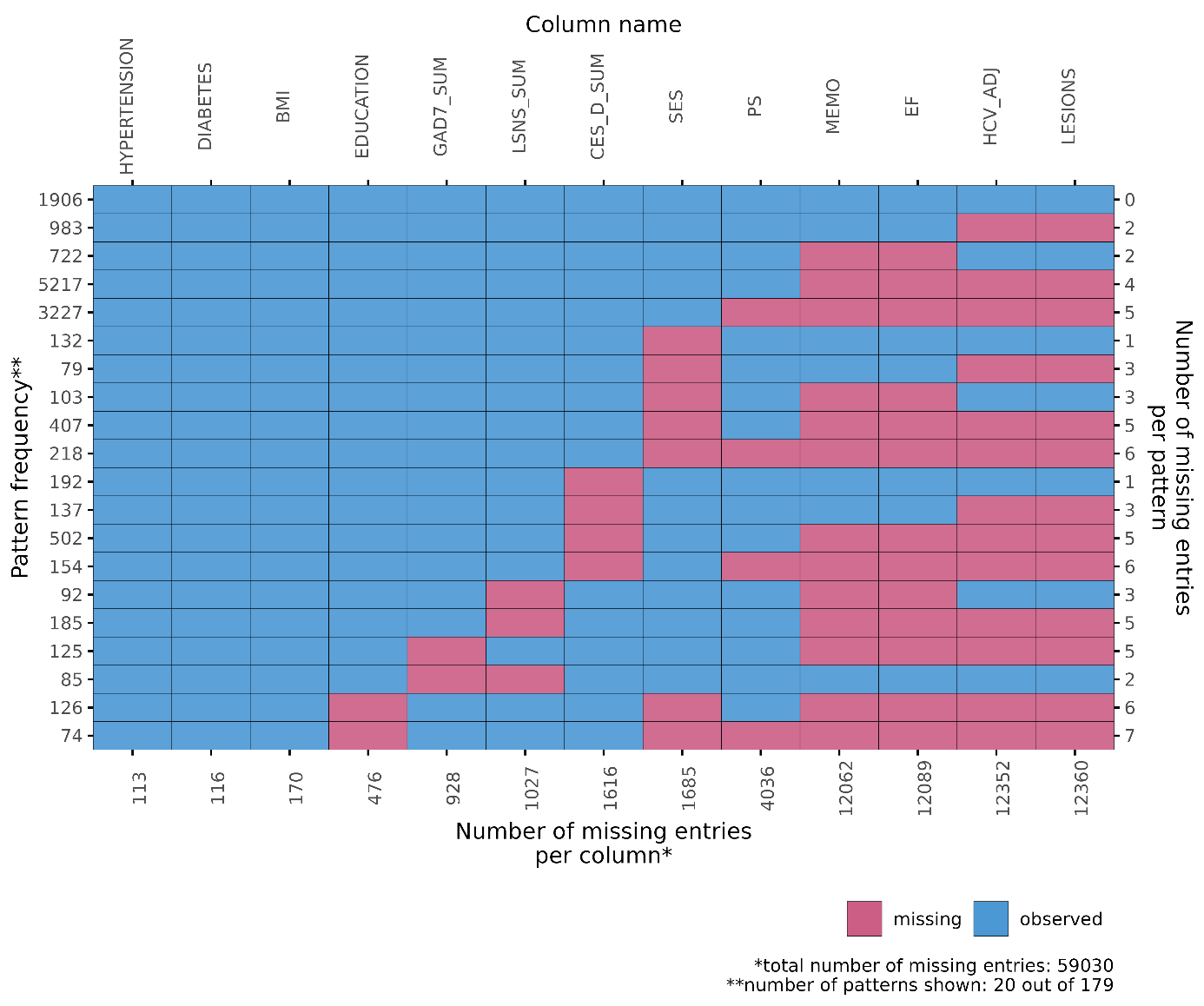


*Supplementary Fig. 5: Missingness pattern of the observed dataset. LSNS_SUM = sum score of the Lubben Social Network Scale; CES_D_SUM = sum score of the Centre for Epidemiologic Studies Depression Scale; GAD7_SUM = sum score of the 7-item Generalized Anxiety Disease questionnaire; MEMO = memory; EF = executive functions; PS = processing speed; HCV_ADJ = adjusted hippocampal volume; LESIONS = white matter hyperintensity/lesion volume; BMI = body-mass-index; SES = socioeconomic status*

**Fig S6**

**
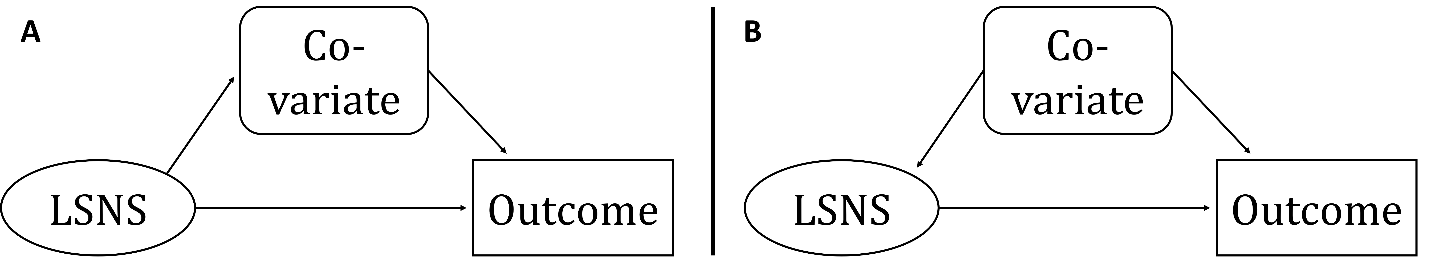
**

*Supplementary Fig. 6: A) The covariate mediates the effect of LSNS on the outcome variable. Hence, the variable should not be controlled for. B) The covariate affects both LSNS and the outcome. Hence, it is a confounder and should be controlled for*

**Fig S7**


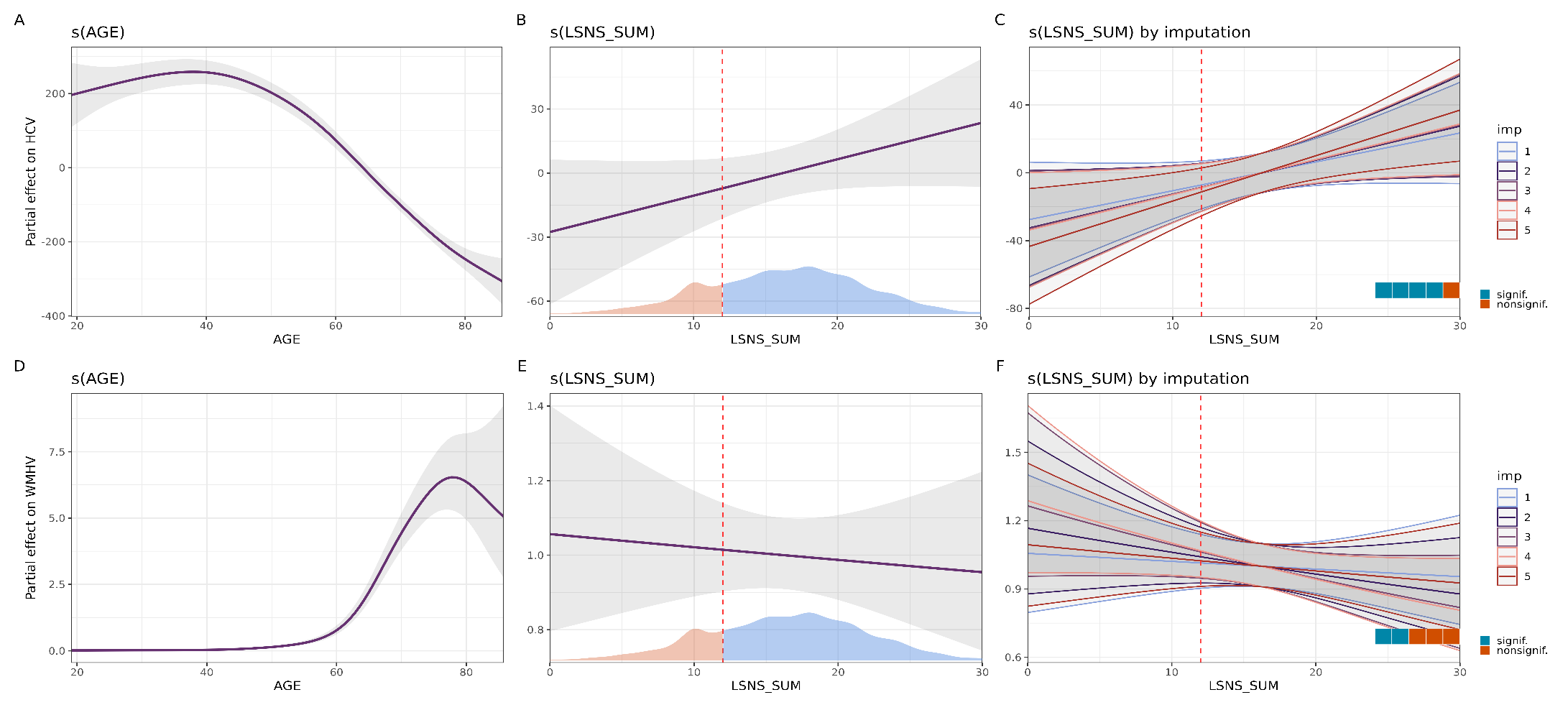


*Supplementary Fig. 7: Results of model 2. A) The partial effect of age on hippocampal volume (HCV in the 1st imputed dataset). B) The partial effect of Lubben Social Network Scale (LSNS) scores on hippocampal volume (HCV) in the 1st imputed dataset. The dashed vertical line illustrates the standard LSNS cut-off. The density plot at the bottom depicts the distribution of LSNS scores with those considered socially isolated shown in red. C) Partial effects of LSNS scores on HCV across the five imputations. The waffle plot indicated that the FDR-corrected q-values were significant in four of five imputations. D) The partial effect of age on white matter hyperintensity volume (WMHV) in the 1st imputed dataset. E) The partial effect of Lubben Social Network Scale (LSNS) scores on white matter hyperintensity volume in the 1st imputed dataset. The dashed vertical line illustrates the standard LSNS cut-off. The density plot at the bottom depicts the distribution of LSNS scores with those considered socially isolated shown in red. F) Partial effects of LSNS scores on white matter hyperintensity volume across the five imputations. The waffle plot indicated that the FDR-corrected q-values were significant in two of five imputations.*

** < 0.05; ** < 0.01; *** < 0.001. HCV and WMHV are measured in mm3. Grey areas indicate 95% confidence intervals.*

**Fig S8****
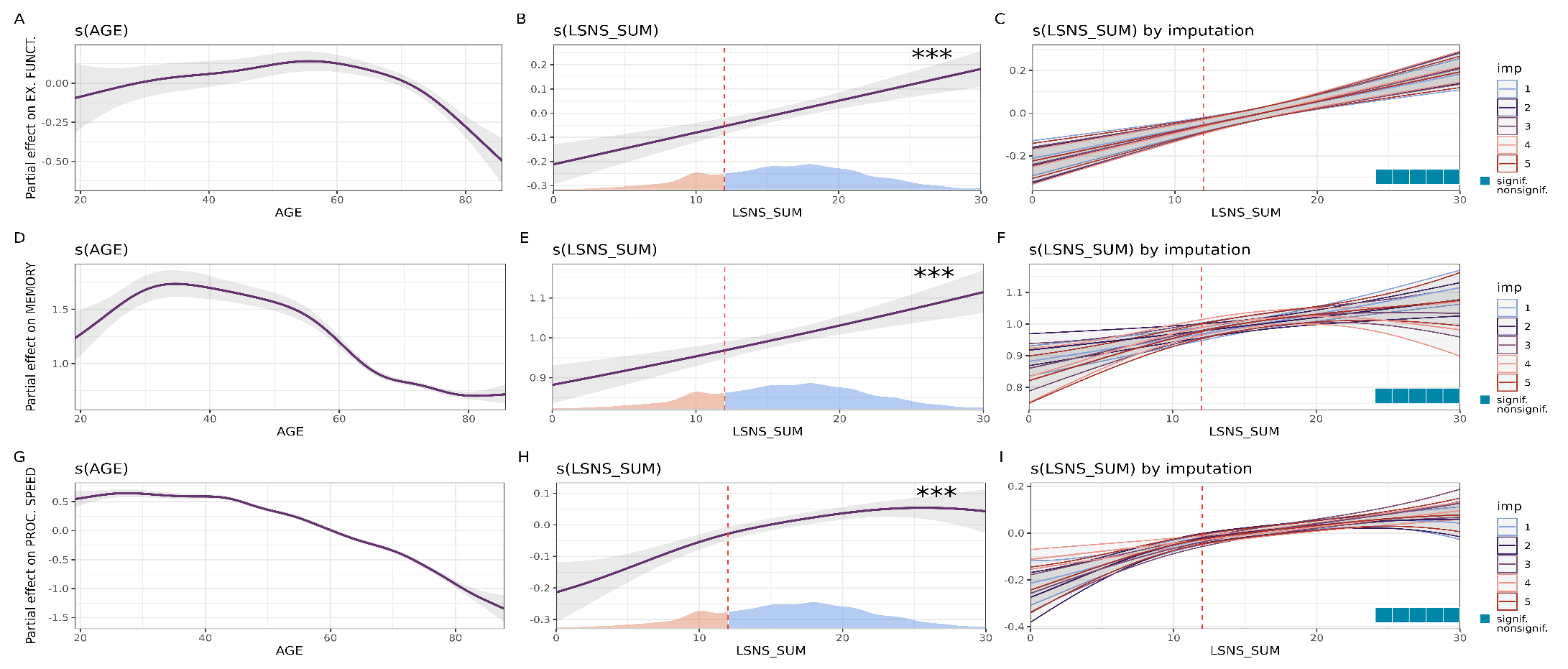
**

*Supplementary Fig. 8: Results of model 2. A) The partial effect of age on executive functions in the 1st imputed dataset. B) The partial effect of Lubben Social Network Scale (LSNS) scores on executive functions in the 1st imputed dataset. The dashed vertical line illustrates the standard LSNS cut-off. The density plot at the bottom depicts the distribution of LSNS scores with those considered socially isolated shown in red. C) Partial effects of LSNS scores on executive functions across the five imputations. The waffle plot indicated that the FDR-corrected q-values were significant in all five imputations. D) The partial effect of age on memory in the 1st imputed dataset. E) The partial effect of Lubben Social Network Scale (LSNS) scores on memory in the 1st imputed dataset. The dashed vertical line illustrates the standard LSNS cut-off. The density plot at the bottom depicts the distribution of LSNS scores with those considered socially isolated shown in red. F) Partial effects of LSNS scores on memory across the five imputations. The waffle plot indicated that the FDR-corrected q-values were significant in all five imputations. G) The partial effect of age on processing speed in the 1st imputed dataset. H) The partial effect of Lubben Social Network Scale (LSNS) scores on processing speed in the 1st imputed dataset. The dashed vertical line illustrates the standard LSNS cut-off. The density plot at the bottom depicts the distribution of LSNS scores with those considered socially isolated shown in red. I) Partial effects of LSNS scores on processing speed across the five imputations. The waffle plot indicated that the FDR-corrected q-values were significant in all five imputations.*

** < 0.05; ** < 0.01; *** < 0.001. Cognitive functions are measured in standard deviations. Grey areas indicate 95% confidence intervals.*

**Fig S9**

**
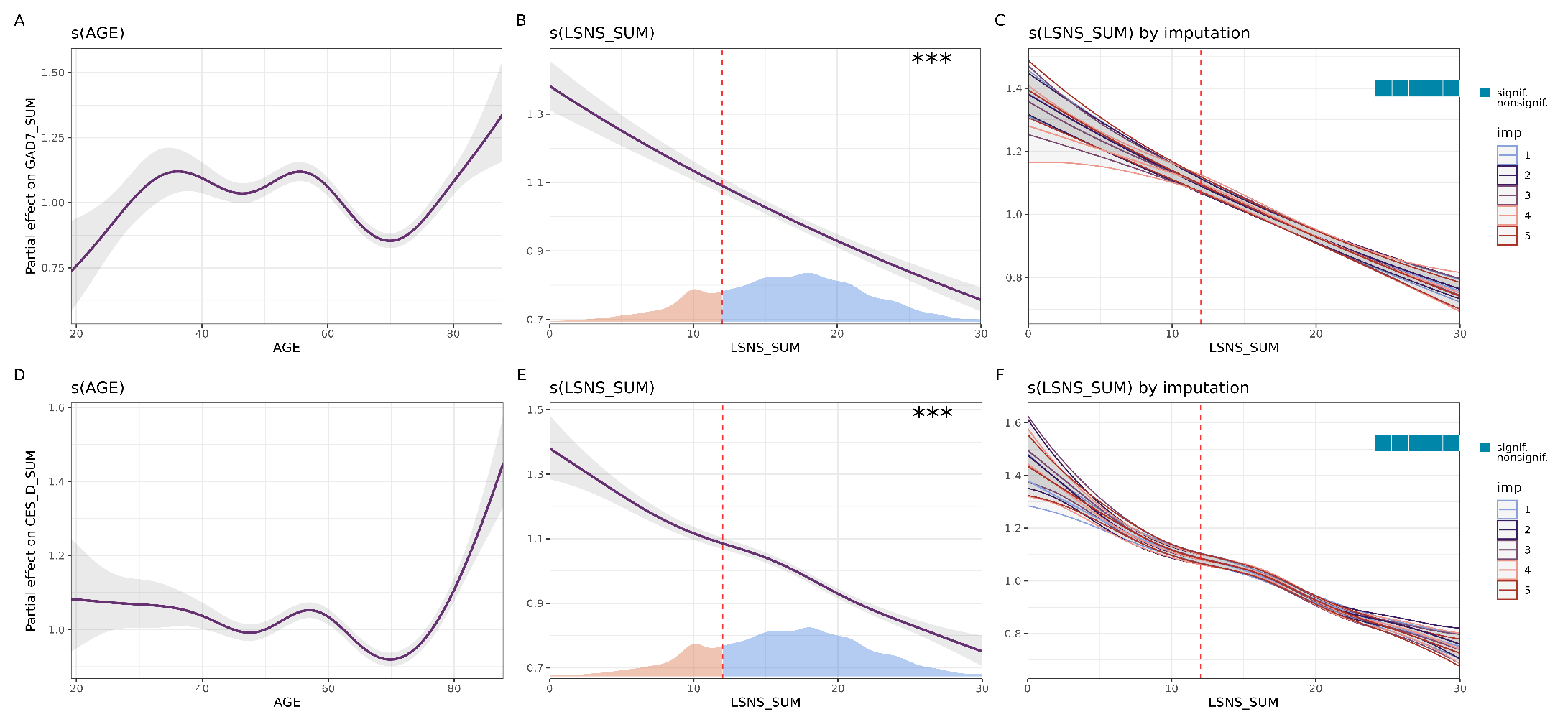
**

*Supplementary Fig. 9: Results of model 2. A) The partial effect of age on anxiety symptoms in the 1st imputed dataset. B) The partial effect of Lubben Social Network Scale (LSNS) scores on anxiety symptoms in the 1st imputed dataset. The dashed vertical line illustrates the standard LSNS cut-off. The density plot at the bottom depicts the distribution of LSNS scores with those considered socially isolated shown in red. C) Partial effects of LSNS scores on anxiety symptoms across the five imputations. The waffle plot indicated that the FDR-corrected q-values were significant in all five imputations. D) The partial effect of age on depressive symptoms in the 1st imputed dataset. E) The partial effect of Lubben Social Network Scale (LSNS) scores on depressive symptoms in the 1st imputed dataset. The dashed vertical line illustrates the standard LSNS cut-off. The density plot at the bottom depicts the distribution of LSNS scores with those considered socially isolated shown in red. F) Partial effects of LSNS scores on depressive symptoms across the five imputations. The waffle plot indicated that the FDR-corrected q-values were significant in all five imputations*

** < 0.05; ** < 0.01; *** < 0.001. GAD7 and CESD are measured in points on the respective questionnaire. Grey areas indicate 95% confidence intervals.*

**Fig S10**
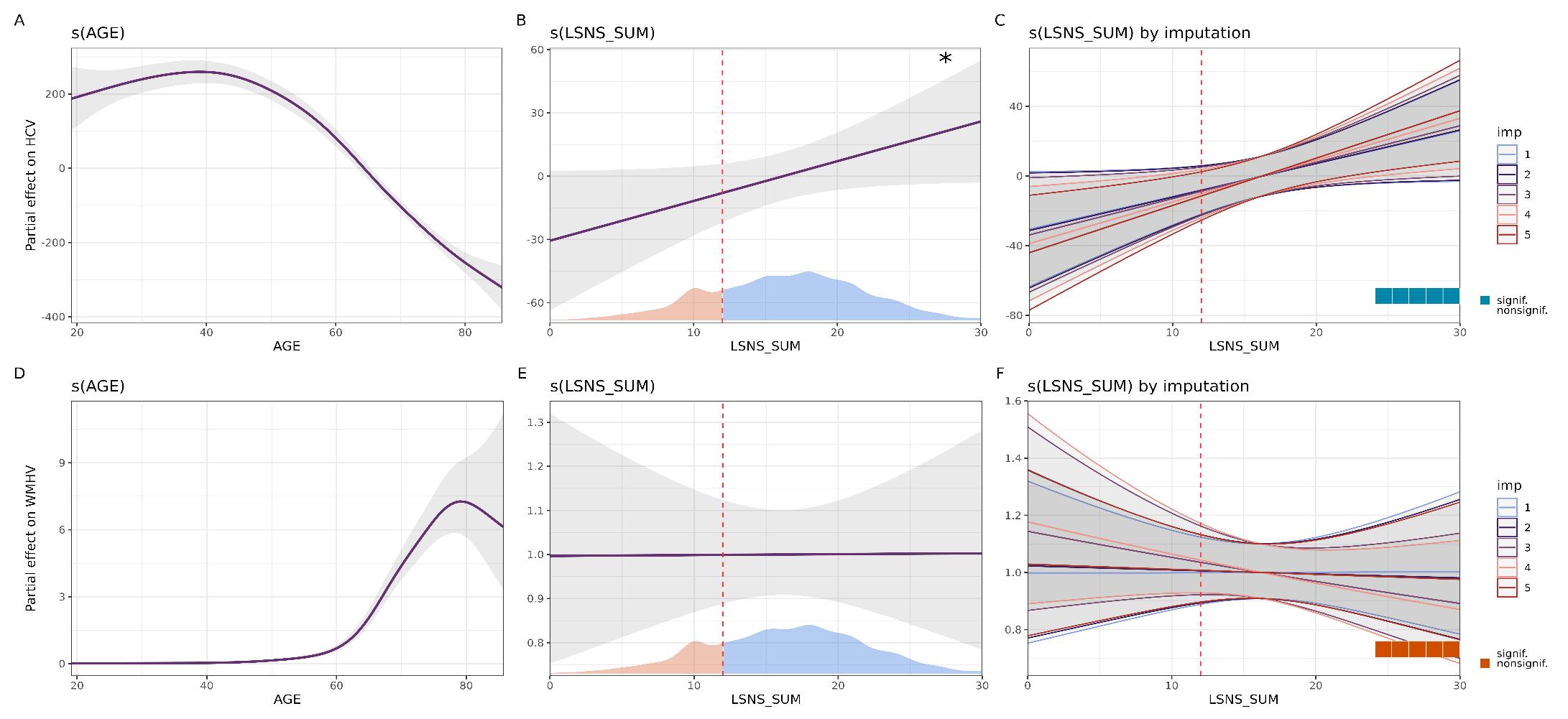


*Supplementary Fig. 10: Results of unweighted model 1. A) The partial effect of age on hippocampal volume (HCV in the 1st imputed dataset). B) The partial effect of Lubben Social Network Scale (LSNS) scores on hippocampal volume (HCV) in the 1st imputed dataset. The dashed vertical line illustrates the standard LSNS cut-off. The density plot at the bottom depicts the distribution of LSNS scores with those considered socially isolated shown in red. C) Partial effects of LSNS scores on HCV across the five imputations. The waffle plot indicated that the FDR-corrected q-values were significant in four of five imputations. D) The partial effect of age on white matter hyperintensity volume (WMHV) in the 1st imputed dataset. E) The partial effect of Lubben Social Network Scale (LSNS) scores on white matter hyperintensity volume in the 1st imputed dataset. The dashed vertical line illustrates the standard LSNS cut-off. The density plot at the bottom depicts the distribution of LSNS scores with those considered socially isolated shown in red. F) Partial effects of LSNS scores on white matter hyperintensity volume across the five imputations. The waffle plot indicated that the FDR-corrected q-values were significant in none of five imputations.*

** < 0.05; ** < 0.01; *** < 0.001. HCV and WMHV are measured in mm3. Grey areas indicate 95% confidence intervals.*

**Fig S11**

**
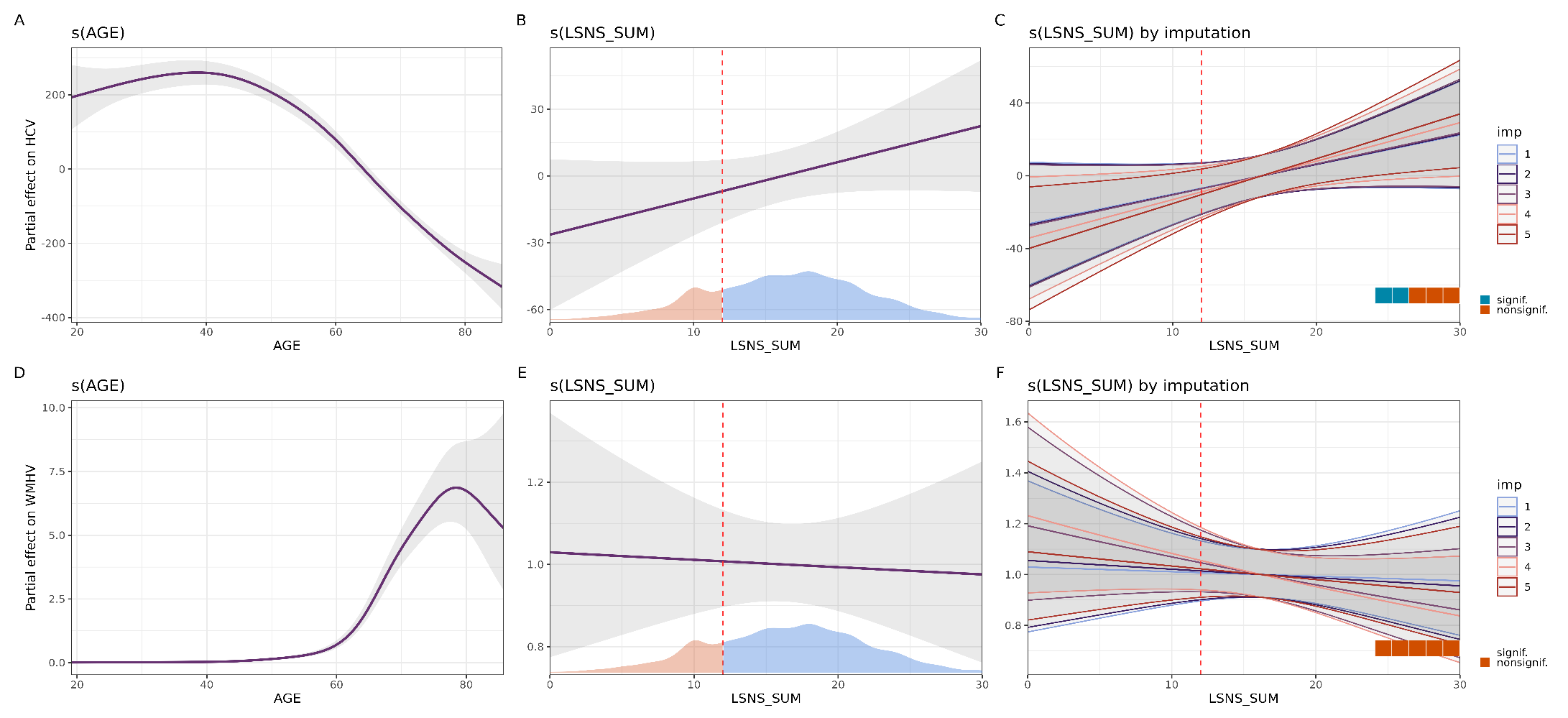
**

*Supplementary Fig. 11: Results of unweighted model 2. A) The partial effect of age on hippocampal volume (HCV in the 1st imputed dataset). B) The partial effect of Lubben Social Network Scale (LSNS) scores on hippocampal volume (HCV) in the 1st imputed dataset. The dashed vertical line illustrates the standard LSNS cut-off. The density plot at the bottom depicts the distribution of LSNS scores with those considered socially isolated shown in red. C) Partial effects of LSNS scores on HCV across the five imputations. The waffle plot indicated that the FDR-corrected q-values were significant in two of five imputations. D) The partial effect of age on white matter hyperintensity volume (WMHV) in the 1st imputed dataset. E) The partial effect of Lubben Social Network Scale (LSNS) scores on white matter hyperintensity volume in the 1st imputed dataset. The dashed vertical line illustrates the standard LSNS cut-off. The density plot at the bottom depicts the distribution of LSNS scores with those considered socially isolated shown in red. F) Partial effects of LSNS scores on white matter hyperintensity volume across the five imputations. The waffle plot indicated that the FDR-corrected q-values were significant in none of five imputations.*

** < 0.05; ** < 0.01; *** < 0.001. HCV and WMHV are measured in mm3. Grey areas indicate 95% confidence intervals.*

**Fig S12****
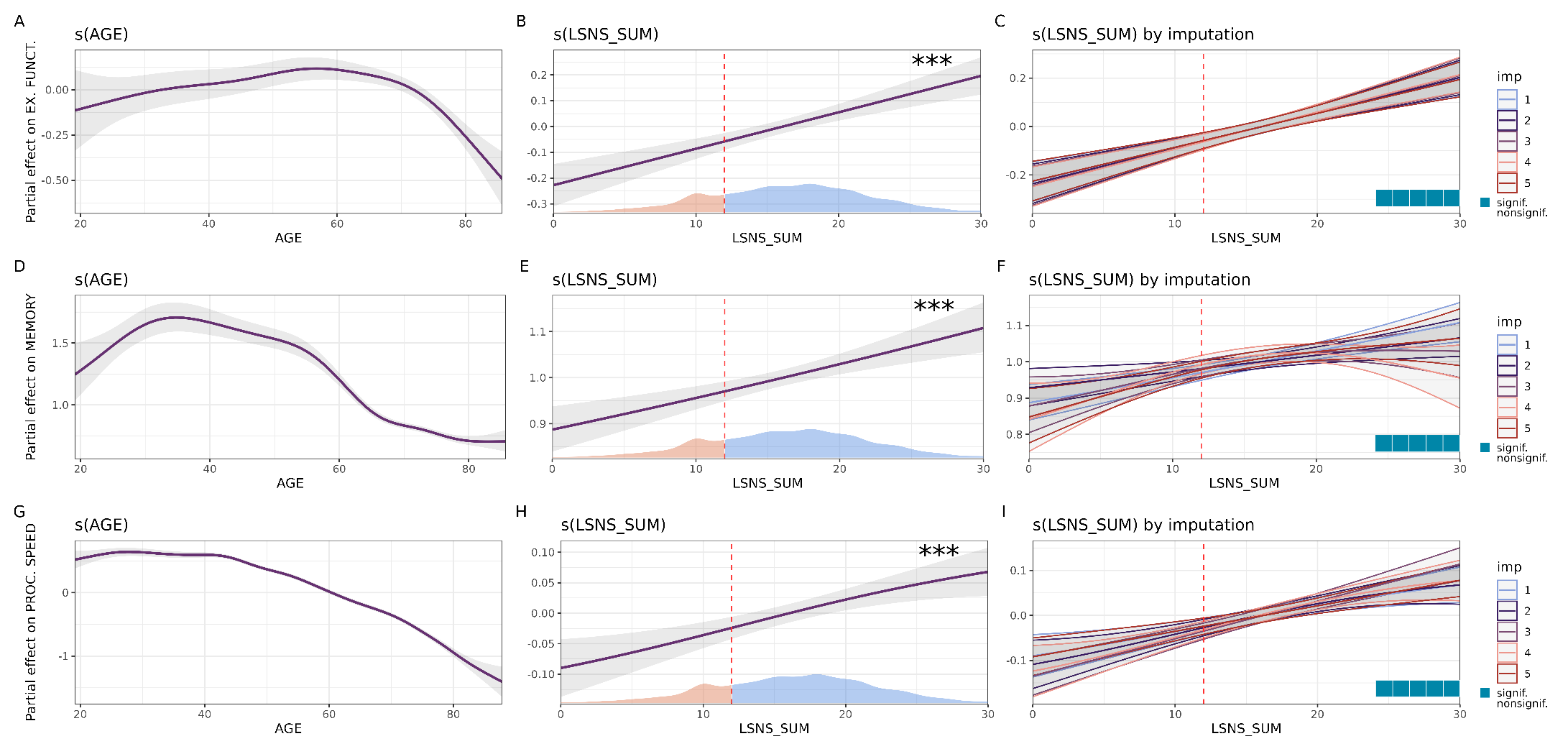
**

*Supplementary Fig. 12: Results of unweighted model 1. A) The partial effect of age on executive functions in the 1st imputed dataset. B) The partial effect of Lubben Social Network Scale (LSNS) scores on executive functions in the 1st imputed dataset. The dashed vertical line illustrates the standard LSNS cut-off. The density plot at the bottom depicts the distribution of LSNS scores with those considered socially isolated shown in red. C) Partial effects of LSNS scores on executive functions across the five imputations. The waffle plot indicated that the FDR-corrected q-values were significant in all five imputations. D) The partial effect of age on memory in the 1st imputed dataset. E) The partial effect of Lubben Social Network Scale (LSNS) scores on memory in the 1st imputed dataset. The dashed vertical line illustrates the standard LSNS cut-off. The density plot at the bottom depicts the distribution of LSNS scores with those considered socially isolated shown in red. F) Partial effects of LSNS scores on memory across the five imputations. The waffle plot indicated that the FDR-corrected q-values were significant in all five imputations. G) The partial effect of age on processing speed in the 1st imputed dataset. H) The partial effect of Lubben Social Network Scale (LSNS) scores on processing speed in the 1st imputed dataset. The dashed vertical line illustrates the standard LSNS cut-off. The density plot at the bottom depicts the distribution of LSNS scores with those considered socially isolated shown in red. I) Partial effects of LSNS scores on processing speed across the five imputations. The waffle plot indicated that the FDR-corrected q-values were significant in all five imputations.*

** < 0.05; ** < 0.01; *** < 0.001. Cognitive functions are measured in standard deviations. Grey areas indicate 95% confidence intervals.*

**Fig S13****
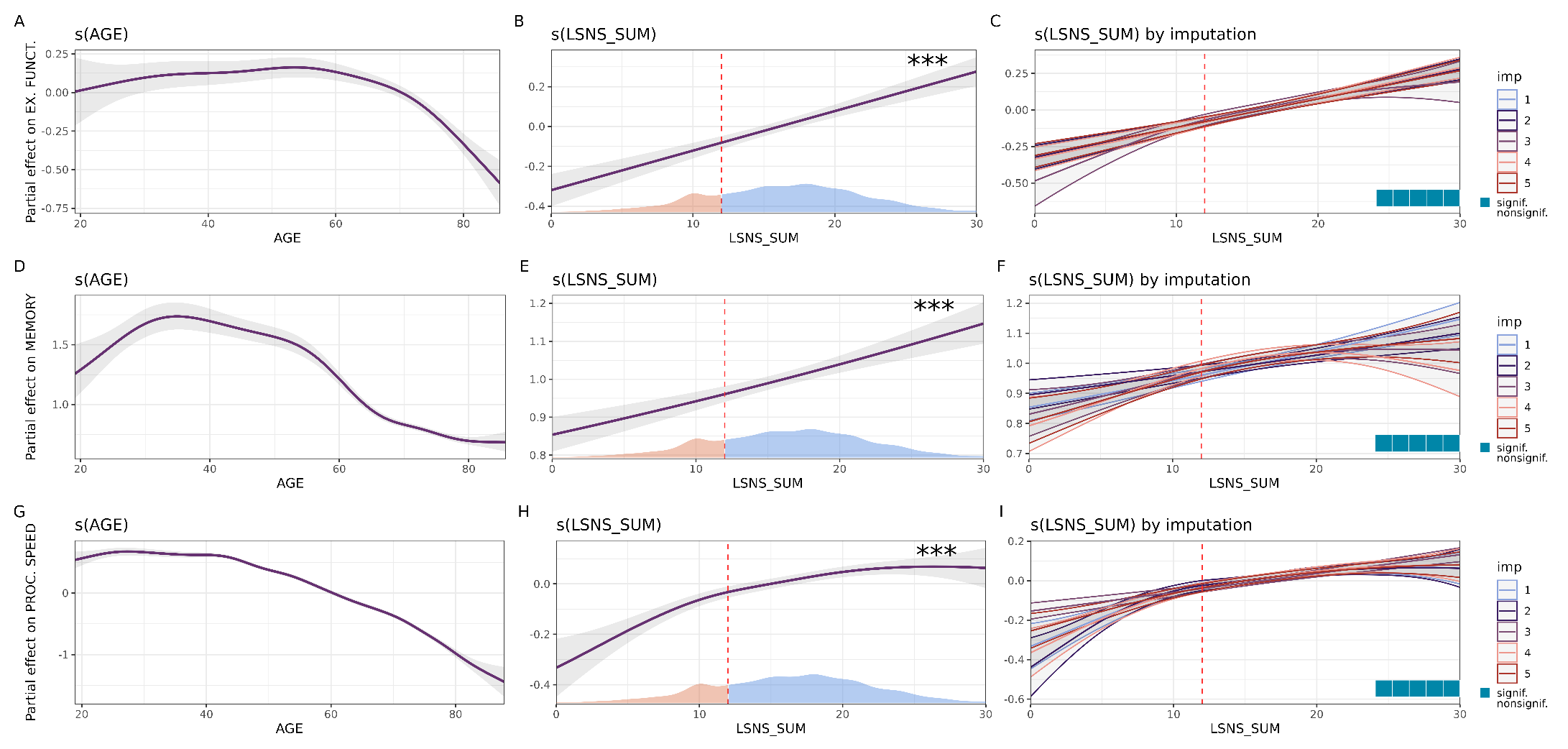
**

*Supplementary Fig. 13: Results of model 2. A) The partial effect of age on executive functions in the 1st imputed dataset. B) The partial effect of Lubben Social Network Scale (LSNS) scores on executive functions in the 1st imputed dataset. The dashed vertical line illustrates the standard LSNS cut-off. The density plot at the bottom depicts the distribution of LSNS scores with those considered socially isolated shown in red. C) Partial effects of LSNS scores on executive functions across the five imputations. The waffle plot indicated that the FDR-corrected q-values were significant in all five imputations. D) The partial effect of age on memory in the 1st imputed dataset. E) The partial effect of Lubben Social Network Scale (LSNS) scores on memory in the 1st imputed dataset. The dashed vertical line illustrates the standard LSNS cut-off. The density plot at the bottom depicts the distribution of LSNS scores with those considered socially isolated shown in red. F) Partial effects of LSNS scores on memory across the five imputations. The waffle plot indicated that the FDR-corrected q-values were significant in all five imputations. G) The partial effect of age on processing speed in the 1st imputed dataset. H) The partial effect of Lubben Social Network Scale (LSNS) scores on processing speed in the 1st imputed dataset. The dashed vertical line illustrates the standard LSNS cut-off. The density plot at the bottom depicts the distribution of LSNS scores with those considered socially isolated shown in red. I) Partial effects of LSNS scores on processing speed across the five imputations. The waffle plot indicated that the FDR-corrected q-values were significant in all five imputations.*

** < 0.05; ** < 0.01; *** < 0.001. Cognitive functions are measured in standard deviations. Grey areas indicate 95% confidence intervals.*

**Fig S14****
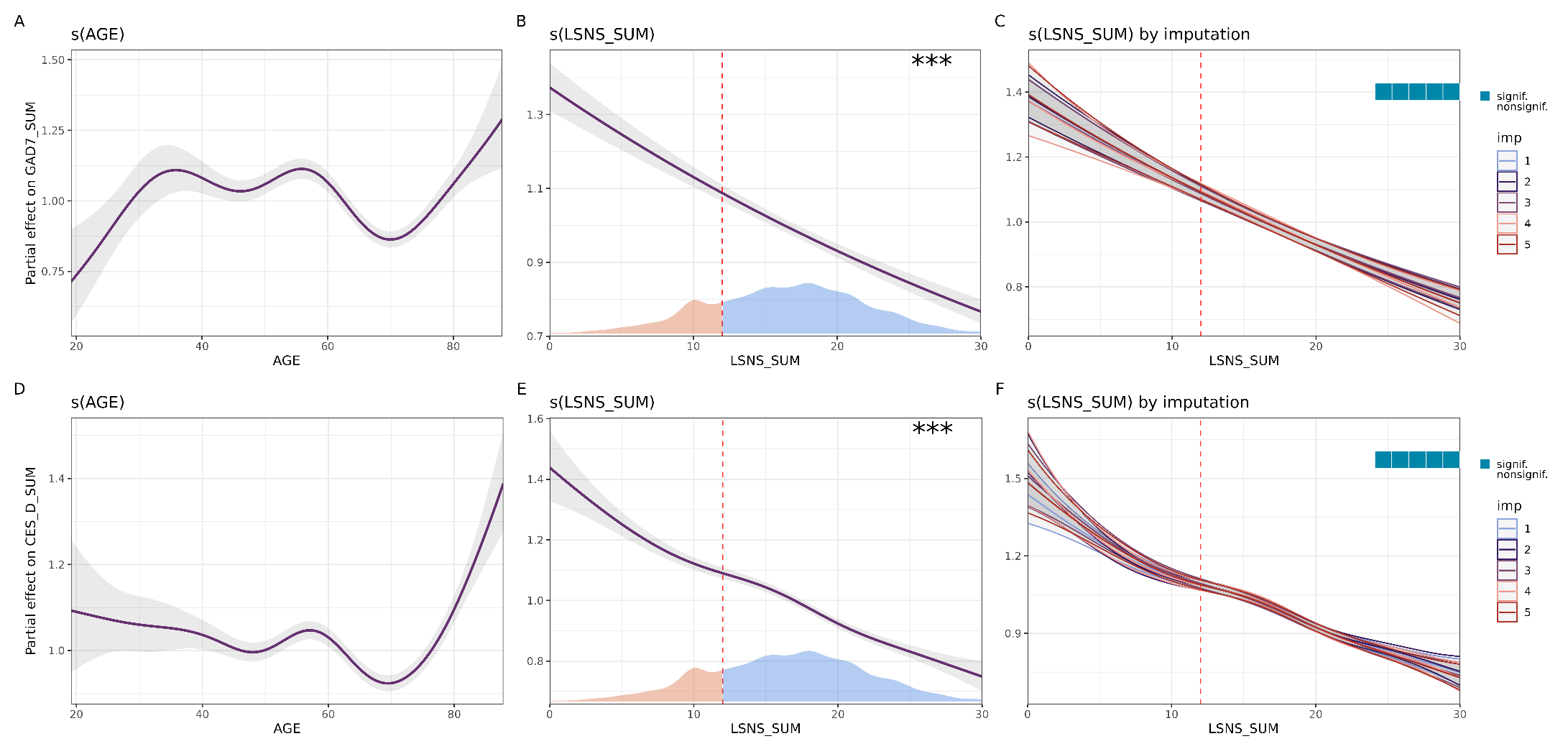
**

*Supplementary Fig. 14: Results of unweighted model 1. A) The partial effect of age on anxiety symptoms in the 1st imputed dataset. B) The partial effect of Lubben Social Network Scale (LSNS) scores on anxiety symptoms in the 1st imputed dataset. The dashed vertical line illustrates the standard LSNS cut-off. The density plot at the bottom depicts the distribution of LSNS scores with those considered socially isolated shown in red. C) Partial effects of LSNS scores on anxiety symptoms across the five imputations. The waffle plot indicated that the FDR-corrected q-values were significant in all five imputations. D) The partial effect of age on depressive symptoms in the 1st imputed dataset. E) The partial effect of Lubben Social Network Scale (LSNS) scores on depressive symptoms in the 1st imputed dataset. The dashed vertical line illustrates the standard LSNS cut-off. The density plot at the bottom depicts the distribution of LSNS scores with those considered socially isolated shown in red. F) Partial effects of LSNS scores on depressive symptoms across the five imputations. The waffle plot indicated that the FDR-corrected q-values were significant in all five imputations*

** < 0.05; ** < 0.01; *** < 0.001. GAD7 and CESD are measured in points on the respective questionnaire. Grey areas indicate 95% confidence intervals.*

**Fig S15**

**
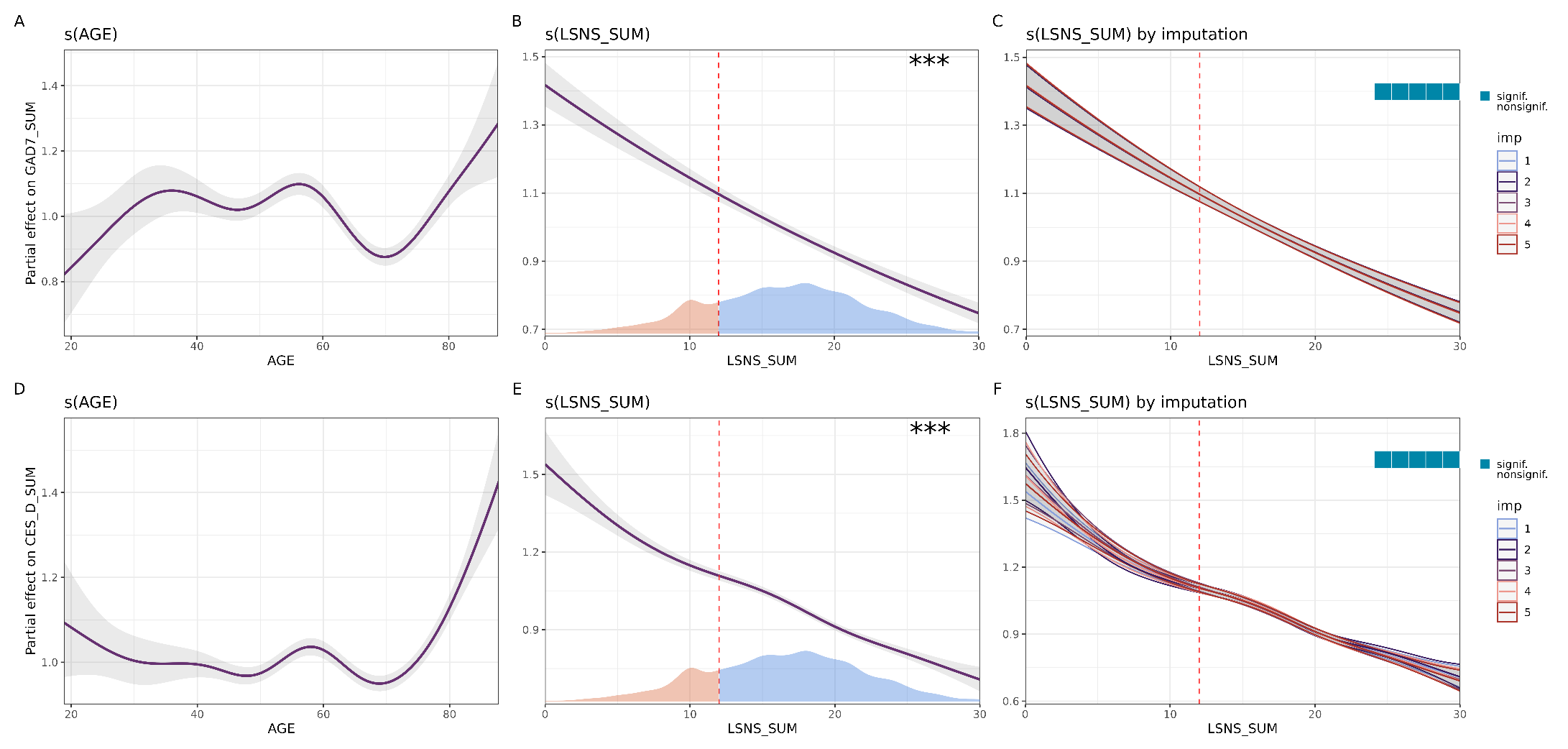
**

*Supplementary Fig. 15: Results of unweighted model 2. A) The partial effect of age on anxiety symptoms in the 1st imputed dataset. B) The partial effect of Lubben Social Network Scale (LSNS) scores on anxiety symptoms in the 1st imputed dataset. The dashed vertical line illustrates the standard LSNS cut-off. The density plot at the bottom depicts the distribution of LSNS scores with those considered socially isolated shown in red. C) Partial effects of LSNS scores on anxiety symptoms across the five imputations. The waffle plot indicated that the FDR-corrected q-values were significant in all five imputations. D) The partial effect of age on depressive symptoms in the 1st imputed dataset. E) The partial effect of Lubben Social Network Scale (LSNS) scores on depressive symptoms in the 1st imputed dataset. The dashed vertical line illustrates the standard LSNS cut-off. The density plot at the bottom depicts the distribution of LSNS scores with those considered socially isolated shown in red. F) Partial effects of LSNS scores on depressive symptoms across the five imputations. The waffle plot indicated that the FDR-corrected q-values were significant in all five imputations*

** < 0.05; ** < 0.01; *** < 0.001. GAD7 and CESD are measured in points on the respective questionnaire. Grey areas indicate 95% confidence intervals.*

**Fig S16**

**
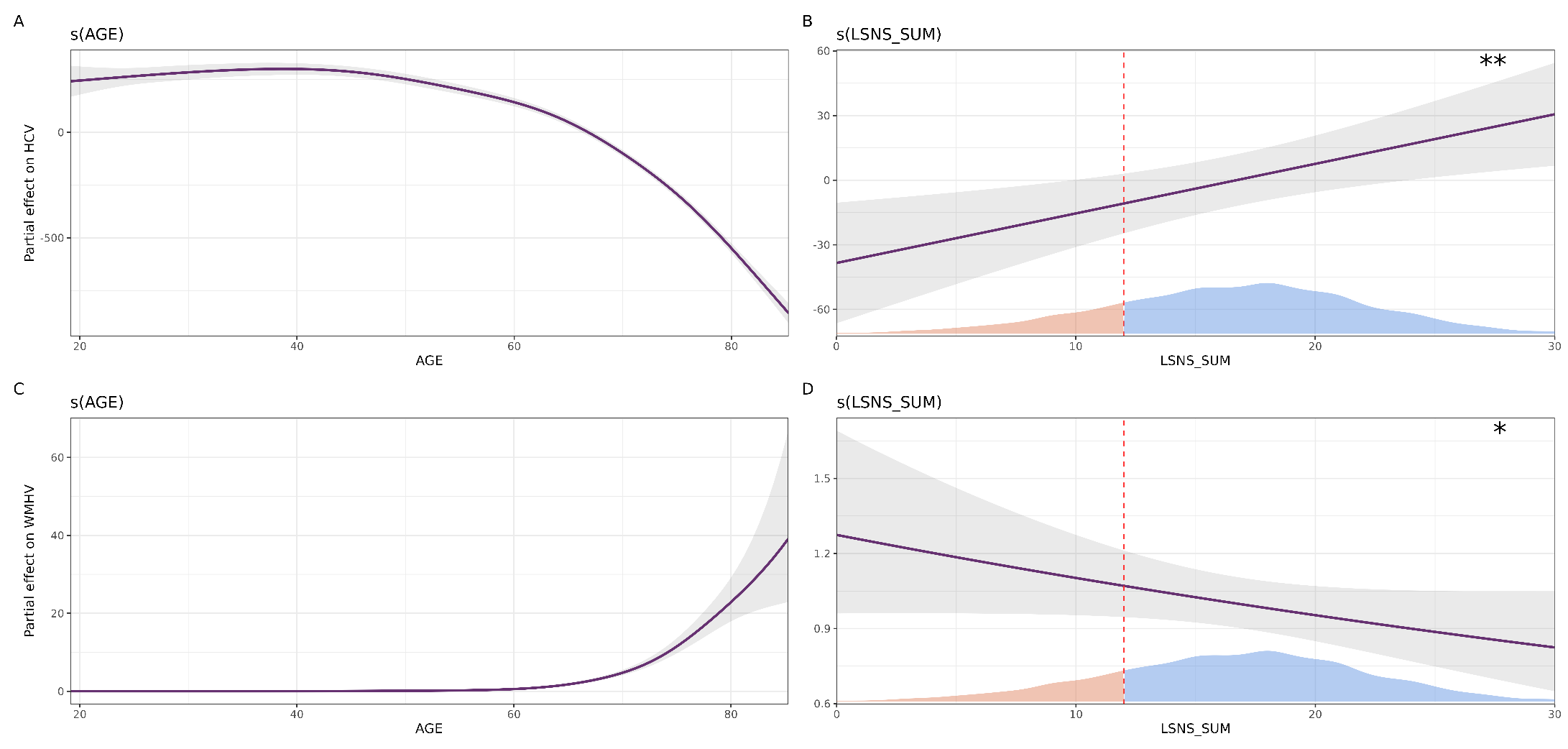
**

*Supplementary Fig. 16: Results of model 1 with only observed data. A) The partial effect of age on hippocampal volume (HCV). B) The partial effect of Lubben Social Network Scale (LSNS) scores on hippocampal volume (HCV). The dashed vertical line illustrates the standard LSNS cut-off. The density plot at the bottom depicts the distribution of LSNS scores with those considered socially isolated shown in red. C) The partial effect of age on white matter hyperintensity volume (WMHV) D) The partial effect of Lubben Social Network Scale (LSNS) scores on white matter hyperintensity volume. The dashed vertical line illustrates the standard LSNS cut-off. The density plot at the bottom depicts the distribution of LSNS scores with those considered socially isolated shown in red.*

** < 0.05; ** < 0.01; *** < 0.001. HCV and WMHV are measured in mm3. Grey areas indicate 95% confidence intervals.*

**Fig S17**


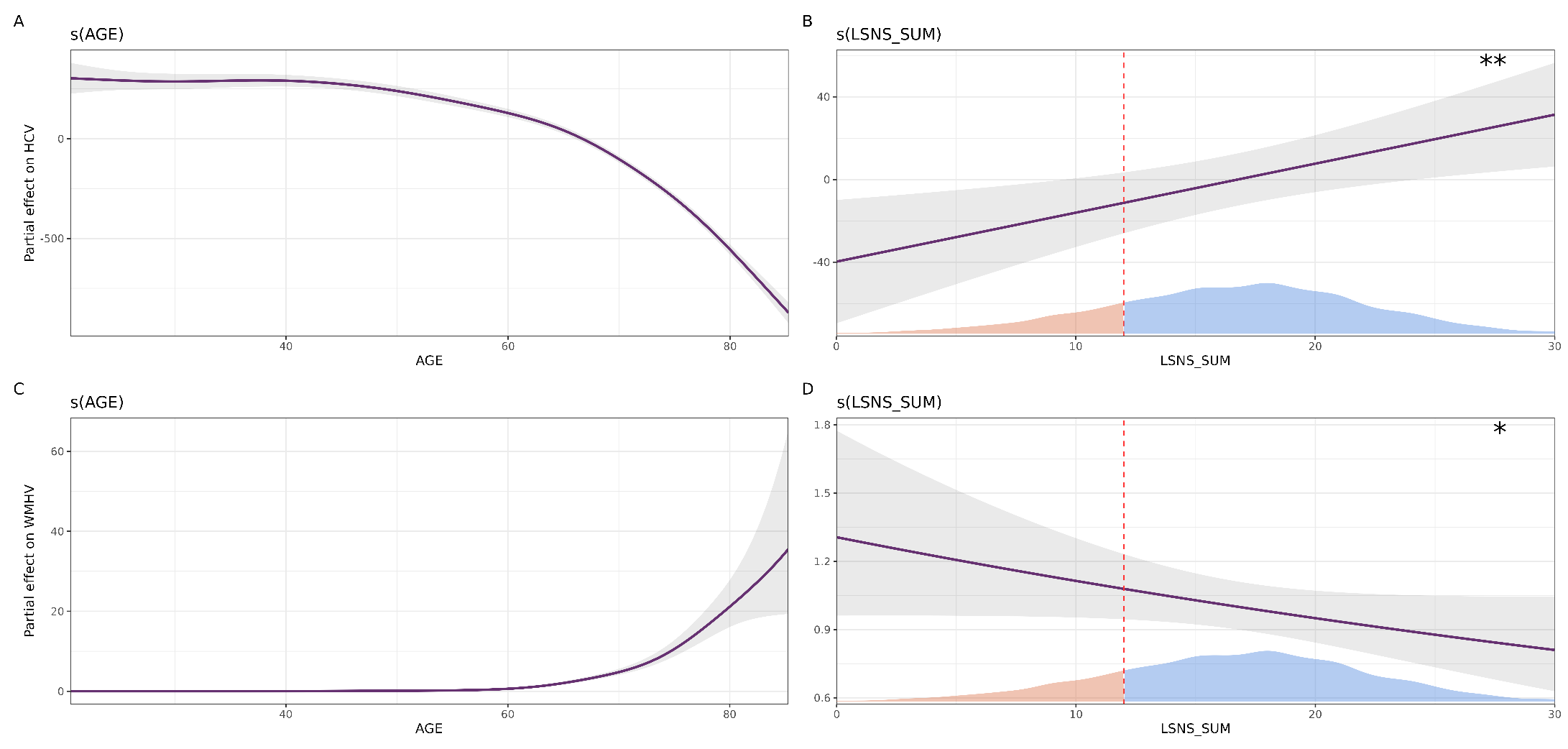


*Supplementary Fig. 17: Results of model 2 with only observed data. A) The partial effect of age on hippocampal volume (HCV). B) The partial effect of Lubben Social Network Scale (LSNS) scores on hippocampal volume (HCV). The dashed vertical line illustrates the standard LSNS cut-off. The density plot at the bottom depicts the distribution of LSNS scores with those considered socially isolated shown in red. C) The partial effect of age on white matter hyperintensity volume (WMHV) D) The partial effect of Lubben Social Network Scale (LSNS) scores on white matter hyperintensity volume. The dashed vertical line illustrates the standard LSNS cut-off. The density plot at the bottom depicts the distribution of LSNS scores with those considered socially isolated shown in red.*

** < 0.05; ** < 0.01; *** < 0.001. HCV and WMHV are measured in mm3. Grey areas indicate 95% confidence intervals.*

**Fig S18**


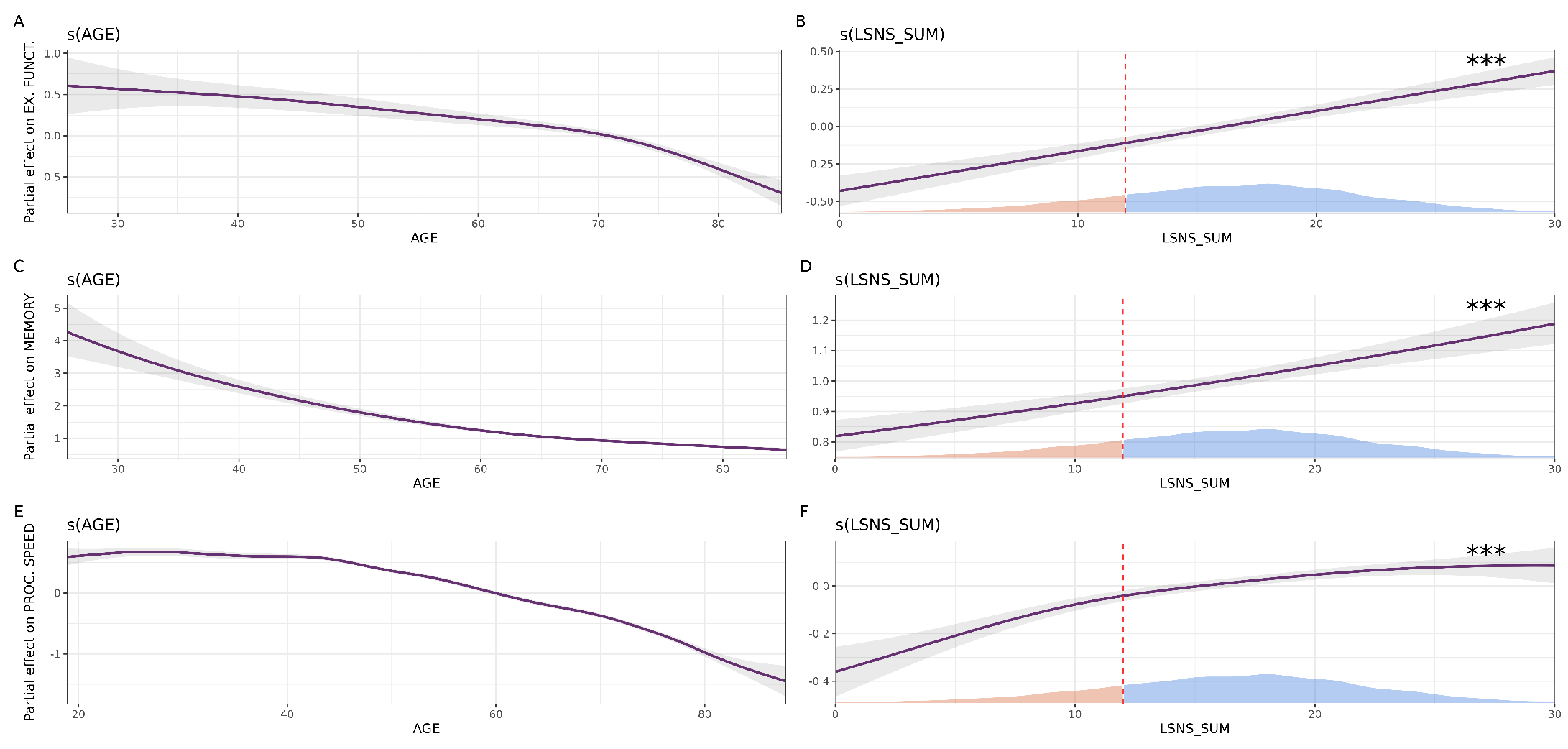


*Supplementary Fig. 18: Results of model 1 with only observed data. A) The partial effect of age on executive functions. B) The partial effect of Lubben Social Network Scale (LSNS) scores on executive functions. The dashed vertical line illustrates the standard LSNS cut-off. The density plot at the bottom depicts the distribution of LSNS scores with those considered socially isolated shown in red. C) The partial effect of age on memory. D) The partial effect of Lubben Social Network Scale (LSNS) scores on memory. The dashed vertical line illustrates the standard LSNS cut-off. The density plot at the bottom depicts the distribution of LSNS scores with those considered socially isolated shown in red. E) The partial effect of age on processing speed. F) The partial effect of Lubben Social Network Scale (LSNS) scores on processing speed. The dashed vertical line illustrates the standard LSNS cut-off. The density plot at the bottom depicts the distribution of LSNS scores with those considered socially isolated shown in red.*

** < 0.05; ** < 0.01; *** < 0.001. Cognitive functions are measured in standard deviations. Grey areas indicate 95% confidence intervals.*

**Fig S19**

**
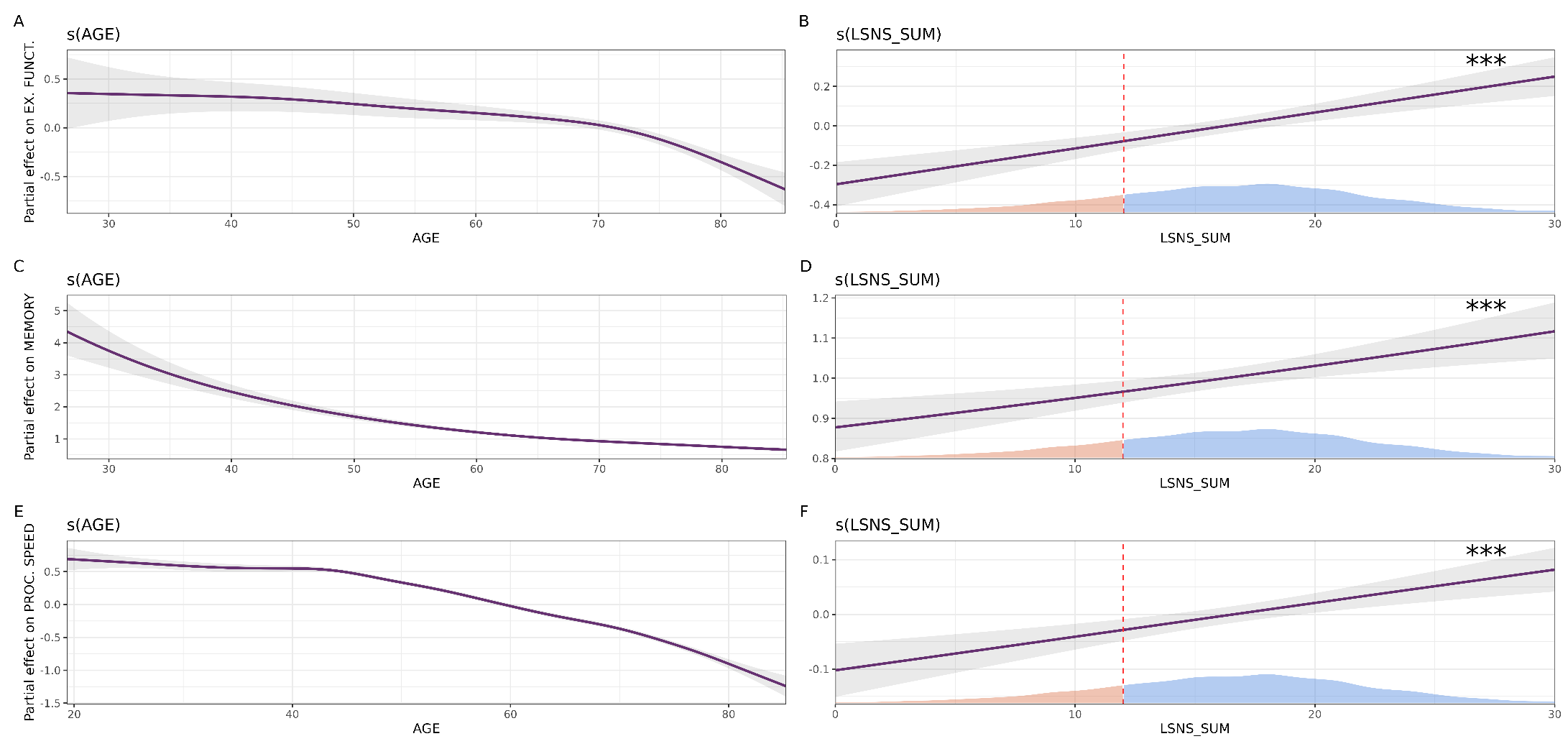
**

*Supplementary Fig. 19: Results of model 2 with only observed data. A) The partial effect of age on executive functions. B) The partial effect of Lubben Social Network Scale (LSNS) scores on executive functions. The dashed vertical line illustrates the standard LSNS cut-off. The density plot at the bottom depicts the distribution of LSNS scores with those considered socially isolated shown in red. C) The partial effect of age on memory. D) The partial effect of Lubben Social Network Scale (LSNS) scores on memory. The dashed vertical line illustrates the standard LSNS cut-off. The density plot at the bottom depicts the distribution of LSNS scores with those considered socially isolated shown in red. E) The partial effect of age on processing speed. F) The partial effect of Lubben Social Network Scale (LSNS) scores on processing speed. The dashed vertical line illustrates the standard LSNS cut-off. The density plot at the bottom depicts the distribution of LSNS scores with those considered socially isolated shown in red.*

** < 0.05; ** < 0.01; *** < 0.001. Cognitive functions are measured in standard deviations. Grey areas indicate 95% confidence intervals.*

**Fig S20**

**
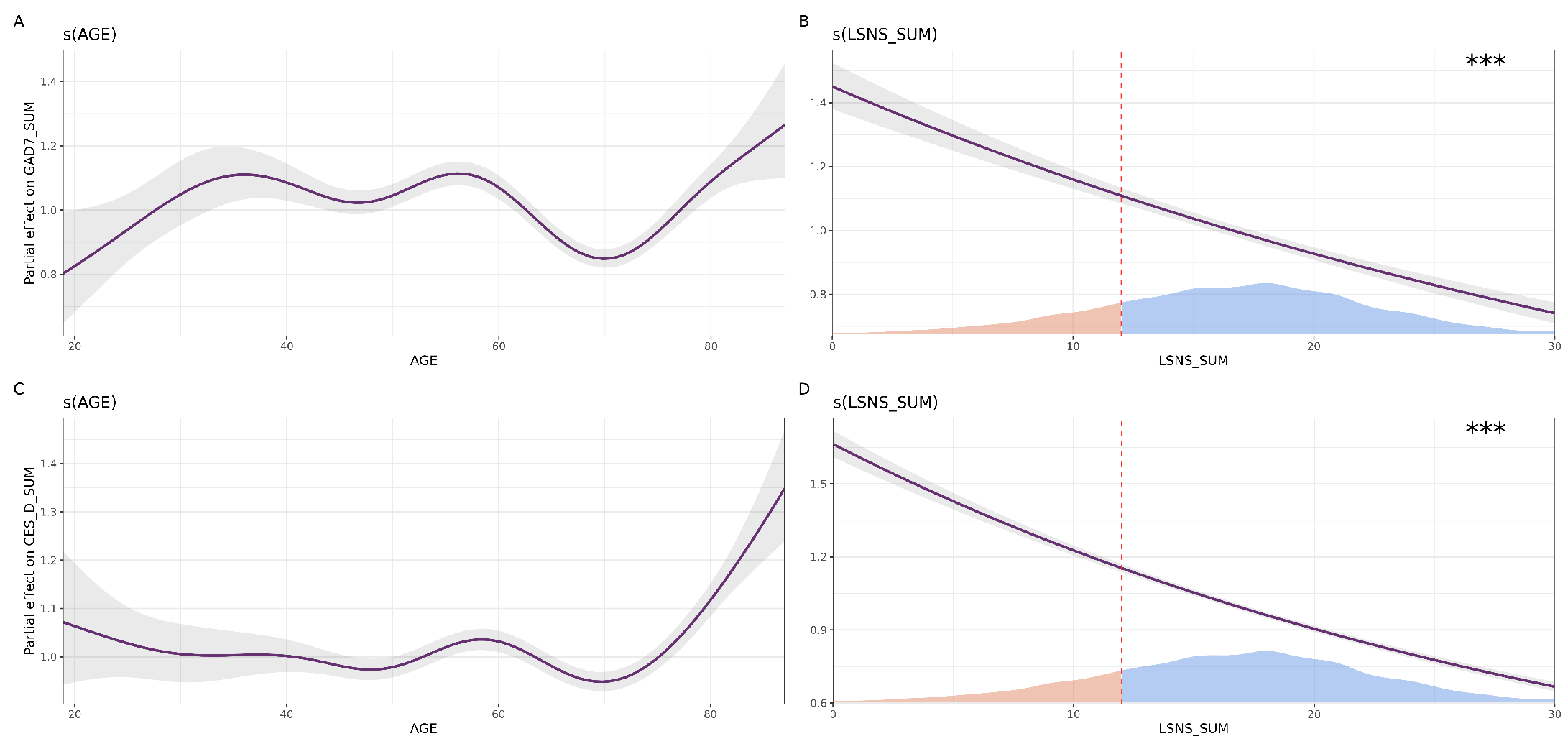
**

*Supplementary Fig. 20: Results of model 1 with only observed data. A) The partial effect of age on anxiety symptoms. B) The partial effect of Lubben Social Network Scale (LSNS) scores on anxiety symptoms. The dashed vertical line illustrates the standard LSNS cut-off. The density plot at the bottom depicts the distribution of LSNS scores with those considered socially isolated shown in red. C) The partial effect of age on depressive symptoms. D) The partial effect of Lubben Social Network Scale (LSNS) scores on depressive symptoms. The dashed vertical line illustrates the standard LSNS cut-off. The density plot at the bottom depicts the distribution of LSNS scores with those considered socially isolated shown in red.*

** < 0.05; ** < 0.01; *** < 0.001. GAD7 and CESD are measured in points on the respective questionnaire. Grey areas indicate 95% confidence intervals.*

**Fig S21**

**
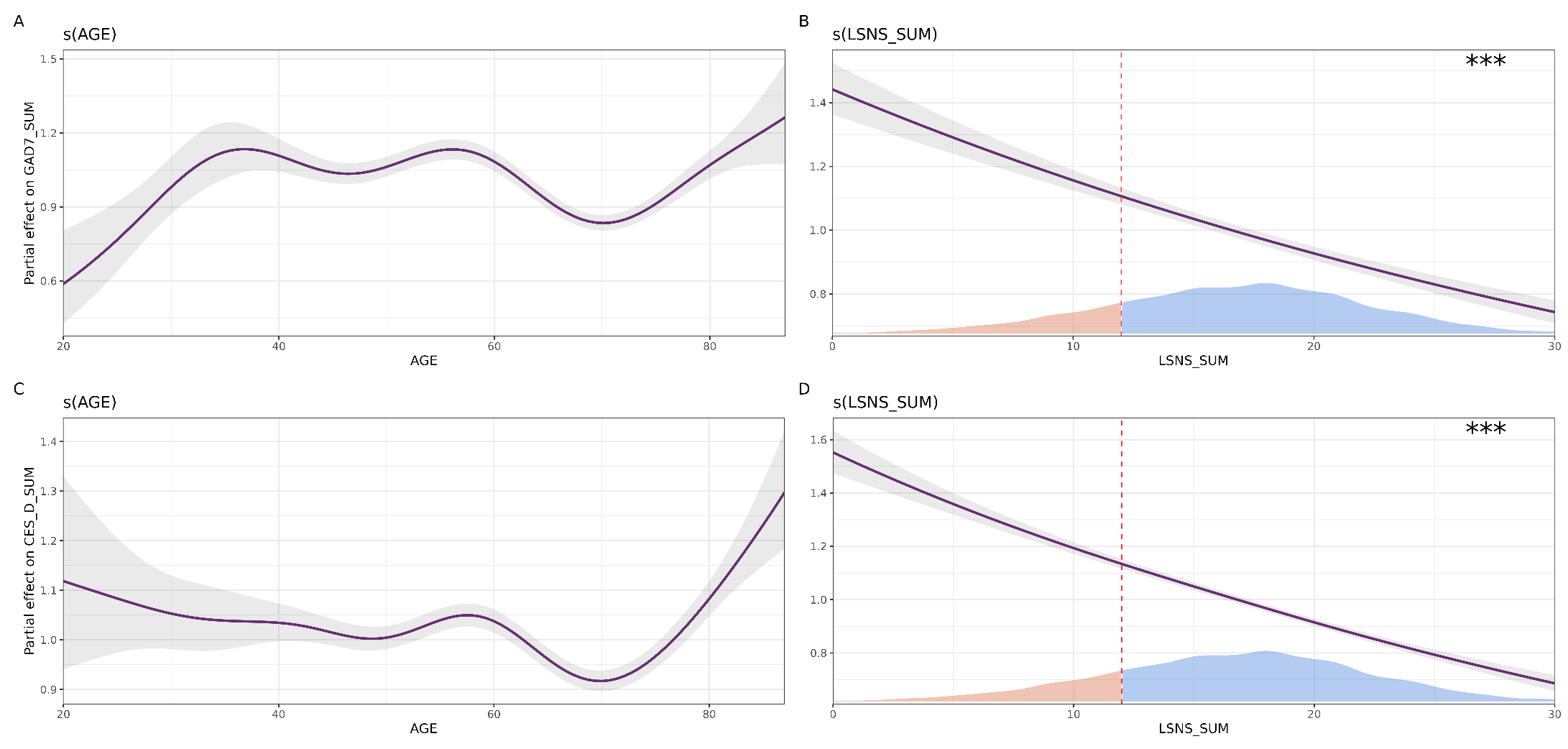
**

*Supplementary Fig. 21: Results of model 2 with only observed data. A) The partial effect of age on anxiety symptoms. B) The partial effect of Lubben Social Network Scale (LSNS) scores on anxiety symptoms. The dashed vertical line illustrates the standard LSNS cut-off. The density plot at the bottom depicts the distribution of LSNS scores with those considered socially isolated shown in red. C) The partial effect of age on depressive symptoms. D) The partial effect of Lubben Social Network Scale (LSNS) scores on depressive symptoms. The dashed vertical line illustrates the standard LSNS cut-off. The density plot at the bottom depicts the distribution of LSNS scores with those considered socially isolated shown in red.*

** < 0.05; ** < 0.01; *** < 0.001. GAD7 and CESD are measured in points on the respective questionnaire. Grey areas indicate 95% confidence intervals.*

**Fig S22**

**
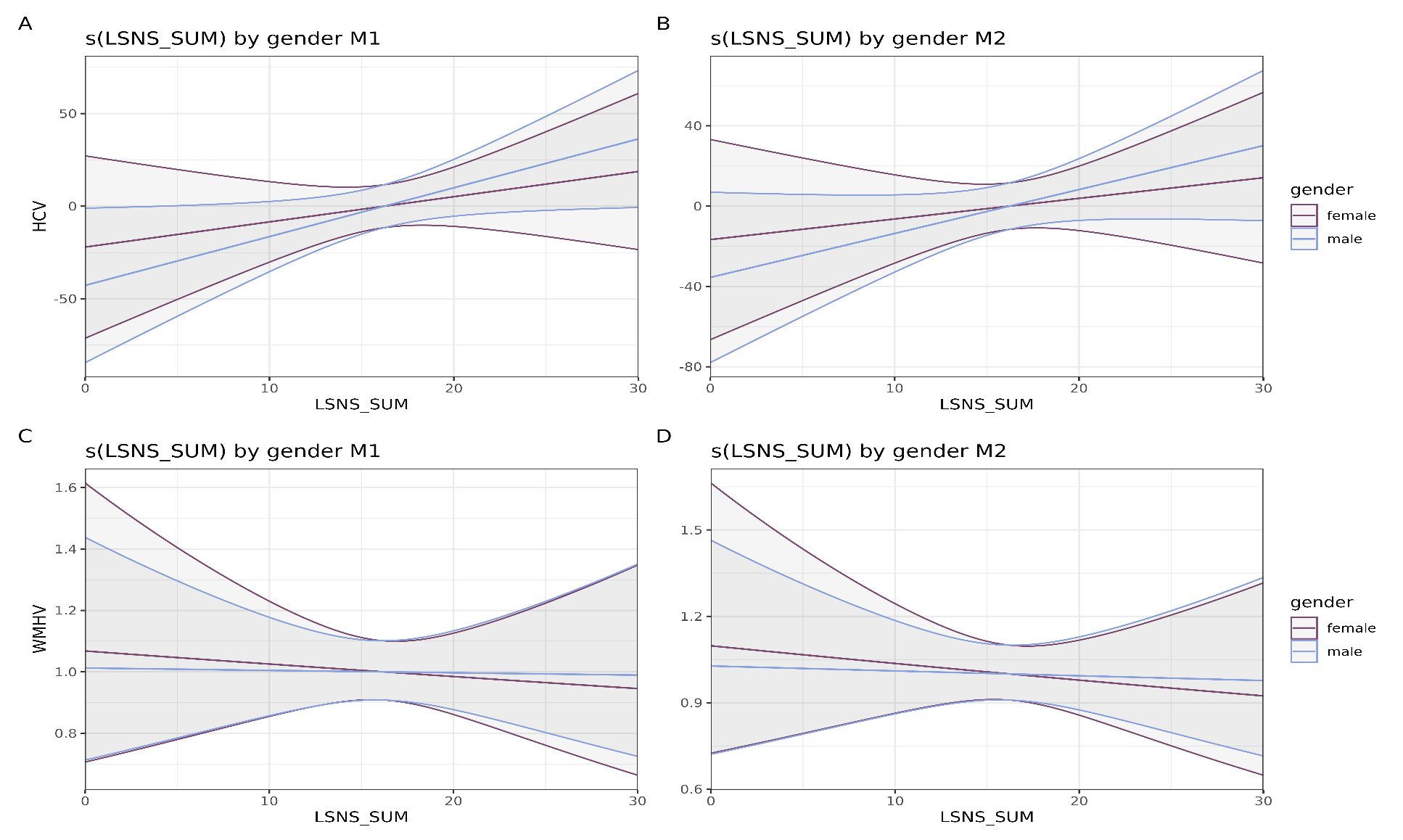
**

*Supplementary Fig. 22: A) The partial effect of Lubben Social Network Scale (LSNS) scores on hippocampal volume by gender in model 1. B) The partial effect of Lubben Social Network Scale (LSNS) scores on hippocampal volume by gender in model 2. C) The partial effect of Lubben Social Network Scale (LSNS) scores on white matter hyperintensity volume (WMHV) by gender in model 1. D) The partial effect of Lubben Social Network Scale (LSNS) scores on white matter hyperintensity volume by gender in model 2. HCV and WMHV are measured in mm3. Grey areas indicate 95% confidence intervals.*

**Fig S23**

**
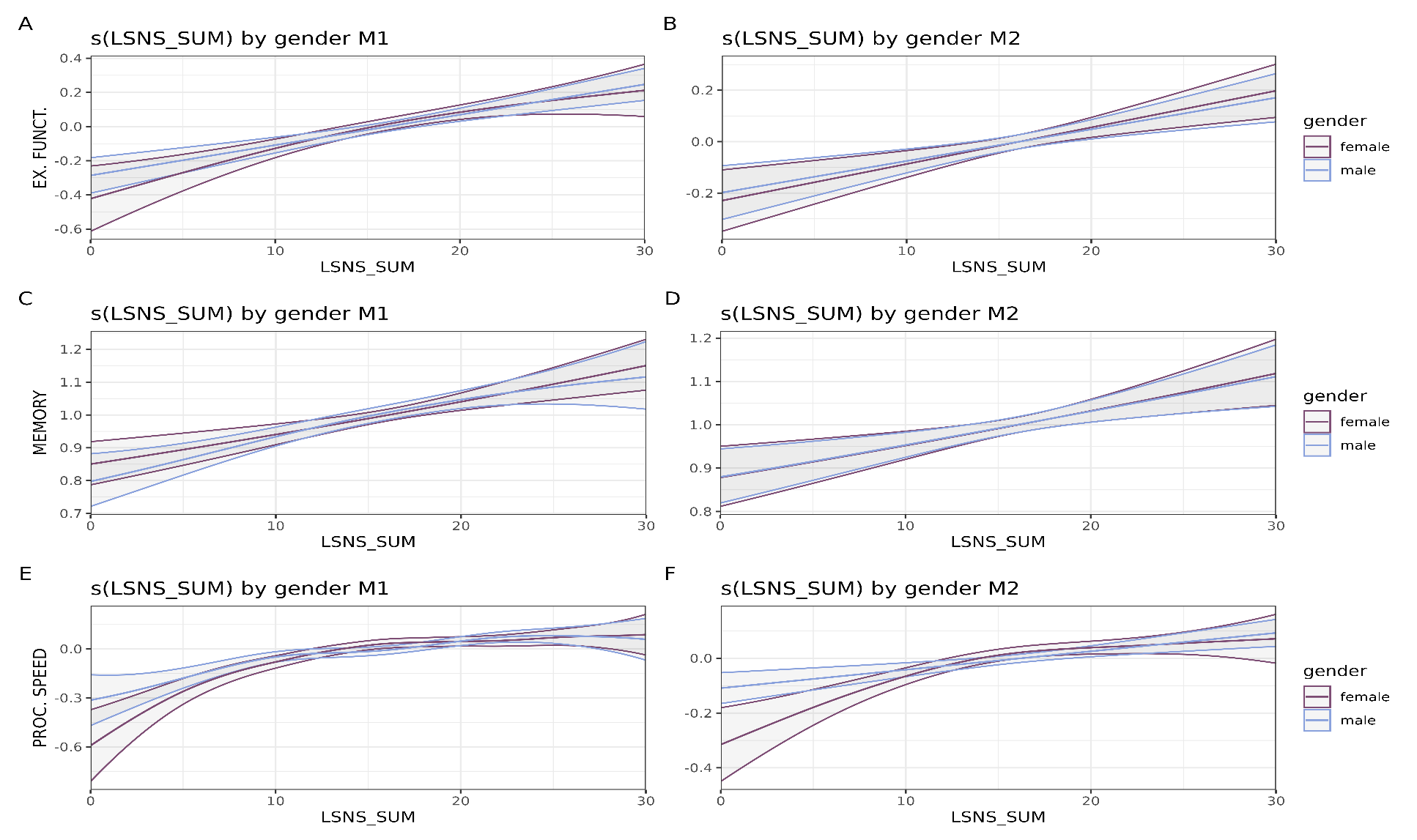
**

*Supplementary Fig. 23: A) The partial effect of Lubben Social Network Scale (LSNS) scores on executive functions by gender in model 1. B) The partial effect of Lubben Social Network Scale (LSNS) scores on executive functions by gender in model 2. C) The partial effect of Lubben Social Network Scale (LSNS) scores on memory by gender in model 1. D) The partial effect of Lubben Social Network Scale (LSNS) scores on memory by gender in model 2. E) The partial effect of Lubben Social Network Scale (LSNS) scores on processing speed by gender in model 1. F) The partial effect of Lubben Social Network Scale (LSNS) scores on processing speed by gender in model 2. Cognitive functions are measured in standard deviations. Grey areas indicate 95% confidence intervals.*

**Fig S24**

**
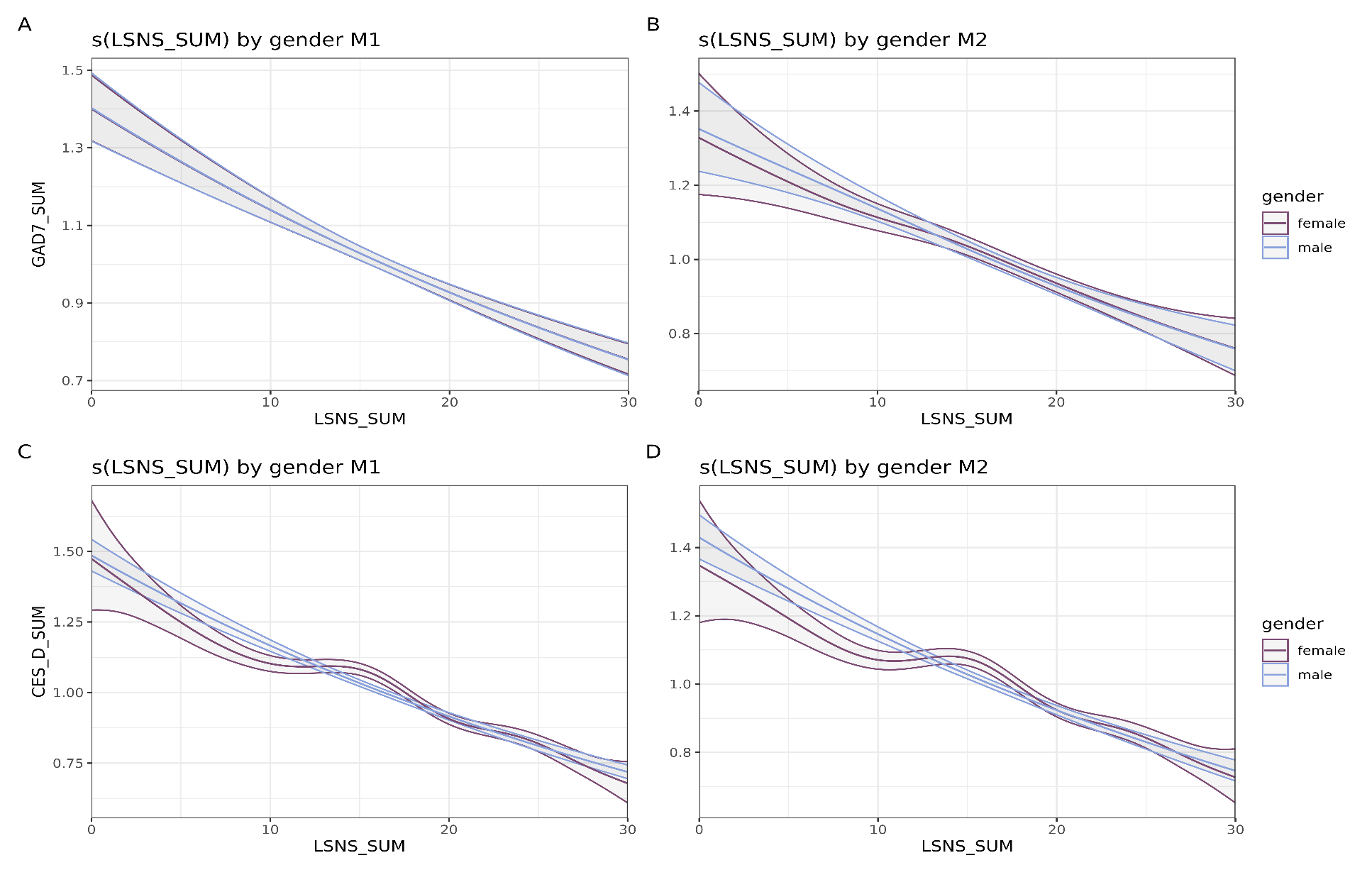
**

*Supplementary Fig. 24: A) The partial effect of Lubben Social Network Scale (LSNS) scores on anxiety symptoms by gender in model 1. B) The partial effect of Lubben Social Network Scale (LSNS) scores on anxiety symptoms by gender in model 2. C) The partial effect of Lubben Social Network Scale (LSNS) scores on depressive symptoms by gender in model 1. D) The partial effect of Lubben Social Network Scale (LSNS) scores on depressive symptoms by gender in model 2. GAD7 and CESD are measured in points on the respective questionnaire. Grey areas indicate 95% confidence intervals.*

**Fig S25**


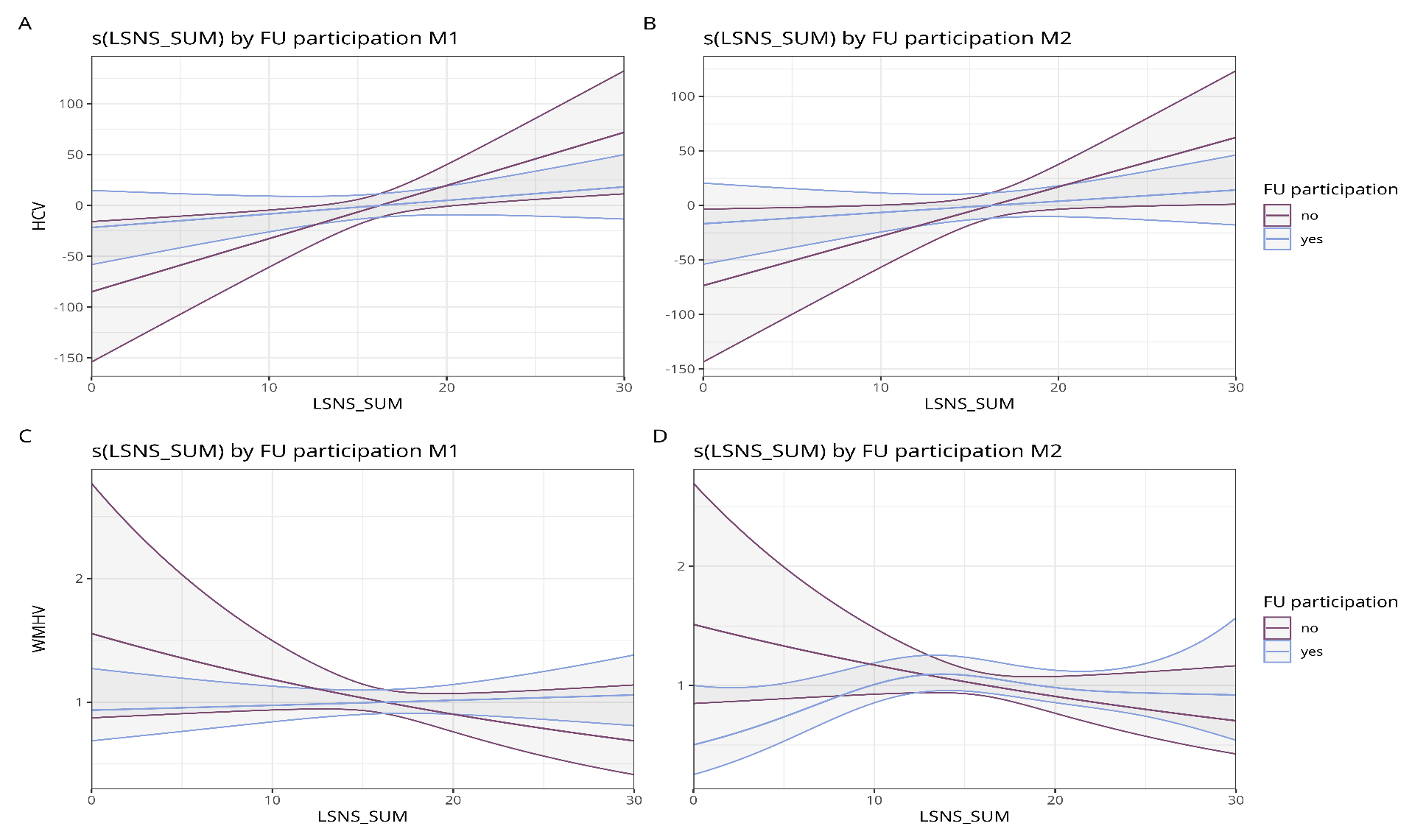


*Supplementary Fig. 25: A) The partial effect of Lubben Social Network Scale (LSNS) scores on hippocampal volume by follow-up (FU) participation in model 1. B) The partial effect of Lubben Social Network Scale (LSNS) scores on hippocampal volume by follow-up (FU) participation in model 2. C) The partial effect of Lubben Social Network Scale (LSNS) scores on white matter hyperintensity volume (WMHV) by FU participation in model 1. D) The partial effect of Lubben Social Network Scale (LSNS) scores on white matter hyperintensity volume by FU participation in model 2. HCV and WMHV are measured in mm3. Grey areas indicate 95% confidence intervals.*

**Fig S26**


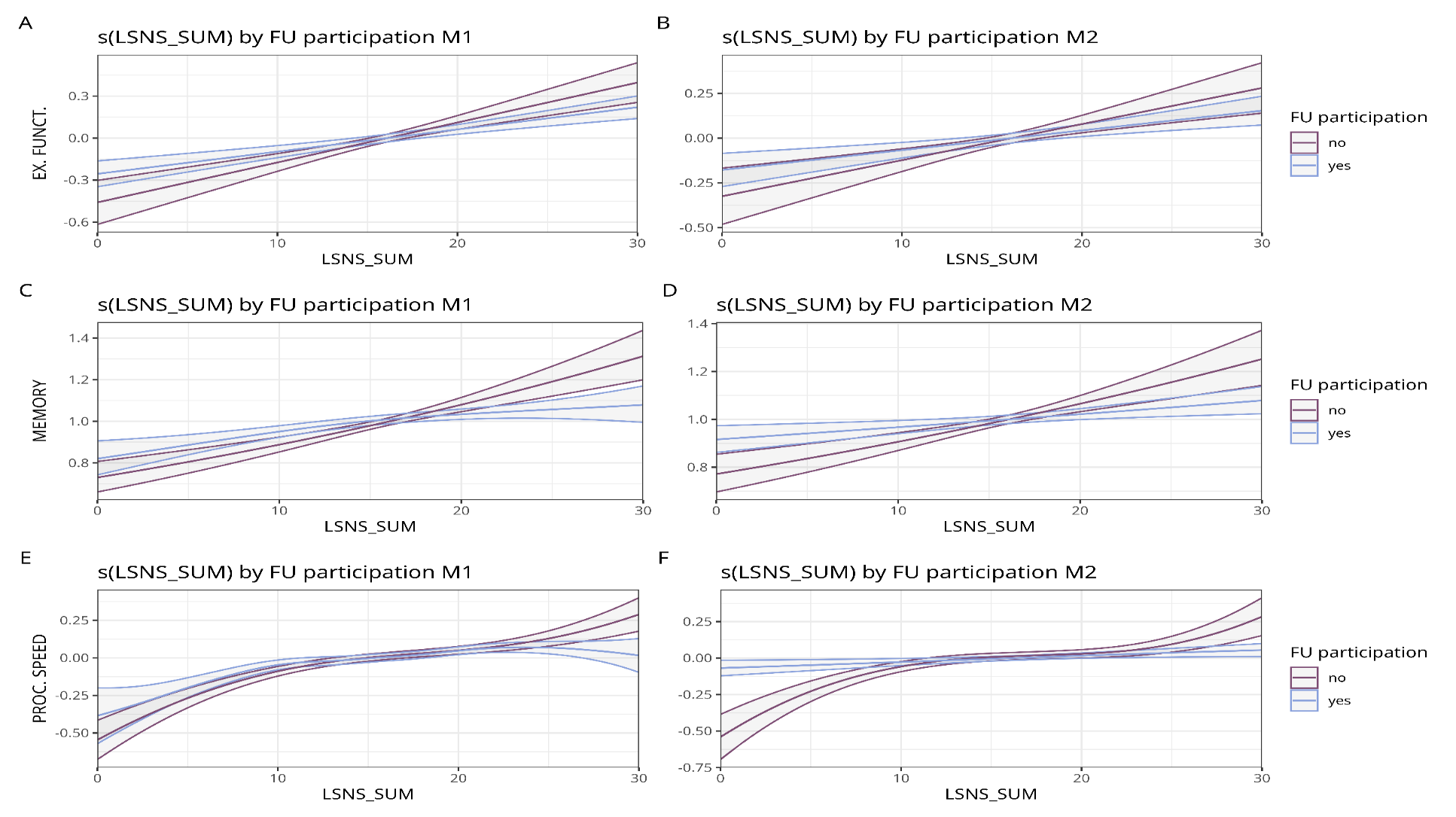


*Supplementary Fig. 26: A) The partial effect of Lubben Social Network Scale (LSNS) scores on executive functions by follow-up (FU) participation in model 1. B) The partial effect of Lubben Social Network Scale (LSNS) scores on executive functions by FU participation in model 2. C) The partial effect of Lubben Social Network Scale (LSNS) scores on memory by FU participation in model 1. D) The partial effect of Lubben Social Network Scale (LSNS) scores on memory by FU participation in model 2. E) The partial effect of Lubben Social Network Scale (LSNS) scores on processing speed by FU participation in model 1. F) The partial effect of Lubben Social Network Scale (LSNS) scores on processing speed by FU participation in model 2. Cognitive functions are measured in standard deviations. Grey areas indicate 95% confidence intervals.*

**Fig S27**


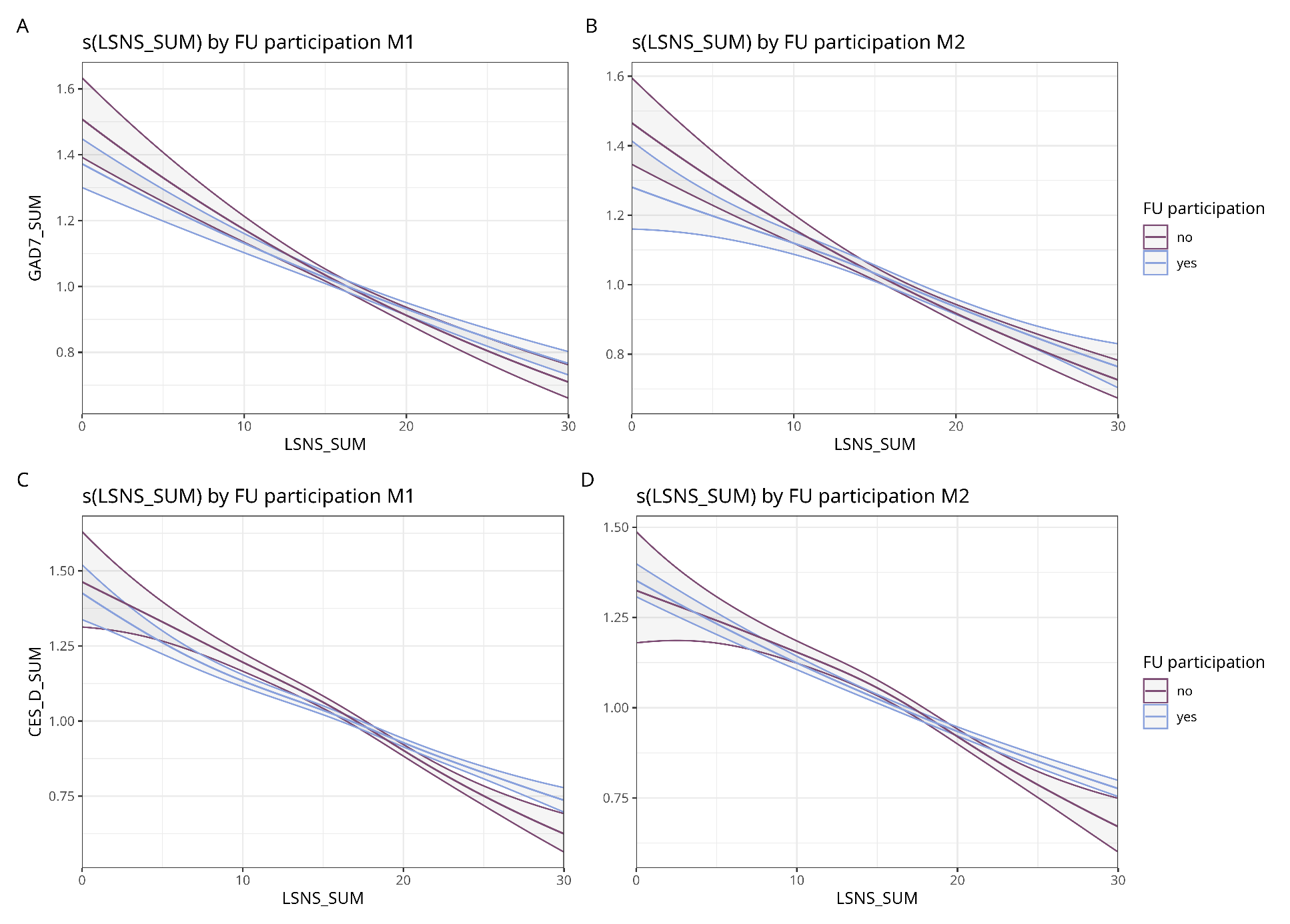


*Supplementary Fig. 27: A) The partial effect of Lubben Social Network Scale (LSNS) scores on anxiety symptoms by follow-up (FU) participation in model 1. B) The partial effect of Lubben Social Network Scale (LSNS) scores on anxiety symptoms by FU participation in model 2. C) The partial effect of Lubben Social Network Scale (LSNS) scores on depressive symptoms by FU participation in model 1. D) The partial effect of Lubben Social Network Scale (LSNS) scores on depressive symptoms by FU participation in model 2. GAD7 and CESD are measured in points on the respective questionnaire. Grey areas indicate 95% confidence intervals.*

**Fig S28**


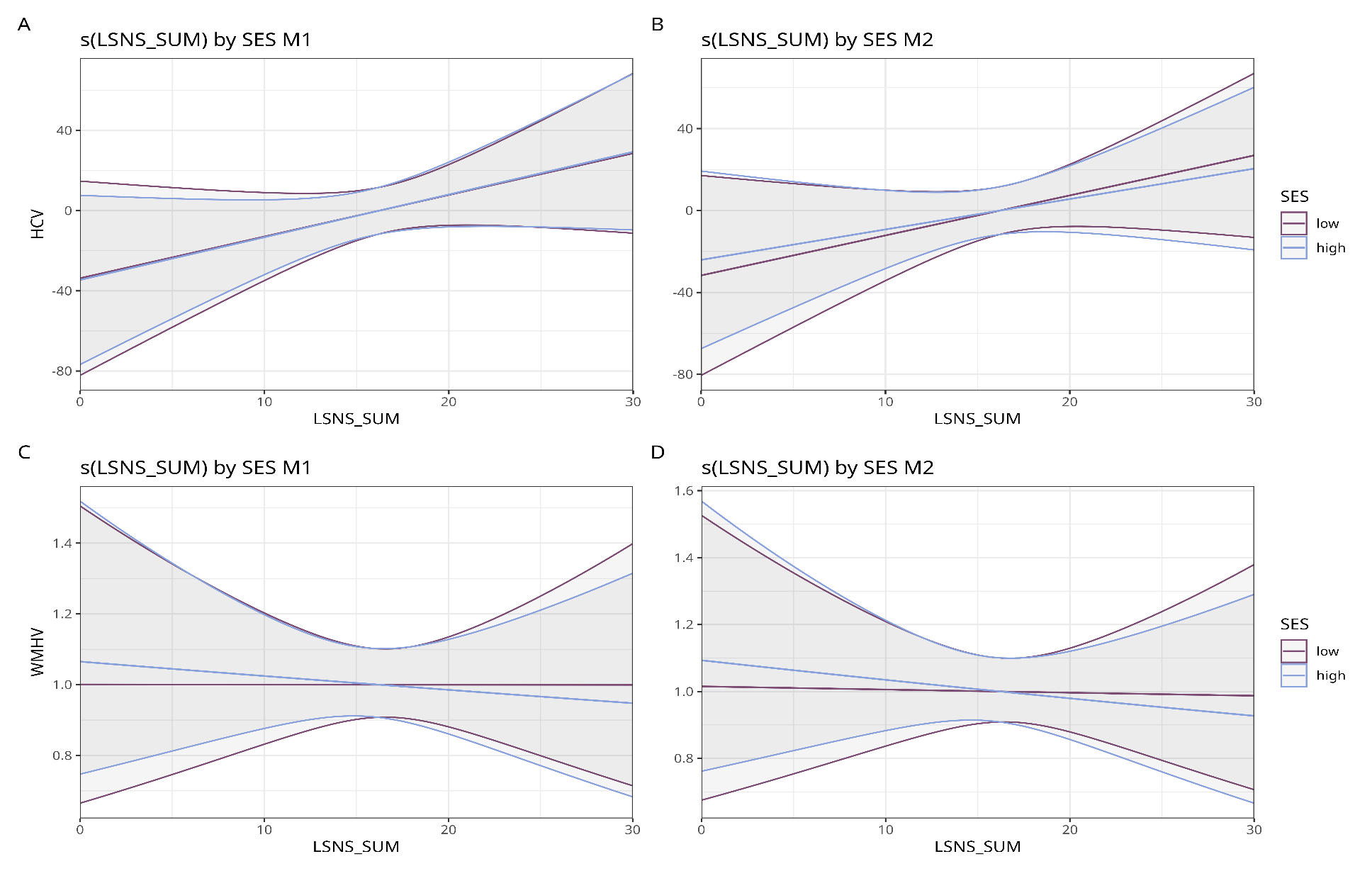


*Supplementary Fig. 28: A) The partial effect of Lubben Social Network Scale (LSNS) scores on hippocampal volume by socioeconomic status (SES) median split in model 1. B) The partial effect of Lubben Social Network Scale (LSNS) scores on hippocampal volume by SES median split in model 2. C) The partial effect of Lubben Social Network Scale (LSNS) scores on white matter hyperintensity volume (WMHV) by SES median split in model 1. D) The partial effect of Lubben Social Network Scale (LSNS) scores on white matter hyperintensity volume by SES median split in model 2. HCV and WMHV are measured in mm3. Grey areas indicate 95% confidence intervals.*

**Fig S29**

**
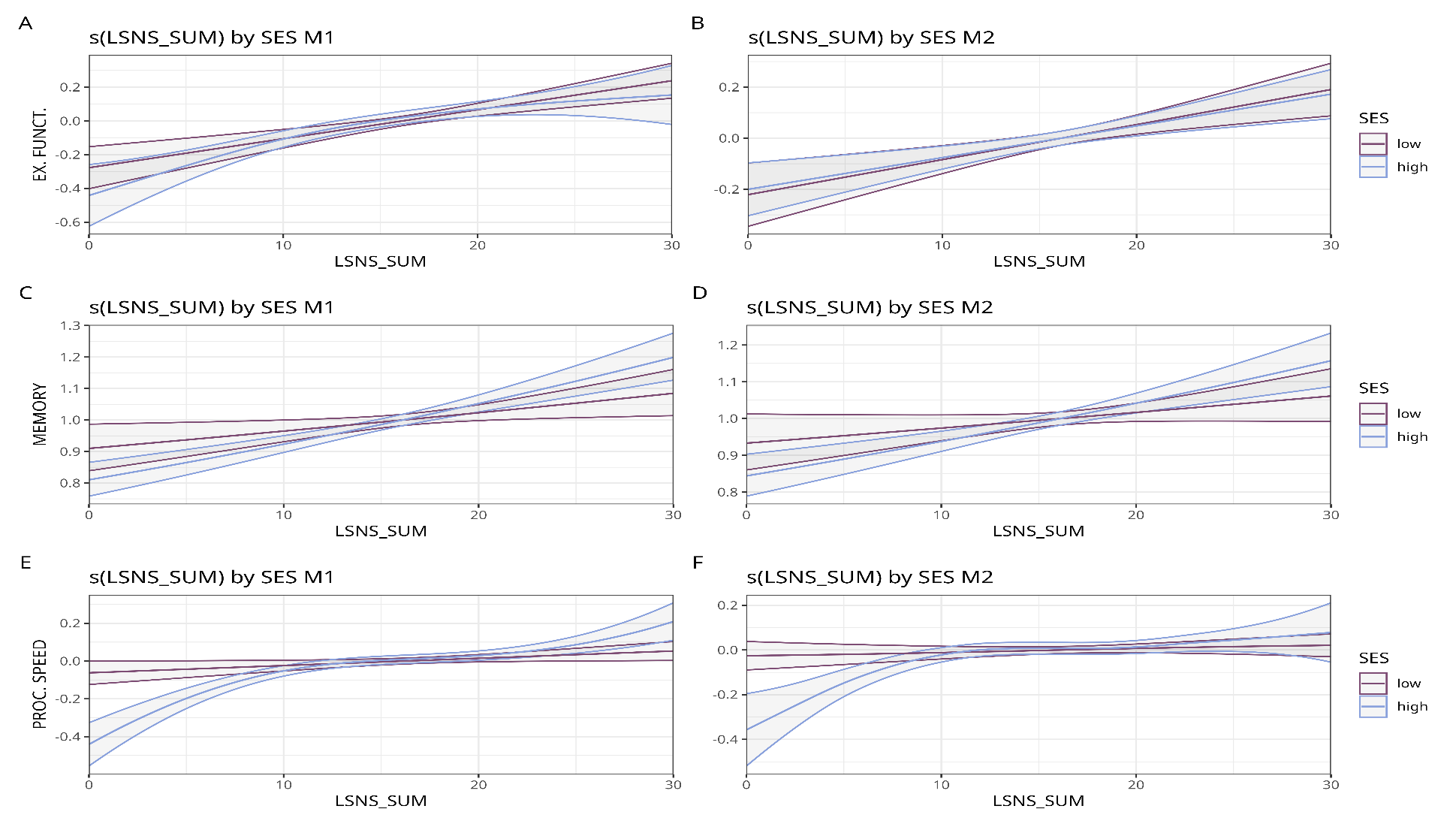
**

*Supplementary Fig. 29: A) The partial effect of Lubben Social Network Scale (LSNS) scores on executive functions by socioeconomic status (SES) median split in model 1. B) The partial effect of Lubben Social Network Scale (LSNS) scores on executive functions by SES median split in model 2. C) The partial effect of Lubben Social Network Scale (LSNS) scores on memory by SES median split in model 1. D) The partial effect of Lubben Social Network Scale (LSNS) scores on memory by SES median split in model 2. E) The partial effect of Lubben Social Network Scale (LSNS) scores on processing speed by SES median split in model 1. F) The partial effect of Lubben Social Network Scale (LSNS) scores on processing speed by SES median split in model 2. Cognitive functions are measured in standard deviations. Grey areas indicate 95% confidence intervals.*

**Fig S30**

**
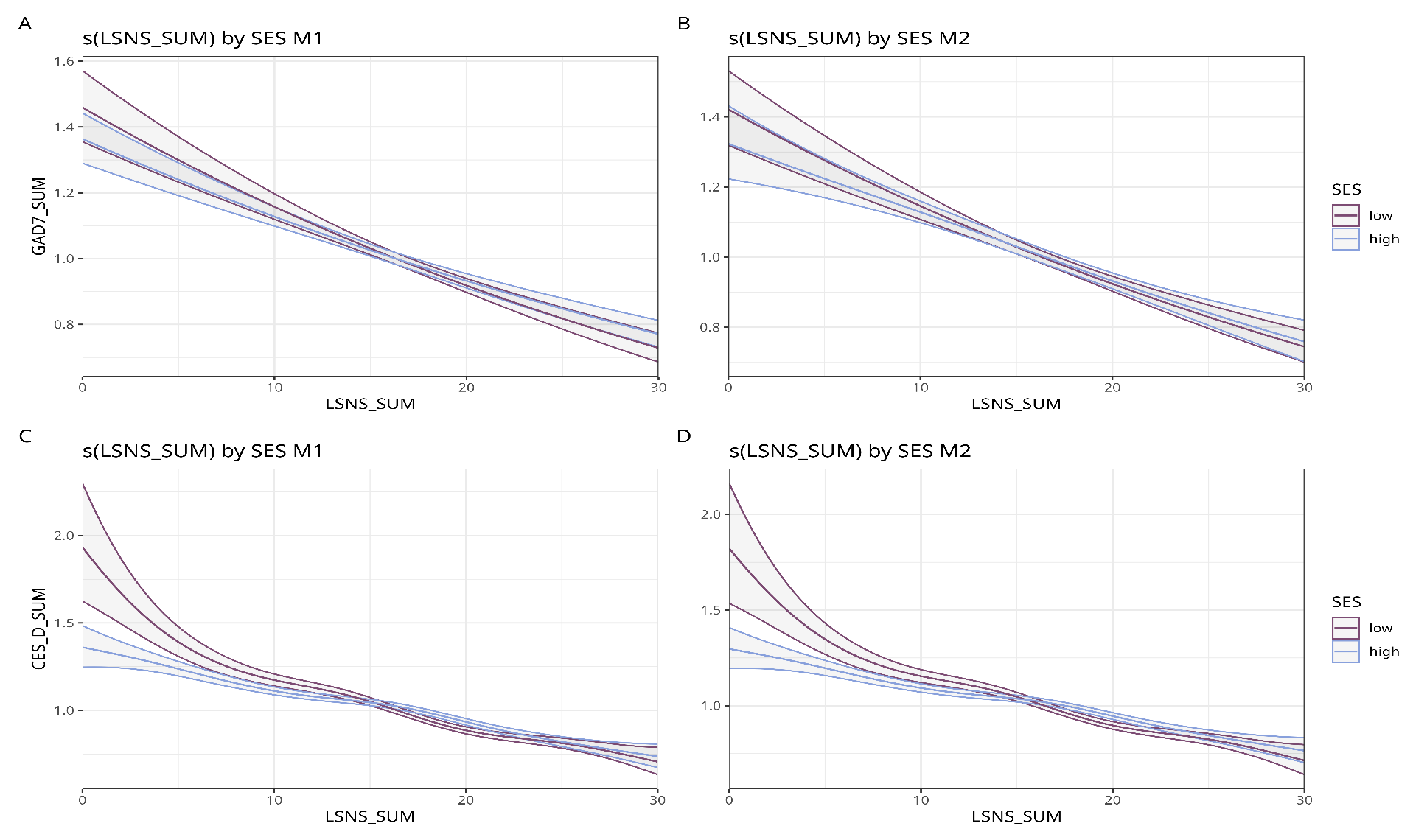
**

*Supplementary Fig. 30: A) The partial effect of Lubben Social Network Scale (LSNS) scores on anxiety symptoms by socioeconomic status (SES) median split in model 1. B) The partial effect of Lubben Social Network Scale (LSNS) scores on anxiety symptoms by SES median split in model 2. C) The partial effect of Lubben Social Network Scale (LSNS) scores on depressive symptoms by SES median split in model 1. D) The partial effect of Lubben Social Network Scale (LSNS) scores on depressive symptoms by SES median split in model 2. GAD7 and CESD are measured in points on the respective questionnaire. Grey areas indicate 95% confidence intervals.*

**Fig S31**

**
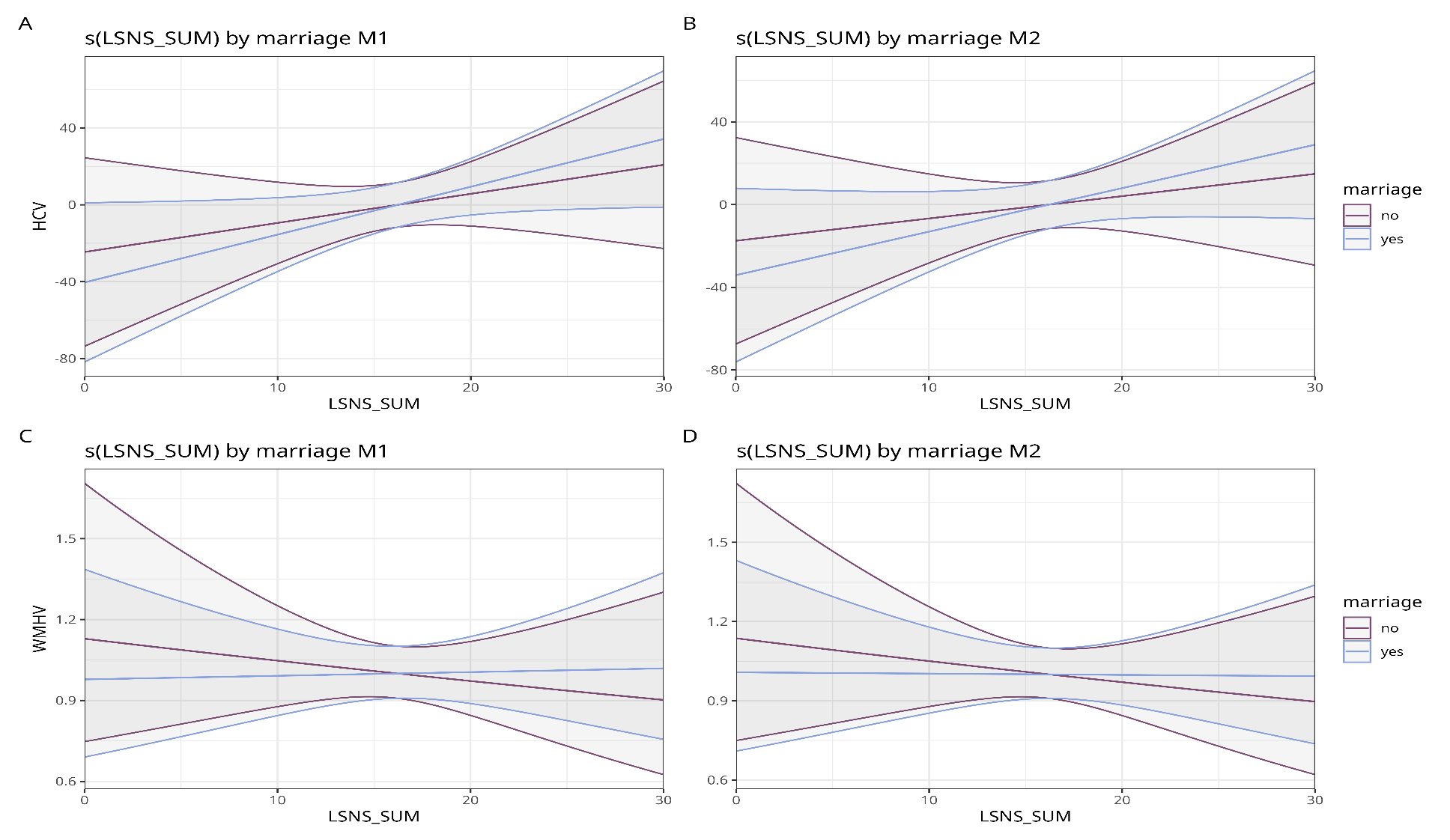
**

*Supplementary Fig. 31: A) The partial effect of Lubben Social Network Scale (LSNS) scores on hippocampal volume by marriage status in model 1. B) The partial effect of Lubben Social Network Scale (LSNS) scores on hippocampal volume by marriage status in model 2. C) The partial effect of Lubben Social Network Scale (LSNS) scores on white matter hyperintensity volume (WMHV) by marriage status in model 1. D) The partial effect of Lubben Social Network Scale (LSNS) scores on white matter hyperintensity volume by marriage status in model 2. HCV and WMHV are measured in mm3. Grey areas indicate 95% confidence intervals.*

**Fig S32**

**
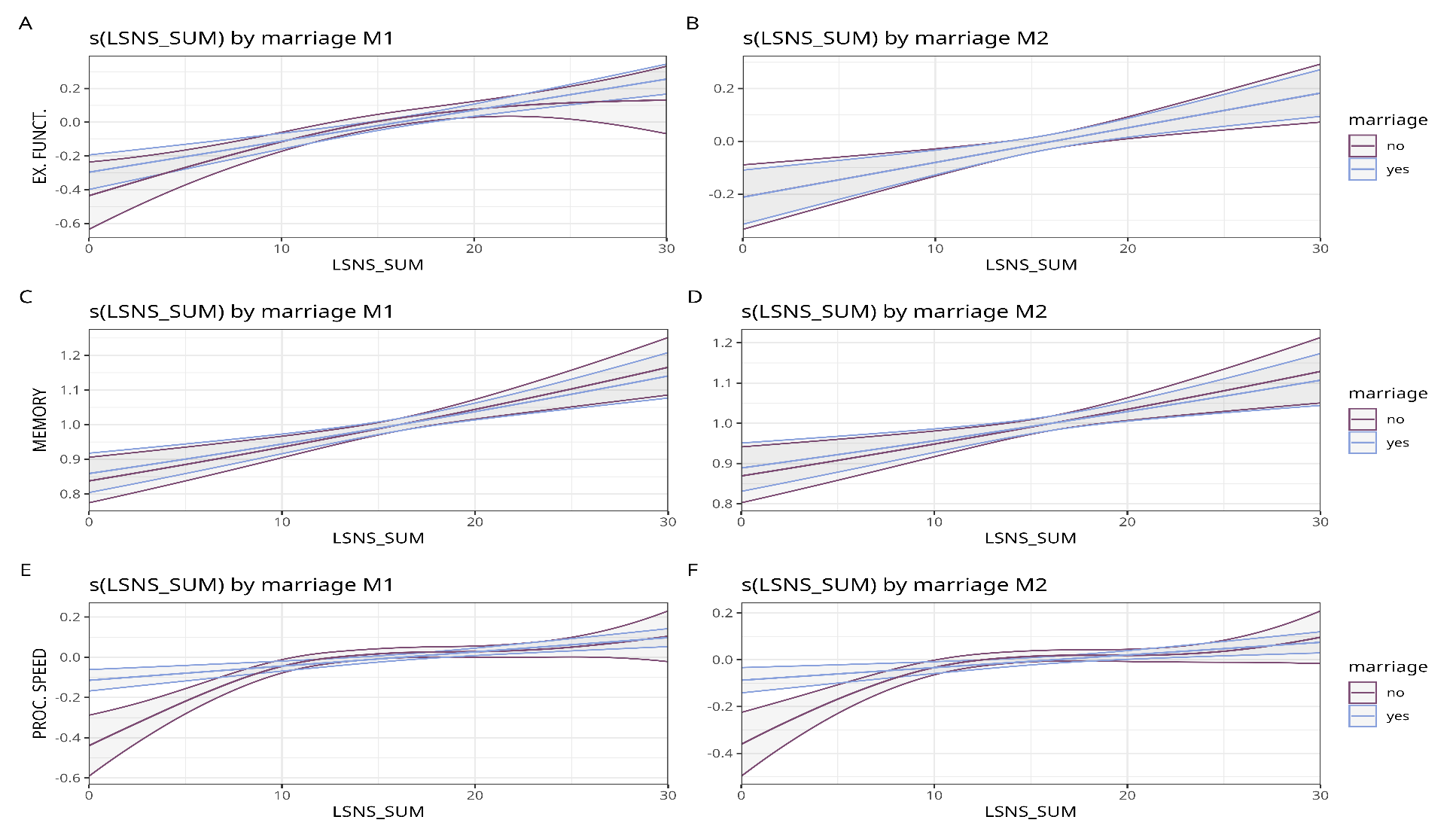
**

*Supplementary Fig. 32: A) The partial effect of Lubben Social Network Scale (LSNS) scores on executive functions by marriage status in model 1. B) The partial effect of Lubben Social Network Scale (LSNS) scores on executive functions by marriage status in model 2. C) The partial effect of Lubben Social Network Scale (LSNS) scores on memory by marriage status in model 1. D) The partial effect of Lubben Social Network Scale (LSNS) scores on memory by marriage status in model 2. E) The partial effect of Lubben Social Network Scale (LSNS) scores on processing speed by marriage status in model 1. F) The partial effect of Lubben Social Network Scale (LSNS) scores on processing speed by marriage status in model 2. Cognitive functions are measured in standard deviations. Grey areas indicate 95% confidence intervals.*

**Fig S33**

**
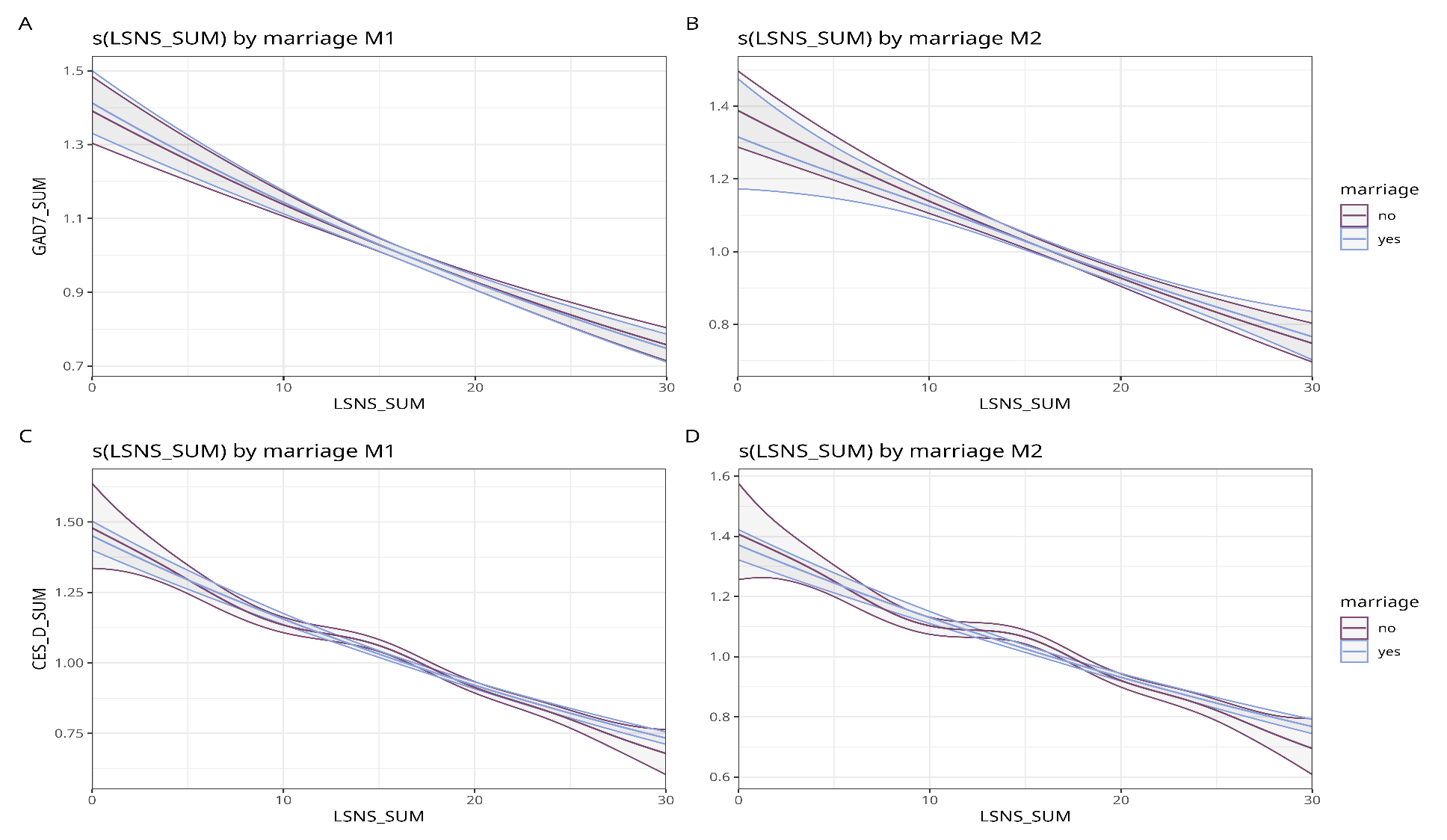
**

*Supplementary Fig. 33: A) The partial effect of Lubben Social Network Scale (LSNS) scores on anxiety symptoms by marriage status in model 1. B) The partial effect of Lubben Social Network Scale (LSNS) scores on anxiety symptoms by marriage status in model 2. C) The partial effect of Lubben Social Network Scale (LSNS) scores on depressive symptoms by marriage status in model 1. D) The partial effect of Lubben Social Network Scale (LSNS) scores on depressive symptoms by marriage status in model 2. GAD7 and CESD are measured in points on the respective questionnaire. Grey areas indicate 95% confidence intervals.*

**Fig S34**

**
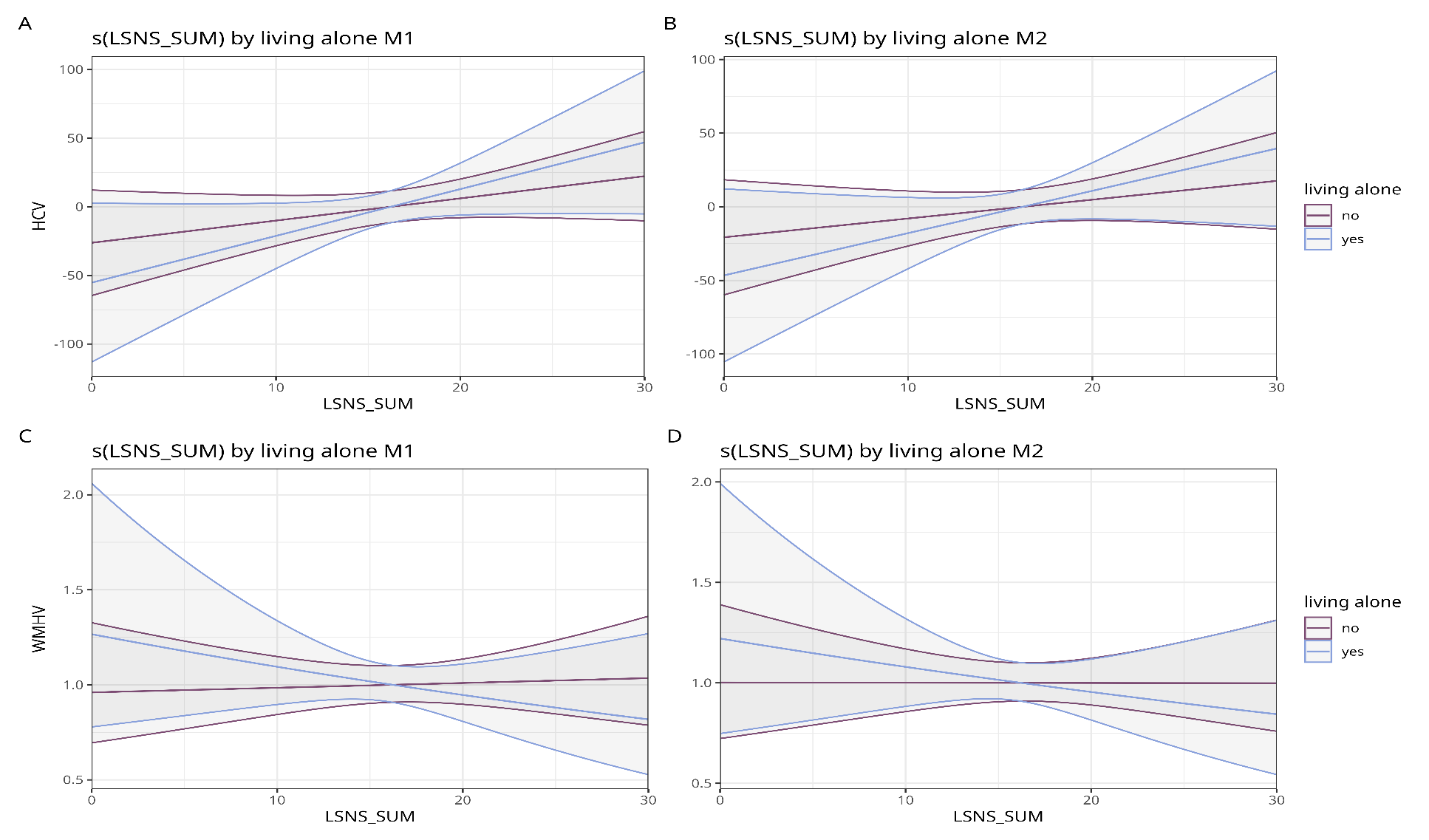
**

*Supplementary Fig. 34: A) The partial effect of Lubben Social Network Scale (LSNS) scores on hippocampal volume by cohabitation status in model 1. B) The partial effect of Lubben Social Network Scale (LSNS) scores on hippocampal volume by cohabitation status in model 2. C) The partial effect of Lubben Social Network Scale (LSNS) scores on white matter hyperintensity volume (WMHV) by cohabitation status in model 1. D) The partial effect of Lubben Social Network Scale (LSNS) scores on white matter hyperintensity volume by cohabitation status in model 2. HCV and WMHV are measured in mm3. Grey areas indicate 95% confidence intervals.*

**Fig S35**

**
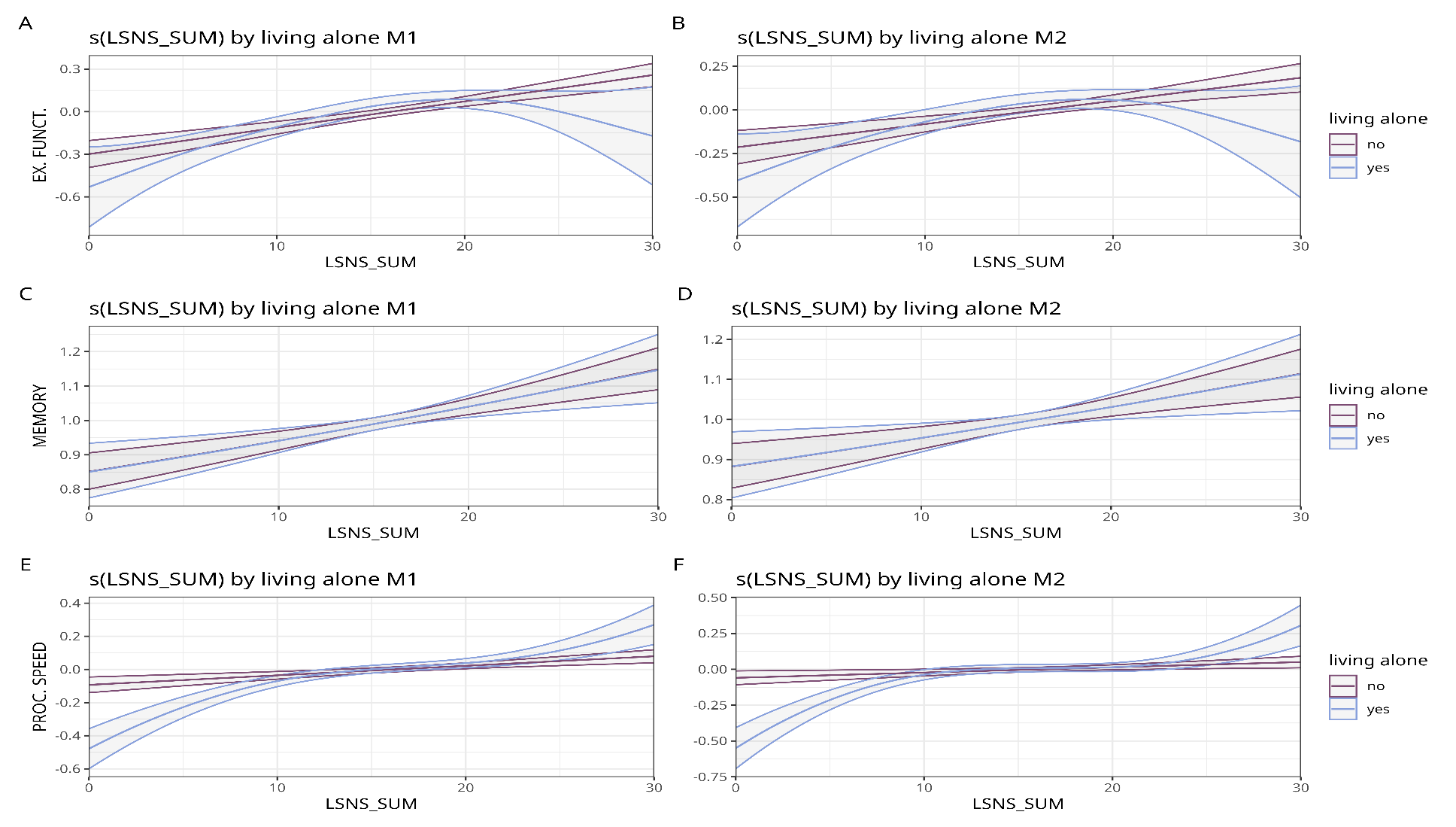
**

*Supplementary Fig. 35: A) The partial effect of Lubben Social Network Scale (LSNS) scores on executive functions by cohabitation status in model 1. B) The partial effect of Lubben Social Network Scale (LSNS) scores on executive functions by cohabitation status in model 2. C) The partial effect of Lubben Social Network Scale (LSNS) scores on memory by cohabitation status in model 1. D) The partial effect of Lubben Social Network Scale (LSNS) scores on memory by cohabitation status in model 2. E) The partial effect of Lubben Social Network Scale (LSNS) scores on processing speed by cohabitation status in model 1. F) The partial effect of Lubben Social Network Scale (LSNS) scores on processing speed by cohabitation status in model 2. Cognitive functions are measured in standard deviations. Grey areas indicate 95% confidence intervals.*

**Fig S36**

**
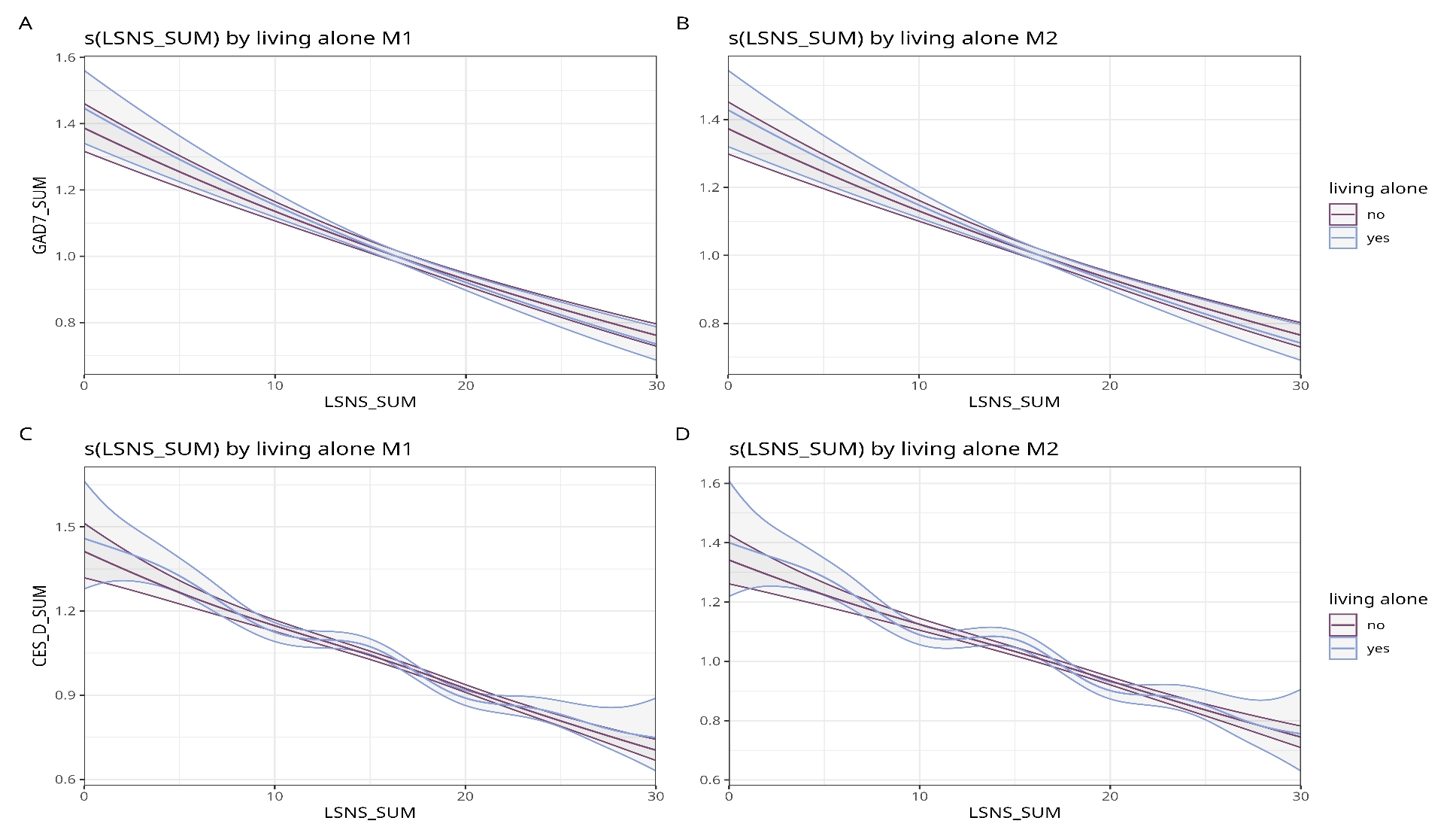
**

*Supplementary Fig. 36: A) The partial effect of Lubben Social Network Scale (LSNS) scores on anxiety symptoms by cohabitation status in model 1. B) The partial effect of Lubben Social Network Scale (LSNS) scores on anxiety symptoms by cohabitation status in model 2. C) The partial effect of Lubben Social Network Scale (LSNS) scores on depressive symptoms by cohabitation status in model 1. D) The partial effect of Lubben Social Network Scale (LSNS) scores on depressive symptoms by cohabitation status in model 2. GAD7 and CESD are measured in points on the respective questionnaire. Grey areas indicate 95% confidence intervals.*

**Fig S37**

**
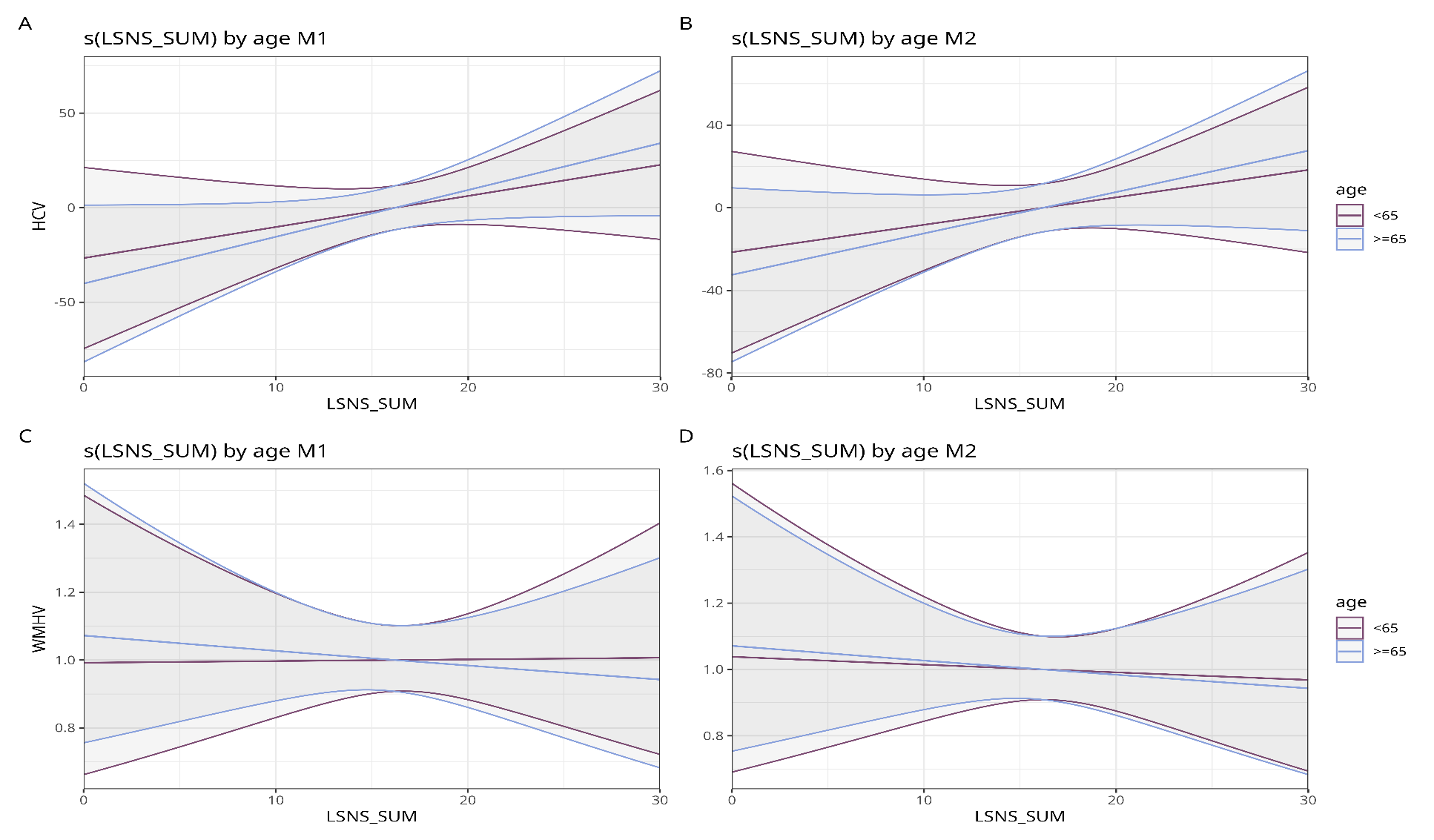
**

*Supplementary Fig. 37: A) The partial effect of Lubben Social Network Scale (LSNS) scores on hippocampal volume by working age status in model 1. B) The partial effect of Lubben Social Network Scale (LSNS) scores on hippocampal volume by working age status in model 2. C) The partial effect of Lubben Social Network Scale (LSNS) scores on white matter hyperintensity volume (WMHV) by working age status in model 1. D) The partial effect of Lubben Social Network Scale (LSNS) scores on white matter hyperintensity volume by working age status in model 2. HCV and WMHV are measured in mm3. Grey areas indicate 95% confidence intervals.*

**Fig S38**

**
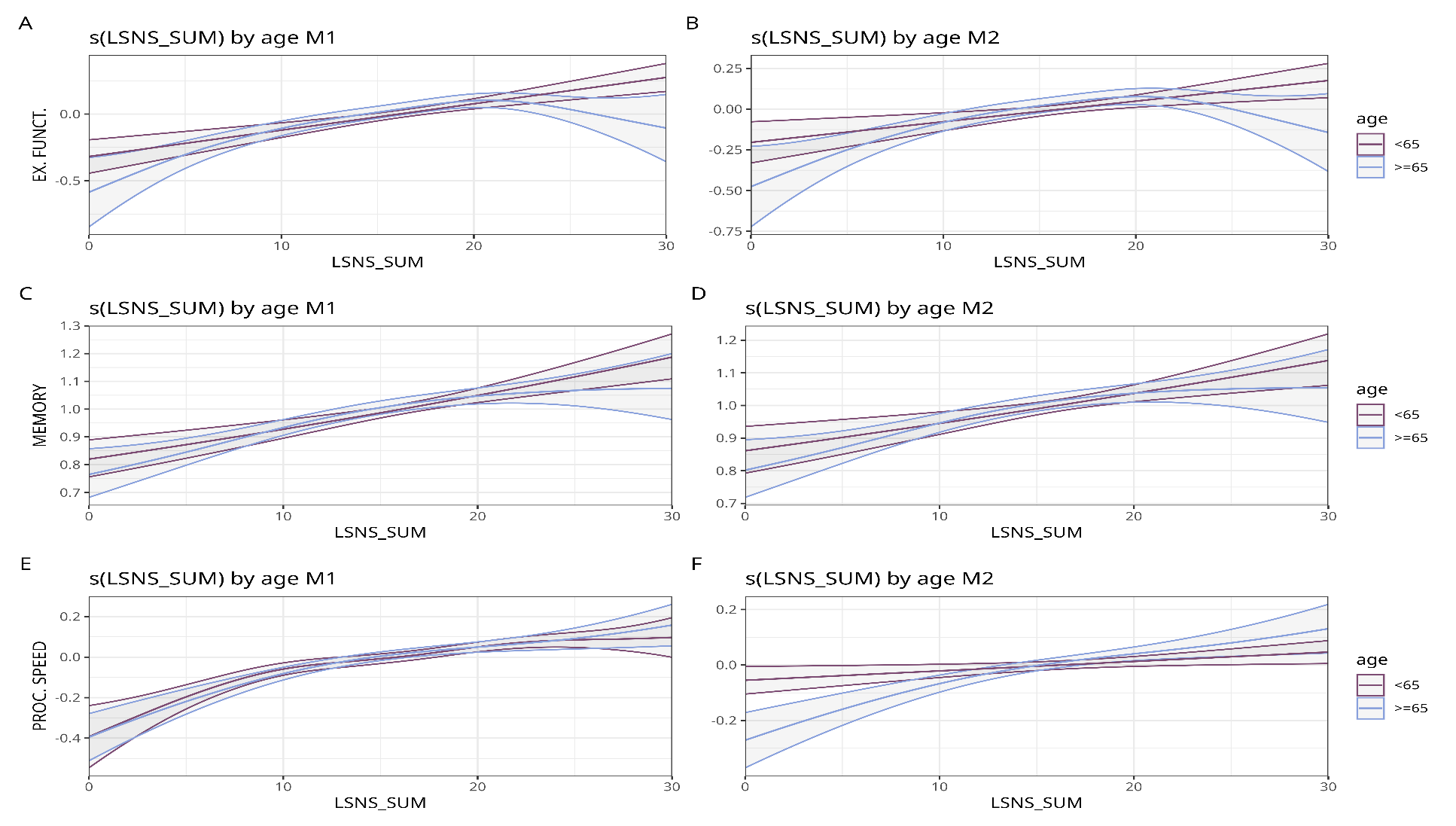
**

*Supplementary Fig. 38: A) The partial effect of Lubben Social Network Scale (LSNS) scores on executive functions by working age status in model 1. B) The partial effect of Lubben Social Network Scale (LSNS) scores on executive functions by working age status in model 2. C) The partial effect of Lubben Social Network Scale (LSNS) scores on memory by working age status in model 1. D) The partial effect of Lubben Social Network Scale (LSNS) scores on memory by working age status in model 2. E) The partial effect of Lubben Social Network Scale (LSNS) scores on processing speed by working age status in model 1. F) The partial effect of Lubben Social Network Scale (LSNS) scores on processing speed by working age status in model 2. Cognitive functions are measured in standard deviations. Grey areas indicate 95% confidence intervals.*

**Fig S39**

**
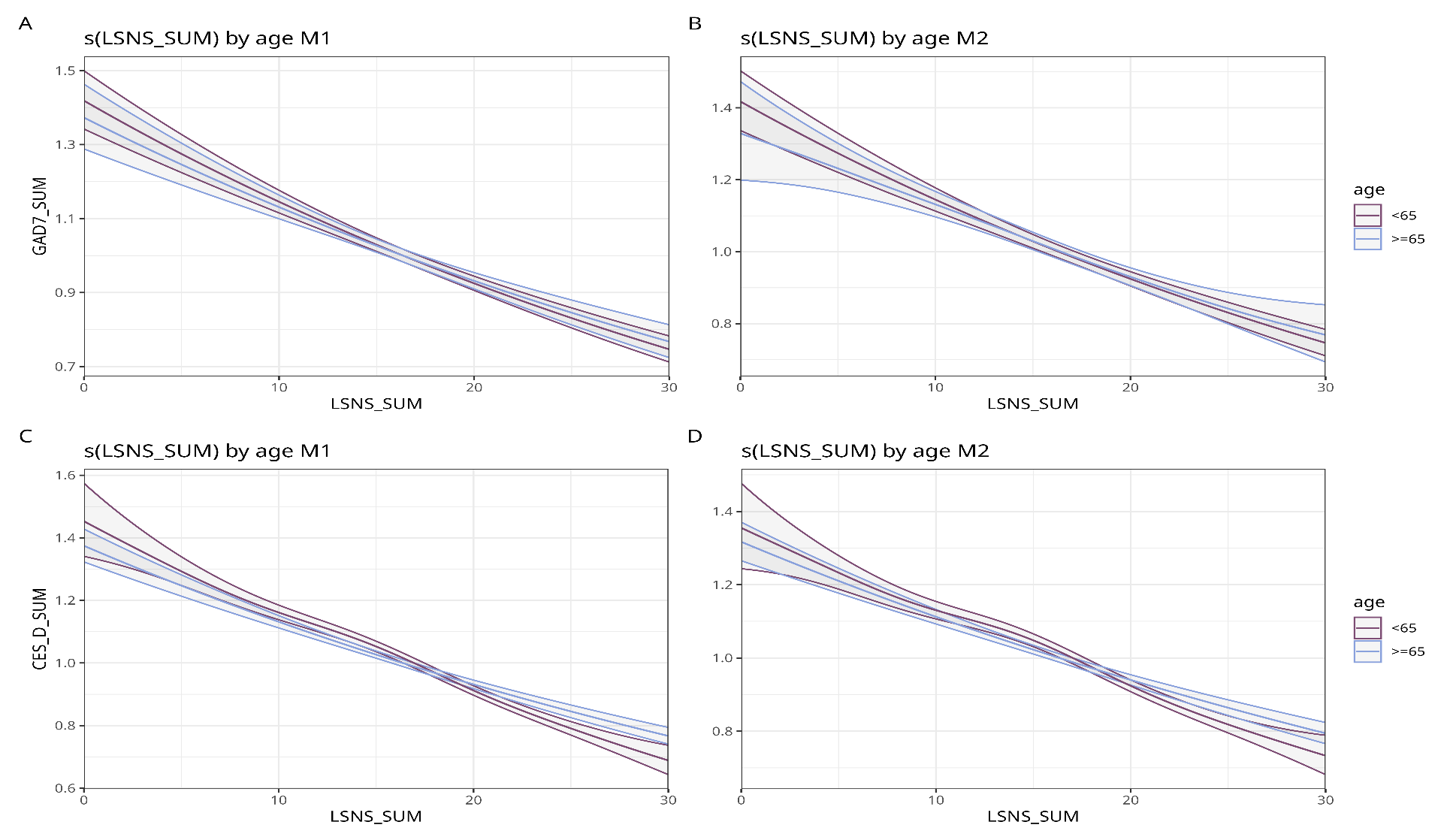
**

*Supplementary Fig. 39: A) The partial effect of Lubben Social Network Scale (LSNS) scores on anxiety symptoms by working age status in model 1. B) The partial effect of Lubben Social Network Scale (LSNS) scores on anxiety symptoms by working age status in model 2. C) The partial effect of Lubben Social Network Scale (LSNS) scores on depressive symptoms by working age status in model 1. D) The partial effect of Lubben Social Network Scale (LSNS) scores on depressive symptoms by working age status in model 2. GAD7 and CESD are measured in points on the respective questionnaire. Grey areas indicate 95% confidence intervals.*

**Fig S40**

***
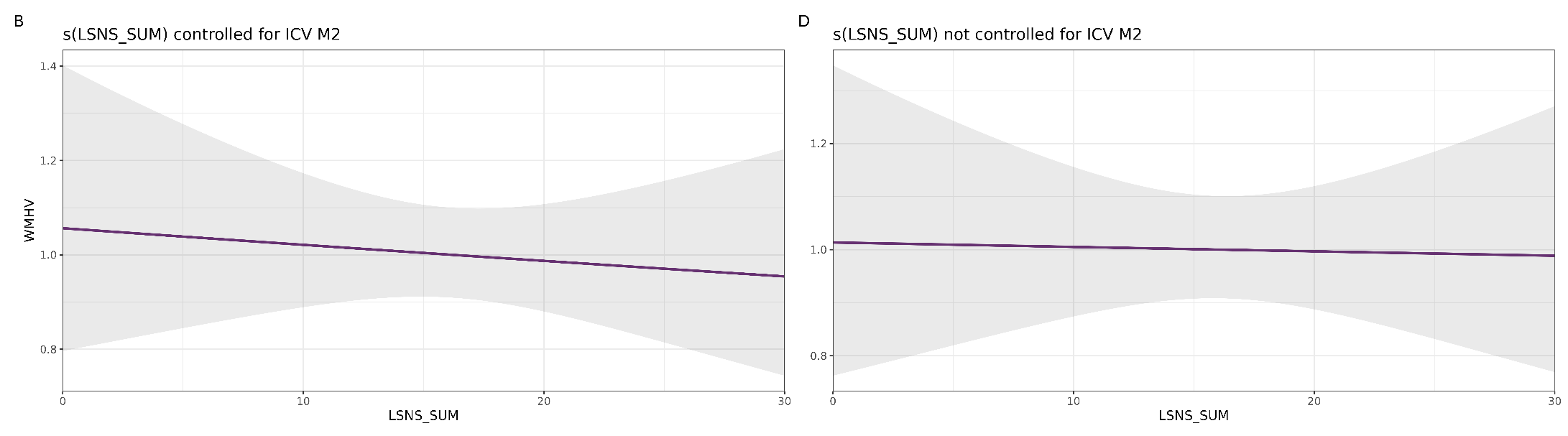
***

*Supplementary Fig. 40: Comparison of models with and without intracranial volume (ICV) as a control variable in the 1st imputed dataset. A) Partial effect of Lubben Social Network Sclae (LSNS) scores on white matter hyperintensity volume (WMHV) controlled for ICV. B) Partial effect of Lubben Social Network Sclae (LSNS) scores on white matter hyperintensity volume (WMHV) not controlled for ICV. WMHV is measured in mm3. Grey areas indicate 95% confidence intervals.*

Supplementary tables

*Supplementary table 1: Results of unweighted models*



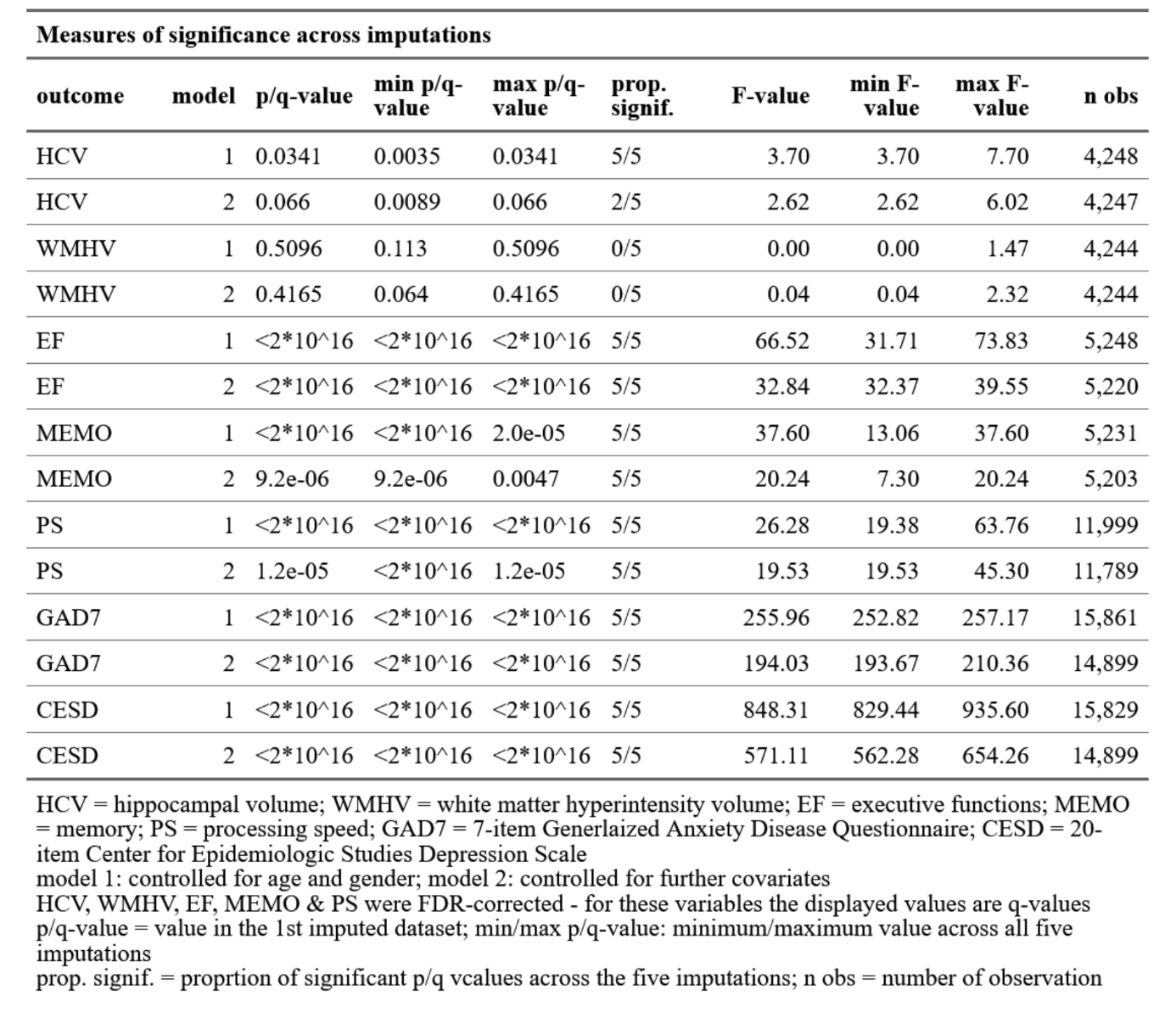


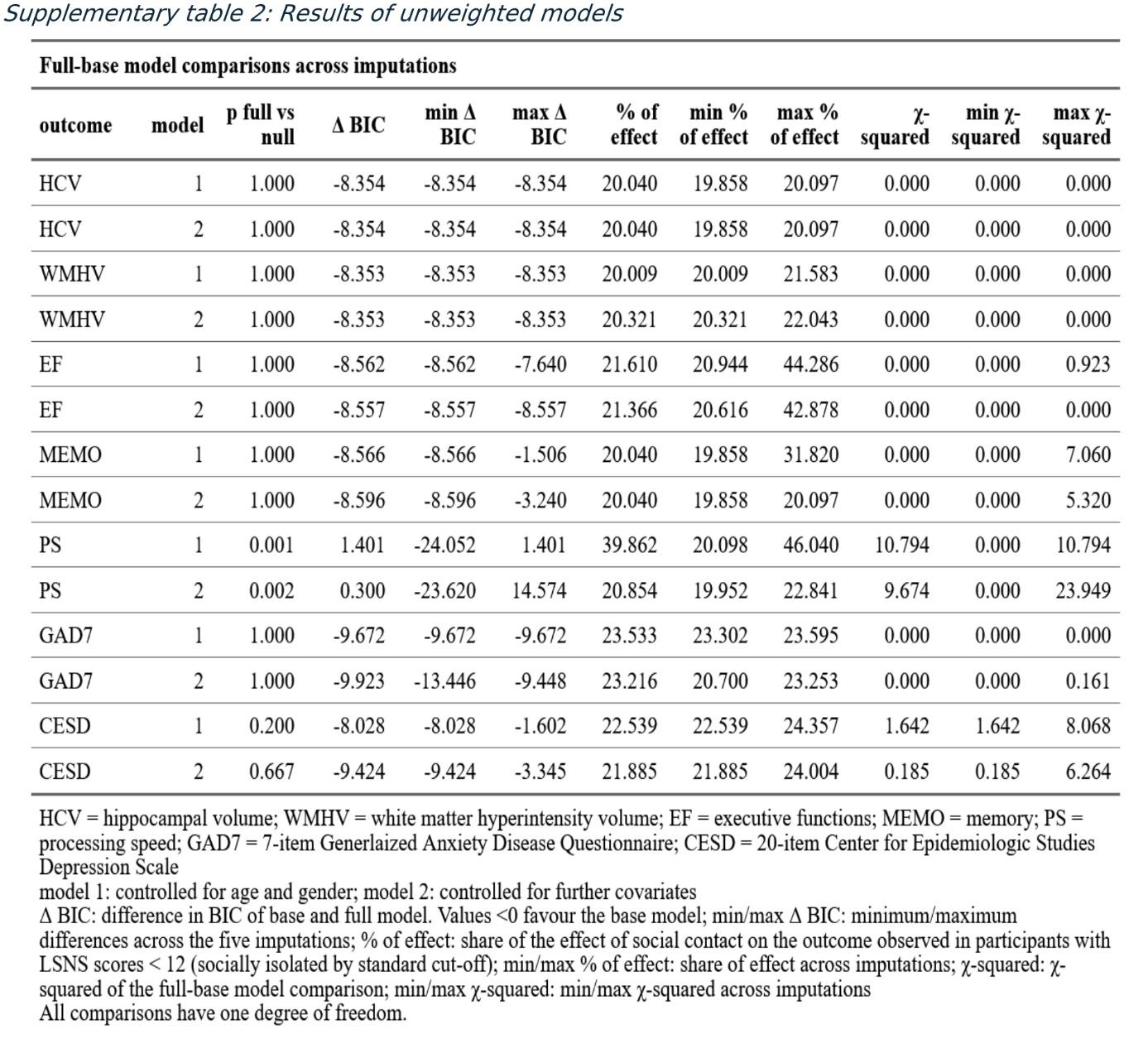


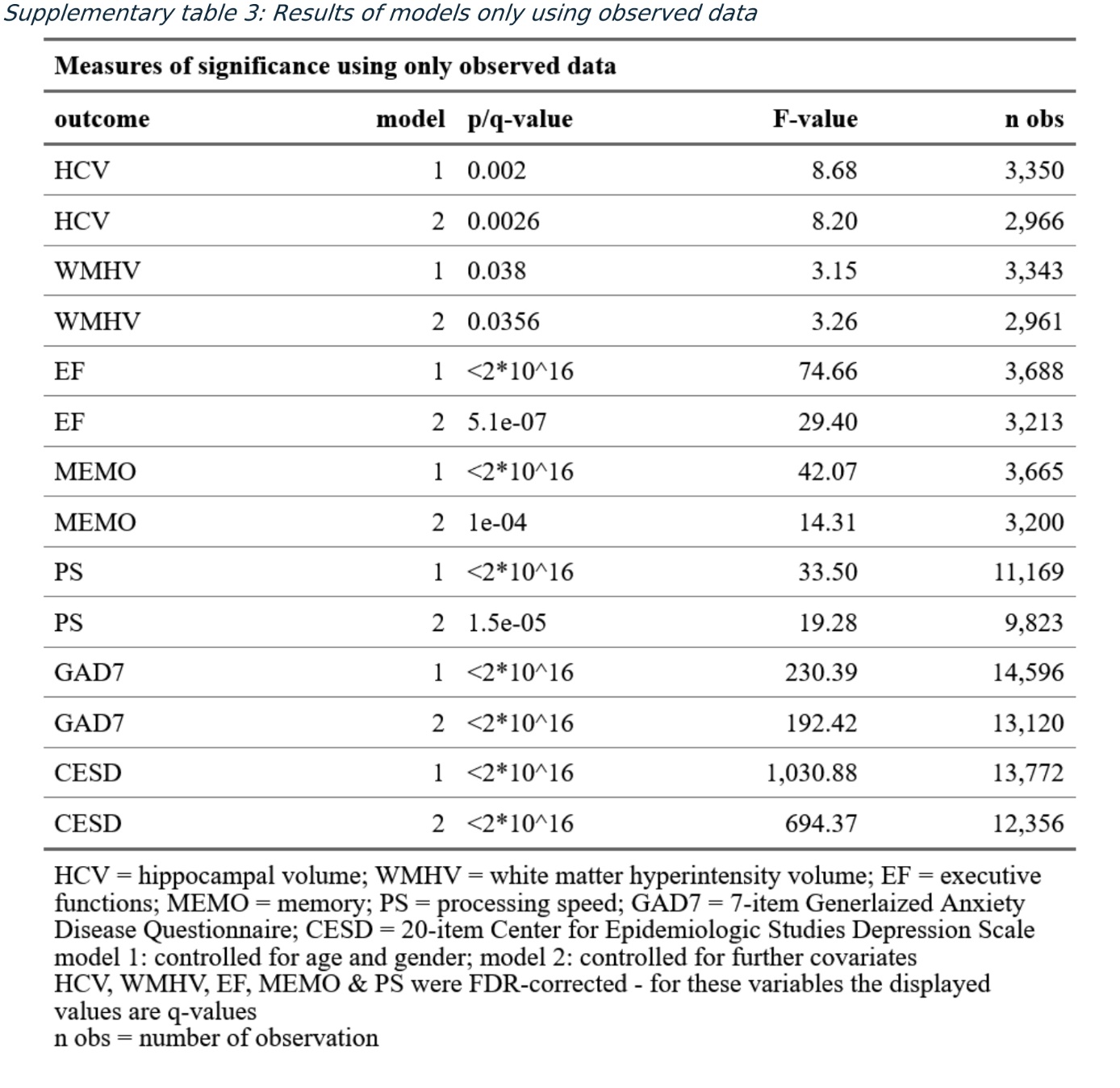


*Supplementary table 4: Results of models using only observed data**
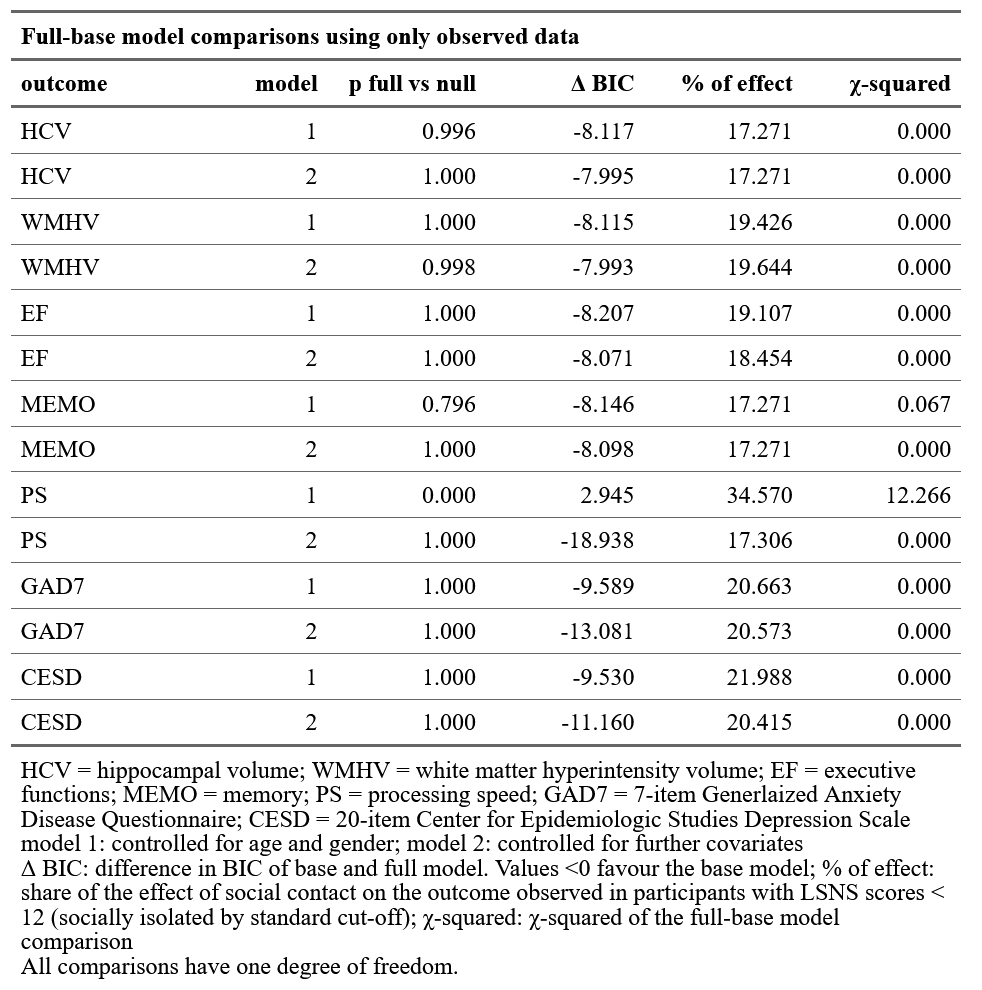
*
